# Novel species and new host records of *Apiospora* and *Nigrospora* (*Amphisphaeriales*, *Apiosporaceae*) from Yunnan-Guizhou Plateau, China

**DOI:** 10.3897/imafungus.17.177246

**Published:** 2026-04-24

**Authors:** Xing-Chang Wang, Rui-Nan Zhang, Chada Norphanphoun, Si-Bo Wang, Meng-Ting Zou, Shi-Qi Guo, Jing-E Sun, Jia-Ping Wang, Nalin N. Wijayawardene, Kevin D. Hyde, Yong Wang, Feng-Quan Liu

**Affiliations:** 1 College of Agriculture, Key Laboratory of Agricultural Microbiology of Guizhou Province, Guizhou University, Guiyang 550025, China Center of Excellence in Fungal Research, Mae Fah Luang University Chiang Rai Thailand; 2 Center for Yunnan Plateau Biological Resources, Protection and Utilization & Yunnan International Joint Laboratory of Fungal Sustainable Utilization in South and Southeast Asia, College of Biology and Food Engineering, Qujing Normal University, Qujing, Yunnan, 655011, China The Institute of Biotechnology and Genetic Engineering, Chulalongkorn University Bangkok Thailand; 3 Department of Bioprocess Technology, Faculty of Technology, Rajarata University of Sri Lanka, Mihintale, Sri Lanka College of Biology and Food Engineering, Qujing Normal University Qujing China; 4 Faculty of Graduate Studies, Sabaragamuwa University of Sri Lanka, Belihuloya, Sri Lanka College of Science, King Saud University Riyadh Saudi Arabia; 5 4High-Value Food from Mushrooms and Bioactive Plants in the Green Economy Value Chain Research Group, The Institute of Biotechnology and Genetic Engineering, Chulalongkorn University, Bangkok, Thailand College of Agriculture, Key Laboratory of Agricultural Microbiology of Guizhou Province, Guizhou University Guiyang China; 6 Department of Botany and Microbiology, College of Science, King Saud University, P.O. Box 22452, Riyadh 11495, Saudi Arabia Faculty of Technology, Rajarata University of Sri Lanka Mihintale Sri Lanka; 7 Center of Excellence in Fungal Research, Mae Fah Luang University, Chiang Rai 57100, Thailand Faculty of Graduate Studies, Sabaragamuwa University of Sri Lanka Belihuloya Sri Lanka

**Keywords:** *Arthrinium*-like, *
Ascomycota
*, multilocus analysis, phylogeny, *
Sordariomycetes
*, taxonomy

## Abstract

The family *Apiosporaceae* is a cosmopolitan family of fungi that occurs as endophytes, pathogens, and saprobes. Due to the morphological similarity of species within this family, both morphological and phylogenetic analyses are required to determine their taxonomic status. In this study, eleven specimens were collected from Yunnan and Guizhou provinces in China. Morphological and phylogenetic analyses based on ITS, LSU, *tef1-α*, and *tub2* sequence data were performed to identify *Apiospora* (*A.*) and *Nigrospora* (*N.*) isolates. Five new species are described: *A. huaxiensis***sp. nov**., *A. nanmingensis***sp. nov**., *A. qingzhenensis***sp. nov**., *A. tongrenensis***sp. nov**., and *Nigrospora neosaccharicola***sp. nov**. Six new host record species are reported: *A. locuta-pollinis, A. setariae*, *N. chinensis*, *N. endophytica, N. osmanthi*, and *N. sphaerica*. Furthermore, *A. mediterranea***syn. nov**. is synonymized under *A. hispanica*, and *A. euphorbiae***syn. nov**., *A. magnispora***syn. nov**., and *A. malaysiana***syn. nov**. are reduced to synonyms of *A. vietnamensis* based on morphological comparison and phylogenetic analysis. In addition, *Apiospora sinensis* is reinstated, with *Arthrinium sinense* (*Ar. sinense*) **syn. nov**. treated as its synonym. Statistical analysis of host preferences indicates that *Poaceae* is the dominant host family. Analysis of their regional distributions showed that *Apiospora* is more prevalent in Europe, while *Nigrospora* is more widespread in Asia. This study refines the taxonomy and expands the phylogenetic understanding of *Apiosporaceae* in China.

## Introduction

The family *Apiosporaceae* was established by Hyde et al. (1998) to accommodate *Apiospora* Sacc., together with *Appendicospora* K.D. Hyde, *Dictyoarthrinium* S. Hughes, *Endocalyx* Berk. & Broome, *Scyphospora* L.A. Kantsch., and *Spegazzinia* Sacc., based on distinctive morphological characteristics, particularly the characters of multilocular ascomata and *Arthrinium*-like basauxic conidiogenous cell (Senanayake et al. 2015; Li et al. 2023; Liu et al. 2024b). Phylogenetic analyses of LSU and SSU sequence data subsequently placed the family within *Xylariales* (Smith 2003), a position later confirmed by Crous and Groenewald (2013), who demonstrated that *Apiosporaceae* and *Amphisphaeriaceae* form sister clades. Wijayawardene et al. (2020) subsequently reassigned *Apiosporaceae* to *Amphisphaeriales* (*Ascomycota*, *Sordariomycetes*) (Hyde et al. 2024b), where it is currently classified. Over time, molecular data have refined the generic boundaries within the family. Tanaka et al. (2015) transferred *Spegazzinia* to *Didymosphaeriaceae* based on multigene phylogenetic evidence, while Wang et al. (2017) included *Nigrospora* Zimm. within *Apiosporaceae*. Samarakoon et al. (2020) further transferred *Dictyoarthrinium* to *Didymosphaeriaceae* based on its close relationship with *Spegazzinia* in both morphology and molecular phylogeny. Konta et al. (2021) placed *Endocalyx* in *Cainiaceae*, and Samarakoon et al. (2022) established *Appendicosporaceae* to accommodate *Appendicospora* as an independent lineage. As a result of these revisions, *Apiosporaceae* is now circumscribed to three well-established genera, *Apiospora*, *Arthrinium* Kunze, and *Nigrospora* (Wang et al. 2017; Pintos and Alvarado 2021; Hyde et al. 2024b; Mukhopadhyay et al. 2025). These genera are morphologically and phylogenetically related but differ in their diagnostic reproductive characteristics. *Apiospora* is the sexually typified genus, producing multilocular ascomata beneath stromata and exhibiting *Arthrinium*-like basauxic conidiogenesis, whereas *Arthrinium* was for a period considered the asexual morph linked to *Apiospora* (Kirk et al. 2008). The genus *Apiospora*, once treated as a synonym of *Arthrinium*, is now recognized as an independent lineage within *Apiosporaceae* based on multilocus phylogenetic evidence (Pintos and Alvarado 2021; Han et al. 2024; Liu et al. 2024b; Yan and Zhang 2024; Chang et al. 2025; Yu et al. 2025). For decades, both names were used interchangeably until multilocus phylogenetic analyses demonstrated that *Apiosporasensu stricto* and *Arthriniumsensu stricto* represent two distinct but closely related lineages within *Apiosporaceae* (Pintos and Alvarado 2021). Although both genera share morphological similarities, they differ in host range, distribution, and conidial morphology; *Apiospora* conidia are generally rounded in face view and lenticular in side view, while those of *Arthrinium* are more variable, including angular, curved, fusiform, globose, and polygonal forms (Zeng et al. 2022). Species of *Apiospora* have been found worldwide, ranging from tropical and subtropical regions to Mediterranean, temperate, and cold climates, while *Arthrinium* s.str. is quite rarely reported in tropical and subtropical habitats (Pintos and Alvarado 2021; Liu et al. 2023a). Additionally, *Apiospora* has a broader host range, whereas *Arthrinium* s.str. specimens are exclusively associated with plants from the *Cyperaceae* and *Juncaceae* (Pintos and Alvarado 2021)*. Nigrospora* forms the third major lineage, phylogenetically allied to the *Apiospora*–*Arthrinium* clade, and is characterized by spherical to subspherical conidiogenous cells producing black, globose to subglobose conidia (Zhang et al. 2024a; Zou et al. 2024). *Nigrospora* is another important genus comprising endophytic, saprobic, and pathogenic species that can cause diseases in economically significant plants (Zou et al. 2024). Despite their morphological distinctness, extensive convergence in conidial size, shape, and development has historically obscured genus boundaries, underscoring the necessity of molecular approaches for accurate identification and phylogenetic resolution within the family (Wang et al. 2017; Pintos and Alvarado 2021; de Queiroz Brito et al. 2023).

Members of *Apiosporaceae* are ecologically versatile, functioning as endophytes, saprobes, and plant pathogens on numerous hosts worldwide (Liao et al. 2023; Liu et al. 2023a). Several species are of agricultural and ecological importance, including *A. arundinis*, associated with root rot of *Pseudostellaria heterophylla* (Xiao et al. 2024), and *N. aurantiaca*, causing leaf spots on *Myrica rubra*, *Pandanus amaryllifolius*, and *Castanea mollissima* (Luo et al. 2020; Khoo et al. 2022; Fu et al. 2025). Moreover, most species in this family can produce secondary metabolites, demonstrating strong biological activity (Xu et al. 2022; Overgaard et al. 2023). Their ecological versatility, morphological plasticity, and close evolutionary relationships make *Apiosporaceae* one of the most taxonomically complex and diverse families within *Amphisphaeriales* (Liao et al. 2023; Liu et al. 2023a).

The Yunnan-Guizhou Plateau harbors numerous potential *Apiospora* species due to its suitable climate, demonstrating strong ecological adaptability of this genus (Zeng et al. 2022; Zhang et al. 2023; Han et al. 2024; Guo et al. 2025; Yu et al. 2025). Despite significant taxonomic progress, the diversity and distribution of *Apiosporaceae* in China remain insufficiently explored, particularly in ecologically rich provinces such as Yunnan and Guizhou. To address this gap, diseased leaves and decaying stems were collected from Southwestern China, from which 35 strains were isolated. These isolates were identified and characterized through detailed morphological observations and multilocus phylogenetic analyses based on ITS, LSU, *tef1-α*, and *tub2* sequence data. Additionally, we statistically analyzed the species distribution and host range of *Apiospora* and *Nigrospora* and selected the most suitable culture medium for morphological observation by testing multiple media. The objectives of this study were to clarify the taxonomy of these fungi, describe their morphological diversity, and elucidate their phylogenetic relationships and ecological associations within these diverse ecosystems. Comprehensive descriptions, illustrations, and phylogenetic placements of the newly identified taxa are provided herein.

## Materials and methods

### Specimen collection and isolation

Samples were collected from different hosts, including *Poaceae* (bamboo), *Zea mays* L. (maize), and *Juglans regia* L. (walnut), in various localities across Southwestern China during 2023–2024. All sample collections were placed in sterile paper bags and brought back to the Plant Pathology Laboratory at Guizhou University for fungal observation and isolation. The collected materials were first examined under a stereomicroscope to observe conidiomata and conidia. Fungi were isolated using two complementary techniques depending on the specimen condition: 1) spore suspension isolation was carried out for the species exhibiting conidiomata; 2) tissue isolation was used for samples without conidiomata but exhibiting disease symptoms, both following the protocol of Senanayake et al. (2020). Subsequently, culture plates containing spore suspensions or sterilized leaf fragments were incubated at 25 °C for 24 h. A single germinated spore or hyphal tip was then transferred to potato dextrose agar (PDA) and incubated at 25 °C for approximately 10 days to obtain pure cultures. Pure cultures were transferred to fresh PDA plates for morphological observation and DNA extraction. The fungal cultures were stored in sterile water and 30% glycerol in a refrigerator at 4 °C. The specimens were dried and stored in the Herbarium of the Department of Plant Pathology, Agricultural College, Guizhou University, Guizhou, China (HGUP). The cultures were deposited in the Culture Collection at the Department of Plant Pathology, Agricultural College, Guizhou University, Guizhou, China (GUCC). Registration ID numbers were obtained from MycoBank (www.mycobank.org) for the novel species in this study.

### Morphological observation

Morphological features of the fungal isolates were examined from cultures grown on PDA, water agar (WA), synthetic low-nutrient agar (SNA), oatmeal agar (OA), cornmeal agar (CMA), and malt extract agar (MEA) for 7–14 days at 25 °C under alternating light and dark conditions. Colony characteristics, including color, texture, growth rate, and pigmentation were recorded after 7 and 14 days of incubation. After 14 days, conidia formed on the culture medium were examined and documented. The colony color recognition was performed using the color charts of Rayner (1970). Microscopic structures were observed and photographed using a Zeiss Scope 5 compound microscope (Carl Zeiss Microscopy GmbH, Jena, Germany) with an attached camera, Axioscope 5 (AxioCam 208 color). The dimensions of the different spore structures were measured using ZEN 3.0 (blue edition) software (Jena, Germany). At least 30 measurements were taken for each significant morphological structure (e.g., conidia, conidiogenous cells, asci, and ascospores) to determine size ranges and mean values. Illustrations were prepared using Adobe Photoshop CS6 (Adobe Systems Inc., USA) to assemble images and adjust brightness and contrast without altering the original structure or color.

### DNA extraction, PCR amplification, and sequencing

Genomic DNA was extracted from 7–10–day-old pure cultures grown on PDA using a BIOMIGA Fungal Genomic DNA Extraction Kit (Biomiga#GD2416, San Diego, California, USA) according to the manufacturer’S protocol. The extracted DNA was stored at – 20 °C until further use. Four loci were amplified for phylogenetic analyses: the internal transcribed spacer (ITS) region, the large subunit ribosomal RNA gene (LSU), the translation elongation factor 1-α (*tef1-α*), and the beta tubulin (*tub2*) gene. The primer pairs and amplification conditions are summarized in Suppl. material 3: table SS1. The following primer pairs were used: ITS5/ITS4 for ITS (White et al. 1990), LR0R/LR5 for LSU (Vilgalys and Hester 1990), EF1-728F/EF2 for *tef1-α* (Carbone and Kohn 1999), and Bt2a /T1/Bt2b for *tub2* (Glass and Donaldson 1995). Polymerase chain reactions (PCR) were performed in a total volume of 25 µL, containing 12.5 µL of 2 × T5 Super PCR Mix (Tsingke Biotech Co., China), 1 µL of each primer (10 µM), 1 µL of DNA template, and 9.5 µL of sterile distilled water. PCR amplifications were carried out in a thermocycler (Applied Biosystems, USA) under the conditions listed in Suppl. material 3: table SS1. The PCR products were visualized on 1% agarose gels stained with ethidium bromide, and successful amplicons were purified and sequenced by Sangon Biotech in Chengdu, China. Forward and reverse sequence reads were assembled and edited using Geneious Prime v2023.2.2 (Biomatters Ltd., New Zealand). Consensus sequences were deposited in GenBank, and accession numbers are provided in Table 1.

**Table 1.** GenBank accession numbers of the phylogenetic analysis in this study. (Species marked in red are new species discovered in this study, while those marked in blue are species for which new host records were identified in this study or species for which the taxonomic name has changed). “T” indicates the type strain.

Taxa	Strain Numbers	GenBank Accession Numbers				Citation
		ITS	LSU	*tub*2	*tef*1*–α*	
*Apiospora acutiapica*	KUMCC 20-0210T	MT946343	MT946339	MT947366	MT947360	Senanayake et al. 2020
*A. acutiapica*	KUMCC 20-0209T	MT946342	MT946338	MT947365	MT947359	Senanayake et al. 2020
*A. adinandrae*	SAUCC 1282B-1T	OR739431	OR739572	OR757128	OR753448	Liu et al. 2024b
*A. adinandrae*	SAUCC 1282B-2	OR739432	OR739573	OR757129	OR753449	Liu et al. 2024b
*A. agari*	KUC21333T	MH498520	–	MH498478	MH544663	Kwon et al. 2021
*A. agari*	KUC21361	MH498519	–	MH498477	MN868914	Kwon et al. 2021
*A. agari*	KUC21364	MH498516	–	MH498474	MN868917	Kwon et al. 2021
*A. ananasi*	MFLU 23-0236T	OR438409	OR438876	OR538086	OR500338	Tian et al. 2024
*A. ananasi*	MFLU 23-0101	OR438410	OR438877	OR538085	OR500339	Tian et al. 2024
*A. anshunensis*	GMB6208T	PQ874006	PQ860451	PQ863977	PQ826947	Habib et al. 2025
*A. anshunensis*	GMB6207	PQ874005	PQ860450	PQ863976	PQ826946	Habib et al. 2025
*A. aquatica*	S-642T	MK828608	MK835806	–	–	Luo et al. 2019
*A. aquatica*	HKAS:131571	PQ845702	PQ819653	–	–	Shen et al. 2025
*A. aquatica*	KUNCC:23-16117	PQ845759	PV536212	–	–	Shen et al. 2025
*A. arctoscopi*	KUC21331T	MH498529	–	MH498487	MN868918	Kwon et al. 2021
*A. arctoscopi*	KUC21344	MH498528	–	MH498486	MN868919	Kwon et al. 2021
*A. arctoscopi*	KUC21345	MH498527	–	MH498485	MN868920	Kwon et al. 2021
*A. arecacearum*	KUNCC 23-15554T	PP584683	PP584780	PP982282	PP933191	Dissanayake et al. 2024
*A. arecacearum*	HKAS 133085	PP584685	PP584782	PP982284	–	Dissanayake et al. 2024
*A. arecacearum*	KUNCC 23-15555	PP584684	PP584781	PP982283	PP933192	Dissanayake et al. 2024
*A. armeniaca*	SAUCC DL1831T	OQ592540	OQ615269	OQ613285	OQ613313	Ai et al. 2024
*A. armeniaca*	SAUCC DL1844	OQ592539	OQ615268	OQ613284	OQ613312	Ai et al. 2024
*A. arundinis*	CBS 449.92	KF144887	KF144931	KF144977	KF145019	Crous and Groenewald 2013
*A. aseptata*	KUNCC 23-14169T	OR590341	OR590335	OR634943	OR634949	Zhang et al. 2023
*A. aurea*	CBS 244.83T	AB220251	KF144935	KF144981	KF145023	Crous and Groenewald 2013
*A. babylonica*	SAUCC DL1841T	OQ592538	OQ615267	OQ613283	OQ613311	Ai et al. 2024
*A. babylonica*	SAUCC DL1864	OQ592537	OQ615266	OQ613282	OQ613310	Ai et al. 2024
*A. balearica*	CBS 145129T	MK014869	MK014836	MK017975	MK017946	Pintos et al. 2019
*A. bambusicaulis*	GUCC17.41-T	PP959151	PP959161	PP998084	PP998074	Yu et al. 2025
*A. bambusicaulis*	GUCC17.42	PP959152	PP959162	PP998085	PP998075	Yu et al. 2025
*A. bambusicola*	MFLUCC 20-0144T	MW173030	MW173087	–	MW183262	Tang et al. 2020
*A. bambusigena*	SAUCC 2446-2T	PP702396	PP711785	PP716801	PP716797	Li et al. 2024
*A. bambusigena*	SAUCC 2446-6	PP702397	PP711786	PP716802	PP716798	Li et al. 2024
*A. bambusilentiginis*	GUCC18.51-T	PP959155	PP959165	PP998088	PP998078	Yu et al. 2025
*A. bambusilentiginis*	GUCC18.52	PP959156	PP959166	PP998089	PP998079	Yu et al. 2025
*A. bambusiparasitica*	RCEF20000	OR687309	PQ530552	OR712912	PQ538537	Chang et al. 2025
*A. bambusiparasitica*	RCEF20003T	OR687306	PQ530551	OR712906	OR712911	Chang et al. 2025
*A. bambusirimae*	GUCC12.51-T	PP959153	PP959163	PP998086	PP998076	Yu et al. 2025
*A. bambusirimae*	GUCC12.52	PP959154	PP959164	PP998087	PP998077	Yu et al. 2025
*A. bawanglingensis*	SAUCC BW0444T	OR739429	OR739570	OR757126	OR753446	Liu et al. 2024b
*A. bawanglingensis*	SAUCC BW04441	OQ592551	OQ615280	OQ613302	OQ613324	Liu et al. 2024b
*A. biserialis*	CGMCC 3.20135T	MW481708	MW478885	MW522955	MW522938	Feng et al. 2021
*A. biserialis*	GZCC20-0099	MW481709	MW478886	MW522956	MW522939	Feng et al. 2021
*A. biserialis*	GZCC20-0100	MW481710	MW478887	MW522957	MW522940	Feng et al. 2021
*A. camelliae-sinensis*	LC 5007T	KY494704	KY494780	KY705173	KY705103	Wang et al. 2018
*A. camelliae-sinensis*	LC 8181	KY494761	KY494837	KY705229	KY705157	Wang et al. 2018
*A. cannae*	ZHKUCC 22-0127T	OR164901	OR164948	OR166321	OR166285	Senanayake et al. 2023
*A. cannae*	ZHKUCC 22-0139	OR164902	OR164949	OR166322	OR166286	Senanayake et al. 2023
*A. chiangraiense*	MFLUCC 21-0053T	MZ542520	MZ542524	MZ546409	–	Tian et al. 2021
*A. chiangraiense*	KUNCC 24-17543	PP584687	PP584784	–	PP933194	Dissanayake et al. 2024
*A. chromolaenae*	MFLUCC 17-1505T	MT214342	MT214436	–	MT235802	Mapook et al. 2020
*A. cordylines*	GUCC 10026	MT040105	–	MT040147	MT040126	Chen et al. 2021
*A. cordylines*	GUCC 10027T	MT040106	–	MT040148	MT040127	Chen et al. 2021
*A. coryli*	CFCC 58978T	OR125564	OR133586	OR139978	OR139974	Li et al. 2023
*A. coryli*	CFCC 58979	OR125565	OR133587	OR139979	OR139975	Li et al. 2023
*A. cyclobalanopsidis*	CGMCC 3.20136T	MW481713	MW478892	MW522962	MW522945	Feng et al. 2021
*A. cyclobalanopsidis*	GZCC20-0103	MW481714	MW478893	MW522963	MW522946	Feng et al. 2021
*A. cylindrica*	CC.BYG98.1T	PP407917	PP407730	–	–	Direct Submission
*A. dehongensis*	GMBCC1011T	PQ111494	PQ111483	PQ463974	PQ464025	Han et al. 2024
*A. dehongensis*	GMBCC1012	PQ111495	PQ111484	PQ463975	PQ464026	Han et al. 2024
*A. dematiacea*	KUNCC 23-14202T	NR_190281	OR590339	OR634948	OR634953	Zhang et al. 2023
*A. dendrobii*	MFLUCC 14-0152T	NR_189847	MZ463192	–	–	Ma et al. 2022
*A. descalsii*	CBS 145130T	MK014870	MK014837	MK017976	MK017947	Pintos et al. 2019
*A. dichotomanthi*	LC 4950T	KY494697	KY494773	KY705167	KY705096	Wang et al. 2018
*A. dichotomanthi*	LC8175	KY494755	KY494831	KY705223	KY705151	Wang et al. 2018
*A. dichotomanthi*	LC8176	KY494756	KY494832	KY705224	KY705152	Wang et al. 2018
*A. dicranopteridis*	KUNCC 23-14171T	NR_190280	OR590336	OR634944	OR634950	Zhang et al. 2023
*A. dicranopteridis*	KUNCC 23-14177	OR590343	OR590337	OR634945	OR634951	Zhang et al. 2023
*A. dongyingensis*	SAUCC 0302T	OP563375	OP572424	OP573270	OP573264	Liu et al. 2023a
*A. dongyingensis*	SAUCC 0303	OP563374	OP572423	OP573269	OP573263	Liu et al. 2023a
*A. elliptica*	ZHKUCC 22-0131T	OR164905	OR164952	OR166323	OR166284	Senanayake et al. 2023
*A. elliptica*	ZHKUCC 22-0140	OR164906	OR164953	OR166324	–	Senanayake et al. 2023
*A. endophytica*	ZHKUCC 23-0006T	OQ587996	OQ587984	OQ586075	OQ586062	Liao et al. 2023
*A. endophytica*	ZHKUCC 23-0007	OQ587997	OQ587985	OQ586076	OQ586063	Liao et al. 2023
*A. esporlensis*	CBS 145136T	MK014878	MK014845	MK017983	MK017954	Pintos et al. 2019
*A. fermenti*	KUC21289T	MF615226	MF615213	MF615231	MH544667	Kwon et al. 2021
*A. fermenti*	KUC21288	MF615230	MF615217	MF615235	MH544668	Kwon et al. 2021
*A. fujianensis*	CGMCC3.25647T	PP159026	PP159034	PP488470	PP488454	Zhao et al. 2024
*A. fujianensis*	CGMCC3.25648	PP159027	PP159035	PP488471	PP488455	Zhao et al. 2024
*A. fuzhouensis*	CGMCC3.25649T	PP159028	PP159036	PP488468	PP488456	Zhao et al. 2024
*A. fuzhouensis*	CGMCC3.25650	PP159029	PP159037	PP488469	PP488457	Zhao et al. 2024
*A. gaoyouensis*	CFCC 52301T	MH197124	–	MH236789	MH236793	Jiang et al. 2018
*A. gaoyouensis*	CFCC 52302	MH197125	–	MH236790	MH236794	Jiang et al. 2018
*A. garethjonesii*	KUMCC 16-0202T	KY356086	KY356091	–	–	Dai et al. 2016
*A. garethjonesii*	SICAUCC 22-0027	ON228603	ON228659	ON237651	–	Direct Submission
*A. garethjonesii*	SICAUCC 22-0028	ON228606	ON228662	ON237654	–	Direct Submission
*A. gelatinosa*	KHAS 11962T	MW481706	MW478888	MW522958	MW522941	Feng et al. 2021
*A. globosa*	KUNCC 23-14210T	NR_190282	OR590340	–	OR634954	Zhang et al. 2023
*A. globosa*	GMBCC1021	PQ111502	PQ111491	–	PQ464027	Han et al. 2024
*A. gongcheniae*	YNE00465T	PP033259	PP033102	PP034691	PP034683	Yan and Zhang 2024
*A. gongcheniae*	YNE00565	PP033260	PP033103	PP034692	PP034684	Yan and Zhang 2024
*A. guangdongensis*	ZHKUCC 23-0004T	OQ587994	OQ587982	OQ586073	OQ586060	Liao et al. 2023
*A. guangdongensis*	ZHKUCC 23-0005	OQ587995	OQ587983	OQ586074	OQ586061	Liao et al. 2023
*A. guiyangensis*	HKAS 102403T	MW240647	MW240577	MW775604	MW759535	Samarakoon et al. 2022
*A. guiyangensis*	HKAS:125898	OQ029540	OQ029613	OQ186446	OQ186444	Direct Submission
*A. guizhouensis*	LC 5322T	KY494709	KY494785	KY705178	KY705108	Wang et al. 2018
*A. guizhouensis*	LC 5318	KY494708	KY494784	KY705177	KY705107	Wang et al. 2018
*A. hainanensis*	SAUCC 1681T	OP563373	OP572422	OP573268	OP573262	Liu et al. 2023a
*A. hainanensis*	SAUCC 1682	OP563372	OP572421	OP573267	OP573261	Liu et al. 2023a
*A. hispanica*	IMI 326877T	AB220242	AB220336	AB220289	–	Direct Submission, Wang et al. 2018
*A. hispanica* (= *A. mediterranea)*	IMI 326875T	AB220243	AB220337	AB220290	–	Direct Submission, Wang et al. 2018
*A. hongheensis*	ZHKUCC 23-0792T	OR936320	OR936322	PP778365	PP778354	Manawasinghe et al. 2025
*A. hongheensis*	ZHKUCC 23-0793	OR936321	OR936323	PP778366	PP778355	Manawasinghe et al. 2025
*A. huaxiensis* sp. nov.	GUCC 25-0068T	PV948976	PV940519	PV954844	PX625453	This study
*A. huaxiensis* sp. nov.	GUCC 25-0069	PV948977	PV940520	PV954845	PX625454	This study
*A. huaxiensis* sp. nov.	GUCC 25-0070	PV948978	PV940521	PV954846	PX625455	This study
*A. hydei*	CBS 114990T	KF144890	KF144936	KF144982	KF145024	Crous and Groenewald 2013
*A. hydei*	GZCC 20–0113	MW481721	MW478900	–	MW522953	Feng et al. 2021
*A. hyphopodii*	MFLUCC 15-0003T	KR069110	–	–	–	Senanayake et al. 2015
*A. hyphopodii*	SICAUCC 22-0034	ON228605	ON228661	ON237653	–	Direct Submission
*A. hysterina*	ICPM 6889T	MK014874	MK014841	MK017980	MK017951	Pintos et al. 2019
*A. hysterina*	KUC21437	ON764018	ON787757	ON806632	ON806622	Kwon et al. 2022
*A. hysterina*	KUC21438	ON764019	ON787758	ON806633	ON806623	Kwon et al. 2022
*A. iberica*	CBS 145137T	MK014879	MK014846	MK017984	MK017955	Pintos et al. 2019
*A. intestini*	CBS 135835T	KR011352	KR149063	KR011350	KR011351	Crous et al. 2015
*A. italica*	CBS 145138T	MK014880	MK014847	MK017985	MK017956	Pintos et al. 2019
*A. italica*	AP29118	MK014881	MK014848	MK017986	MK017957	Pintos et al. 2019
*A. jatrophae*	AMH-9557T	JQ246355	–	–	–	Sharma et al. 2014
*A. jiangxiensis*	LC 4494	KY494690	KY494766	KY705160	KY705089	Wang et al. 2018
*A. jiangxiensis*	LC 4577T	KY494693	KY494769	KY705163	KY705092	Wang et al. 2018
*A. jinanensis*	SAUCC DL1981T	OQ592544	OQ615273	OQ613289	OQ613317	Ai et al. 2024
*A. jinanensis*	SAUCC DL2000	OQ592543	OQ615272	OQ613288	OQ613316	Ai et al. 2024
*A. jinghongensis*	GMB-W1013T	PQ140160	PQ140163	PQ463971	PQ464022	Han et al. 2024
*A. jinghongensis*	GMB-W1014	PQ140161	PQ140164	PQ463972	PQ464023	Han et al. 2024
*A. kogelbergensis*	CBS 113333T	KF144892	KF144938	KF144984	KF145026	Crous and Groenewald 2013
*A. koreana*	KUC21332T	MH498524	–	MH498482	MH544664	Kwon et al. 2021
*A. koreana*	KUNCC23-15553	PP584690	PP584787	PP982289	PP933195	Dissanayake et al. 2024
*A. lageniformis*	KUC21686T	ON764022	ON787761	ON806636	ON806626	Kwon et al. 2022
*A. lageniformis*	KUC21687	ON764023	ON787762	ON806637	ON806627	Kwon et al. 2022
*A. lageniformis*	KUC21685	ON764021	ON787760	ON806635	ON806625	Kwon et al. 2022
*A. locuta-pollinis*	LC 11683T	MF939595	–	MF939622	MF939616	Zhao et al. 2018
*A. locuta-pollinis*	SAUCC 3808	OQ592563	OQ615292	OQ613308	OQ613336	Direct Submission
*A. locuta-pollinis*	SAUCC 3807	OQ592564	OQ615293	OQ613309	OQ613337	Direct Submission
*A. locuta-pollinis*	GUCC 25-0071	PV948987	PV940530	PV954855	PX625458	This study
*A. locuta-pollinis*	GUCC 25-0072	PV948988	PV940531	PV954856	PX625459	This study
*A. longistroma*	MFLUCC_11-0481T	KU940141	KU863129	–	–	Dai et al. 2017
*A. longistroma*	MFLUCC_11-0479	KU940142	KU863130	–	–	Dai et al. 2017
*A. lophatheri*	CFCC 58975T	OR125566	OR133588	OR139980	OR139970	Li et al. 2023
*A. lophatheri*	CFCC 58976	OR125567	OR133589	OR139981	OR139971	Li et al. 2023
*A. machili*	SAUCC 1175A-4_T	OR739433	OR739574	OR757130	OR753450	Liu et al. 2024b
*A. machili*	SAUCC 1175	OQ592560	OQ615289	OQ613307	OQ613333	Liu et al. 2024b
*A. marianiae*	CBS 148710T	NR_183001	NG_149092	–	–	Pintos and Alvarado 2022
*A. marianiae*	AP18219	ON692406	ON692422	ON677186	ON677180	Pintos and Alvarado 2022
*A. marianiae*	AP301119	ON692407	ON692423	ON677187	ON677181	Pintos and Alvarado 2022
*A. marii*	CBS 497.90T	AB220252	KF144947	KF144993	KF145035	Crous and Groenewald 2013
*A. marii*	CPC 18904	KF144902	KF144949	KF144994	KF145036	Crous and Groenewald 2013
*A. marii*	CBS 200.57	KF144900	KF144946	KF144992	KF145034	Crous and Groenewald 2013
*A. marina*	KUC21328T	MH498538	–	MH498496	MH544669	Kwon et al. 2022
*A. marina*	KUC21353	MH498537	–	MH498495	MN868923	Kwon et al. 2022
*A. menglaensis*	KUNCC 24-17546T	PP584693	PP584790	PP982292	PP933198	Dissanayake et al. 2024
*A. menglaensis*	KUNCC 24-17547	PP584694	PP584791	PP982293	PP933199	Dissanayake et al. 2024
*A. minutispora*	17E-042T	LC517882	–	LC518888	LC518889	Das et al. 2020
*A. mori*	MFLUCC 20-0181T	MW114313	MW114393	–	–	Tennakoon et al. 2021
*A. mori*	NCYUCC 19-0340	MW114314	MW114394	–	–	Tennakoon et al. 2021
*A. mukdahanensis*	MFLUCC 22-0056T	OP377735	OP377742	–	OP381089	Monkai et al. 2022
*A. multiloculata*	MFLUCC 21-0023T	OL873137	OL873138	–	–	Bhunjun et al. 2022
*A. mytilomorpha*	DAOM 214595T	KY494685	–	–	–	Wang et al. 2018
*A. nanmingensis* sp. nov.	GUCC 25-0087T	PV948979	PV940522	PV954847	PX625456	This study
*A. nanmingensis* sp. nov.	GUCC 25-0088	PV948980	PV940523	PV954848	PX625457	This study
*A. neobambusae*	LC 7106T	KY494718	KY494794	KY705186	KY806204	Wang et al. 2018
*A. neobambusae*	LC 7124	KY494727	KY494803	KY705195	KY806206	Wang et al. 2018
*A. neochinensis*	CFCC 53036T	MK819291	–	MK818547	MK818545	Jiang et al. 2020
*A. neochinensis*	CFCC 53037	MK819292	–	MK818548	MK818546	Jiang et al. 2020
*A. neogarethjonesii*	KUMCC 18-0192T	MK070897	MK070898	–	–	Hyde et al. 2020
*A. neogongcheniae*	YNE01248T	PP033263	PP033106	PP034695	PP034687	Yan and Zhang 2024
*A. neosubglobosa*	KUMCC 16-0203T	KY356090	KY356095	–	–	Dai et al. 2016
*A. neosubglobosa*	JHB006	KY356089	KY356094	–	–	Dai et al. 2016
*A. neosubglobosa*	GZAAS 20–0099	MW481705	MW478901	MW522969	MW522954	Feng et al. 2021
*A. obovata*	LC 4940T	KY494696	KY494772	KY705166	KY705095	Wang et al. 2018
*A. obovata*	LC 8177	KY494757	KY494833	KY705225	KY705153	Wang et al. 2018
*A. oenotherae*	CFCC 58972T	OR125568	OR133590	OR139982	OR139972	Li et al. 2023
*A. olivata*	ZY22.052 T	OR680531	OR680598	OR843234	OR858925	Zhang et al. 2024b
*A. olivata*	ZY22.053	OR680532	OR680599	OR843235	OR858926	Zhang et al. 2024b
*A. ovata*	CBS 115042T	KF144903	KF144950	KF144995	KF145037	Crous and Groenewald 2013
*A. pallidespora*	ZHKUCC 22-0129T	OR164903	OR164950	–	–	Senanayake et al. 2023
*A. paragongcheniae*	YNE00992T	PP033261	PP033104	PP034693	PP034685	Yan and Zhang 2024
*A. paragongcheniae*	YNE01259	PP033262	PP033105	PP034694	PP034686	Yan and Zhang 2024
*A. paraphaeosperma*	MFLUCC 13-0644T	KX822128	KX822124	–	–	Hyde et al. 2016
*A. phragmitis*	CPC 18900T	KF144909	KF144956	KF145001	KF145043	Crous and Groenewald 2013
*A. phragmitis*	CBS 145145	MK014889	MK014856	MK017994	MK017965	Pintos et al. 2019
*A. phyllostachydis*	MFLUCC 18-1101T	MK351842	MH368077	MK291949	MK340918	Yang et al. 2019
*A. phyllostachydis*	GZCC:20-0111	MW481717	–	MW522965	MW522949	Feng et al. 2021
*A. phyllostachydis*	GZCC:20-0112	MW481718	–	MW522966	MW522950	Feng et al. 2021
*A. piptatheri*	CBS 145149T	MK014893	MK014860	–	MK017969	Pintos et al. 2019
*A. piptatheri*	KUC21279	MF615229	–	MF615234	MH544671	Kwon et al. 2022
*A. piptatheri*	KUC21220	KT207736	–	KT207636	MH544672	Kwon et al. 2022
*A. pseudohyphopodii*	KUC21680T	ON764026	ON787765	ON806640	ON806630	Kwon et al. 2022
*A. pseudohyphopodii*	KUC21684	ON764027	ON787766	ON806641	ON806631	Kwon et al. 2022
*A. pseudoparenchymatica*	LC 7234T	KY494743	KY494819	KY705211	KY705139	Wang et al. 2018
*A. pseudoparenchymatica*	LC8173	KY494753	KY494829	KY705221	KY705149	Wang et al. 2018
*A. pseudoparenchymatica*	GZCC 20–0117	MW481719	MW478898	MW522967	MW522951	Feng et al. 2021
*A. pseudorasikravindrae*	KUMCC 20-0208T	MT946344	–	MT947367	MT947361	Senanayake et al. 2020
*A. pseudorasikravindrae*	KUMCC 20–0211	MT946345	–	MT947368	MT947362	Senanayake et al. 2020
*A. pseudosinensis*	CPC 21546T	KF144910	KF144957	–	KF145044	Crous and Groenewald 2013
*A. pseudosinensis*	SAUCC 0221	OP563377	OP572426	OP573272	OP573266	Liu et al. 2023a
*A. pseudosinensis*	SAUCC 0222	OP563376	OP572425	OP573271	OP573265	Liu et al. 2023a
*A. pseudospegazzinii*	CBS 102052T	KF144911	KF144958	KF145002	KF145045	Crous and Groenewald 2013
*A. pterosperma*	CPC 20193T	KF144913	KF144960	KF145004	KF145046	Crous and Groenewald 2013
*A. pusillisperma*	KUC21321T	MH498533	–	MH498491	MN868930	Kwon et al. 2022
*A. pusillisperma*	KUC21357	MH498532	–	MH498490	MN868931	Kwon et al. 2022
*A. qiannanensis*	RCEF7610	PQ526600	PQ530550	PQ538539	PQ538535	Chang et al. 2025
*A. qiannanensis*	RCEF7611T	PQ526599	PQ530549	PQ538538	PQ538536	Chang et al. 2025
*A. qingzhenensis* sp. nov.	GUCC 25-0077T	PV948972	PV940515	PV954840	PX625449	This study
*A. qingzhenensis* sp. nov.	GUCC 25-0078	PV948973	PV940516	PV954841	PX625450	This study
*A. qingzhenensis* sp. nov.	GUCC 25-0079	PV948974	PV940517	PV954842	PX625451	This study
*A. qingzhenensis* sp. nov.	GUCC 25-0080	PV948975	PV940518	PV954843	PX625452	This study
*A. qinlingensis*	CFCC 52303T	MH197120	–	MH236791	MH236795	Jiang et al. 2018
*A. rasikravindrae*	NFCCI 2144T	JF326454	–	–	–	Singh et al. 2012
*A. rasikravindrae*	KUC21327	MH498541	–	MH498499	MH544670	Kwon et al. 2021
*A. rasikravindrae*	KUC21351	MH498540	–	MH498498	MN868932	Wang et al. 2018
*A. rasikravindrae*	LC5449	KY494713	–	KY705182	KY705112	Wang et al. 2018
*A. rasikravindrae*	LC7115	KY494721	–	KY705189	KY705118	Wang et al. 2018
*A. sacchari*	CBS 372.67	KF144918	KF144964	KF145007	KF145049	Crous and Groenewald 2013
*A. sacchari*	CBS 30149	KF144917	KF144963	KF145006	KF145048	Crous and Groenewald 2013
*A. saccharicola*	CBS 191.73	KF144920	KF144966	KF145009	KF145051	Crous and Groenewald 2013
*A. saccharicola*	CBS 463.83	KF144921	KF144968	KF145011	KF145053	Crous and Groenewald 2013
*A. sargassi*	KUC21228T	KT207746	–	KT207644	MH544677	Kwon et al. 2022
*A. sargassi*	KUC21232	KT207750	KT207700	KT207648	MH544676	Kwon et al. 2022
*A. sasae*	CBS 146808T	MW883402	MW883797	MW890120	MW890104	Crous et al. 2021
*A. sasae*	23P495	PV384448	PV384449	PV460718	PV460719	Kim et al. 2025
*A. senecionis*	HKAS 127245T	PP584697	PP584794	–	PP993513	Dissanayake et al. 2024
*A. senecionis*	KUNCC 23-15557	PP584698	PP584795	–	PP993514	Dissanayake et al. 2024
*A. septata*	CGMCC 3.20134T	MW481711	MW478890	MW522960	MW522943	Feng et al. 2021
*A. septata*	GZCC20-0109	MW481712	MW478891	MW522961	MW522944	Feng et al. 2021
*A. serenensis*	IMI 326869T	AB220250	AB220344	AB220297	–	Direct Submission, Wang et al. 2018
*A. serenensis*	ATCC 76309	AB220240	AB220334	AB220287	–	Direct Submission, Wang et al. 2018
*A. setariae*	beilin024	MT492005	–	MT497467	MW118457	Jiang and Tian 2021
*A. setariae*	CFCC 54041T	MT492004	–	MT497466	MW118456	Jiang and Tian 2021
*A. setariae*	GUCC 25-0081	PV948989	PV940532	PV954857	PX625460	This study
*A. setariae*	GUCC 25-0082	PV948990	PV940533	PV954858	PX625461	This study
*A. setostroma*	KUMCC 19-0217T	MN528012	MN528011	–	MN527357	Jiang et al. 2019
*A. shangrilaensis*	GMBCC1019T	PQ111492	PQ111481	PQ164976	PQ164974	Han et al. 2024
*A. shangrilaensis*	GMBCC1020	PQ111493	PQ111482	PQ164977	PQ164975	Han et al. 2024
*A. sichuanensis*	HKAS 107008T	MW240648	MW240578	MW775605	MW759536	Samarakoon et al. 2022
*A. sichuanensis*	AP151120	ON692419	ON692426	ON677190	ON677184	Pintos and Alvarado 2022
*A. sichuanensis*	AP121220	ON692420	ON692427	ON677191	ON677185	Pintos and Alvarado 2022
*A. sinense* (= *Arthrinium sinense*)	HKUCC 3143	–	AY083831	–	–	Direct Submission, Réblová et al. 2016
*A. sorghi*	URM 93000T	MK371706	–	MK348526	–	Yuan et al. 2020
*A. sphaeroidea*	CC.YQ02.3T	PP407911	PP407724	–	PP496600	Direct Submission
*A. sphaerosperma*	CBS114315	KF144905	KF144952	KF144997	KF145039	Crous and Groenewald 2013
*A. sphaerosperma*	CBS114317	KF144906	KF144953	KF144998	KF145040	Crous and Groenewald 2013
*A. sphaerosperma*	CBS114318	KF144907	KF144954	KF144999	KF145041	Crous and Groenewald 2013
*A. sphaerosperma*	CBS114314	KF144904	KF144951	KF144996	KF145038	Crous and Groenewald 2013
*A. stipae*	CBS 146804T	MW883403	MW883798	MW890121	MW890082	Crous et al. 2021
*A. subglobosa*	MFLUCC 11-0397T	KR069112	KR069113	–	–	Senanayake et al. 2015
*A. subglobosa*	GMB-W1024	PQ140162	PQ140165	PQ463973	PQ464024	Han et al. 2024
*A. subrosea*	LC 7291	KY494751	KY494827	KY705219	KY705147	Wang et al. 2018
*A. subrosea*	LC 7292T	KY494752	KY494828	KY705220	KY705148	Wang et al. 2018
*A. taeanensis*	KUC21322T	MH498515	–	MH498473	MH544662	Kwon et al. 2022
*A. taeanensis*	KUC21359	MH498513	–	MH498471	MN868935	Kwon et al. 2022
*A. thailandica*	MFLUCC 15-0202T	KU940145	KU863133	–	–	Dai et al. 2017
*A. tongrenensis* sp. nov.	GUCC 25-0083T	PV948993	PV940536	PV954861	PX625462	This study
*A. tongrenensis* sp. nov.	GUCC 25-0084	PV948994	PV940537	PV954862	PX625463	This study
*A. trachycarpi*	KUNCC23-15558T	PP584701	PP584798	PP982298	PP933204	Dissanayake et al. 2024
*A. trachycarpi*	KUNCC23-15559	PP584702	PP584799	PP982299	PP933205	Dissanayake et al. 2024
*A. tropica*	MFLUCC 21-0056T	OK491657	OK491653	OK560922	–	Phukhamsakda et al. 2022
*A. ulmicola*	CFCC 57941	PP965512	–	–	PP957891	Yin and Jiang 2024
*A. ulmicola*	CFCC 57942	PP965513	–	–	PP957892	Yin and Jiang 2024
*A. vietnamensis*	IMI 99670T	KX986096	KX986111	KY019466	–	Wang et al. 2017
*A. vietnamensis* (= *A. malaysiana*)	CBS 102053T	KF144896	KF144942	KF144988	KF145030	Crous and Groenewald 2013
*A. vietnamensis* (= *A. magnispora*)	ZHKUCC 22-0001	OM728647	OM486971	OM543544	OM543543	Zhao et al. 2023
*A. Vietnamensis* (= *A. euphorbiae*)	IMI 285638b	AB220241	AB220335	AB220288	–	Direct Submission, Tian et al. 2021
*A. vietnamensis*	KUMCC 21-0428	ON426826	OP363254	OR025928	OR025967	Liu et al. 2023b
*A. vietnamensis*	KUMCC 21-0429	ON426827	OP363255	OR025929	OR025968	Liu et al. 2023b
*A. vietnamensis* (=*A. malaysiana*)	CBS 251.29	KF144897	KF144943	KF144989	KF145031	Crous and Groenewald 2013
*A. wurfbainiae*	ZHKUCC 23-0008T	OQ587998	OQ587986	OQ586077	OQ586064	Liao et al. 2023
*A. wurfbainiae*	ZHKUCC 23-0009	OQ587999	OQ587987	OQ586078	OQ586065	Liao et al. 2023
*A. xenocordella*	CBS 478.86T	KF144925	KF144970	KF145013	KF145055	Crous and Groenewald 2013
*A. xenocordella*	CBS 595.66	KF144926	KF144971	–	–	Crous and Groenewald 2013
*A. xiangxiense*	RCEF20001T	OR687308	PQ530553	OR712910	OR712909	Chang et al. 2025
*A. xiangxiense*	RCEF20002	OR687307	PQ530548	OR712908	OR712907	Chang et al. 2025
*A. yunnana*	MFLUCC 15-1002T	KU940147	KU863135	–	–	Dai et al. 2017
*A. yunnana*	DDQ 00281	KU940148	KU863136	–	–	Dai et al. 2017
*A. yunnanensis*	ZHKUCC 23-0014T	OQ588004	OQ587992	OQ586083	OQ586070	Liao et al. 2023
*A. yunnanensis*	ZHKUCC 23-0015	OQ588005	OQ587993	OQ586084	OQ586071	Liao et al. 2023
*A. zhaotongensis*	GMBCC1015T	PQ111500	PQ111489	PQ463980	PQ464016	Han et al. 2024
*A. zhaotongensis*	GMBCC1016	PQ111501	PQ111490	PQ463981	PQ464017	Han et al. 2024
*A. zhenxiongensis*	GMBCC1017T	PQ111498	PQ111487	PQ463978	PQ464018	Han et al. 2024
*A. zhenxiongensis*	GMBCC1018	PQ111499	PQ111488	PQ463979	PQ464019	Han et al. 2024
*A. xishuangbannaensis*	KUMCC 21-0695T	ON426832	OP363248	OR025930	OR025969	Liu et al. 2023b
*A. xishuangbannaensis*	KUMCC 21-0696	ON426833	OP363249	OR025931	OR025970	Liu et al. 2023b
*Arthrinium austriacum*	GZU 345004	MW208928	–	–	–	Pintos and Alvarado 2021
*Ar. caricicola*	AP23518	MK014871	MK014838	MK017977	MK017948	Pintos et al. 2019
*Ar. crenatum*	AG19066T	MW208931	MW208861	MW221923	MW221917	Pintos and Alvarado 2021
*Ar. curvatum*	AG191036	MW208935	MW208862	MW221924	–	Pintos and Alvarado 2021
*Ar. japonicum*	IFO 30500	AB220262	AB220356	AB220309	–	Direct Submission
*Ar. morthieri*	GZU 345043	MW208938	MW208864	MW221926	MW221920	Pintos and Alvarado 2021
*Ar. puccinioides*	AP26418	MK014894	MK014861	MK017998	MK017970	Pintos et al. 2019
*Ar. sporophleoides*	GZU 345102	MW208944	MW208866	MW221927	–	Pintos and Alvarado 2021
*Neoarthrinium lithocarpicola*	CFCC 54456	ON427580	ON427582	ON456914	–	Jiang et al. 2022
*Neo. lithocarpicola*	CFCC 55883	ON427581	ON427583	ON456915	–	Jiang et al. 2022
*Nigrospora anhuiensis*	QY-2	OP677969	–	PP103614	PP103590	Liu et al. 2024c
*N. aurantiaca*	LC7034	KX986093	–	KY019598	KY019394	Wang et al. 2017
*N. aurantiaca*	CGMCC 3.18130T	KX986064	–	KY019465	KY019295	Wang et al. 2017
*N. bambusae*	CGMCC 3.18327T	KY385307	–	KY385319	KY385313	Wang et al. 2017
*N. bambusae*	LC7245	KY385305	–	KY385321	KY385315	Wang et al. 2017
*N. brasiliensis*	CMM 1217	KY569630	–	MK720817	MK753272	Crous et al. 2019
*N. brasiliensis*	CMM 1214T	KY569629	–	MK720816	MK753271	Crous et al. 2019
*N. camelliae-sinensis*	CGMCC 3.18125T	KX985986	–	KY019460	KY019293	Wang et al. 2017
*N. chinensis*	CGMCC 3.18127T	KX986023	–	KY019462	KY019422	Wang et al. 2017
*N. chinensis*	LC6851	KX986049	–	KY019579	KY019450	Wang et al. 2017
*N. chinensis*	GUCC 25-0120	PX460746	–	PX512433	PX512413	This study
*N. chinensis*	GUCC 25-0121	PX460747	–	PX512434	PX512414	This study
*N. cooperae*	BRIP 72440aT	NR_185745	–	OP039540	OP039539	Tan et al. 2022
*N. cooperae*	BRIP 72531c	OP035049	–	OP039542	OP039541	Tan et al. 2022
*N. coryli*	W18T	PP218065	–	PP320372	PP461302	Wang et al. 2024
*N. covidalis*	LC158337	OK335210	–	OK431480	OK431486	Chen et al. 2022
*N. covidalis*	CGMCC 3.20538T	OK335209	–	OK431479	OK431485	Chen et al. 2022
*N. endophytica*	URM8462=A.R.M. 973T	OM265233	–	OP572420	OP572416	de Queiroz Brito et al. 2023
*N. endophytica*	URM8712=A.R.M. 687	OM265226	–	OP572418	OP572415	de Queiroz Brito et al. 2023
*N. endophytica*	GUCC 25-0122	PX460760	–	PX512447	PX512427	This study
*N. endophytica*	GUCC 25-0123	PX460761	–	PX512448	PX512428	This study
*N. falsivesicularis*	LC13553	MN215779	–	MN329943	MN264018	Raza et al. 2019
*N. falsivesicularis*	CGMCC 3.19678T	MN215778	–	MN329942	MN264017	Raza et al. 2019
*N. ficuum*	ZHKUCC 22-0125	OR164910	–	–	–	Senanayake et al. 2023
*N. ficuum*	ZHKUCC 22-0143	OR164911	–	–	–	Senanayake et al. 2023
*N. globosa*	LC12440T	MK329121	–	MK336134	MK336056	Chen et al. 2022
*N. globosa*	LC12441	MK329122	–	MK336135	MK336057	Chen et al. 2022
*N. globospora*	LC15839	OK335212	–	OK431482	OK431488	Chen et al. 2022
*N. globospora*	CGMCC 3.20539T	OK335211	–	OK431481	OK431487	Chen et al. 2022
*N. gorlenkoana*	CBS 480.73T	KX986048	–	KY019456	KY019420	Wang et al. 2017
*N. guangdongense*	ZHKUCC 24-0545	PV523285	–	PV536733	PV536735	Hongsanan et al. 2025
*N. guangdongense*	ZHKUCC 24-0546	PV523286	–	PV536734	PV536736	Hongsanan et al. 2025
*N. guangdongensis*	CFCC:53917T	MT017509	–	MT024495	MT024493	Tian et al. 2020
*N. guilinensis*	CGMCC 3.18124T	KX985983	–	KY019459	KY019292	Wang et al. 2017
*N. guilinensis*	LC7301	KX986063	–	KY019608	KY019404	Wang et al. 2017
*N. hainanensis*	CGMCC 3.18129T	KX986091	–	KY019464	KY019415	Wang et al. 2017
*N. hainanensis*	URM8714=A.R.M.967	OM265228	–	OM793057	OM642834	de Queiroz Brito et al. 2023
*N. hainanensis*	URM8715=A.R.M.968	OM265229	–	OM793058	OM642835	de Queiroz Brito et al. 2023
*N. hainanensis*	URM8717=A.R.M.972	OM265232	–	OP572419	OM642837	de Queiroz Brito et al. 2023
*N. hainanensis*	URM8719=A.R.M.976	OM265236	–	OM793060	OP572417	de Queiroz Brito et al. 2023
*N. humicola*	CFCC 56884T	ON555686	–	ON557392	ON557394	Zhang et al. 2024a
*N. humicola*	CFCC 56885	ON555687	–	ON557393	ON557395	Zhang et al. 2024a
*N. lacticolonia*	CGMCC 3.18123T	KX985978	–	KY019458	KY019291	Wang et al. 2017
*N. lacticolonia*	URM8713=A.R.M. 921	OM265227	–	OM642838	OM642833	de Queiroz Brito et al. 2023
*N. macarangae*	MFLUCC_19-0141	MW114318	–	–	–	Tennakoon et al. 2021
*N. macarangae*	NCYUCC 19-0177	MW114319	–	–	–	Tennakoon et al. 2021
*N. macarangae*	NCYUCC 19-0312	MW114320	–	–	–	Tennakoon et al. 2021
*N. magnoliae*	MFLUCC 19–0112T	MW285092	–	MW438334	–	Tennakoon et al. 2021
*N. manihoticola*	URM8461=A.R.M. 645T	OM265224	–	OM869479	OM914791	de Queiroz Brito et al. 2023
*N. marylouisemclawsiae*	BRIP 74865b	PP125567	–	PP209362	PP209361	Direct Submission
*N. mercuriadeae*	BRIP 75764a	NR_198785	–	PP712794	PP712793	Direct Submission
*N. musae*	CBS 319.34T	KX986076	–	KY019455	KY019419	Wang et al. 2017
*N. musae*	LC6385	KX986042	–	KY019567	KY019371	Wang et al. 2017
*N. neosaccharicola* sp. nov.	GUCC 25-0124T	PX460756	–	PX512443	PX512423	This study
*N. neosaccharicola* sp. nov.	GUCC 25-0125	PX460757	–	PX512444	PX512424	This study
*N. neosaccharicola* sp. nov.	GUCC 25-0126	PX460758	–	PX512445	PX512425	This study
*N. neosaccharicola* sp. nov.	GUCC 25-0127	PX460759	–	PX512446	PX512426	This study
*N. neosaccharicola* sp. nov.	GUCC 25-0128	PX460764	–	PX512451	PX512431	This study
*N. neosaccharicola* sp. nov.	GUCC 25-0129	PX460765	–	PX512452	PX512432	This study
*N. oryzae*	LC2724	KX985959	–	KY019486	KY019312	Wang et al. 2017
*N. oryzae*	LC4265	KX985994	–	KY019518	KY019335	Wang et al. 2017
*N. osmanthi*	CGMCC 3.18126T	KX986010	–	KY019461	KY019421	Wang et al. 2017
*N. osmanthi*	LC4487	KX986017	–	KY019540	KY019438	Wang et al. 2017
*N. osmanthi*	GUCC 25-0130	PX460752	–	PX512439	PX512419	This study
*N. osmanthi*	GUCC 25-0131	PX460753	–	PX512440	PX512420	This study
*N. osmanthi*	GUCC 25-0132	PX460754	–	PX512441	PX512421	This study
*N. osmanthi*	GUCC 25-0133	PX460755	–	PX512442	PX512422	This study
*N. pernambucoensis*	URM8711=A.R.M.651	OM265225	–	OM869480	OM914792	de Queiroz Brito et al. 2023
*N. pernambucoensis*	URM8463=A.R.M. 974T	OM265234	–	OM869481	OM914793	de Queiroz Brito et al. 2023
*N. philosophiae-doctoris*	CGMCC 3.20540T	OK335214	–	OK431484	OK431490	Chen et al. 2022
*N. pubeiensis*	KUNCC 23-16745	PQ553686	–	PQ613608	PQ613603	Zhang et al. 2025
*N. pyriformis*	CGMCC 3.18122T	KX985940	–	KY019457	KY019290	Wang et al. 2017
*N. pyriformis*	URM8716=A.R.M.970	OM265231	–	OM642839	OM513904	de Queiroz Brito et al. 2023
*N. rubi*	LC2698T	KX985948	–	KY019475	KY019302	Wang et al. 2017
*N. saccharicola*	LC12057	MN215789	–	MN329952	MN264028	Raza et al. 2019
*N. saccharicola*	CGMCC 3.19362T	MN215788	–	MN329951	MN264027	Raza et al. 2019
*N. sacchari-ofcinarum*	CGMCC 3.19335T	MN215791	–	MN329954	MN264030	Raza et al. 2019
*N. sacchari-ofcinarum*	LC13531	MN215792	–	MN329955	MN264031	Raza et al. 2019
*N. singularis*	CGMCC 3.19334T	MN215793	–	MN329956	MN264032	Raza et al. 2019
*N. singularis*	LC12068	MN215794	–	MN329957	MN264033	Raza et al. 2019
*N. sphaerica*	LC 7294	KX985932	–	KY019602	KY019397	Wang et al. 2017
*N. sphaerica*	LC 2705	KX985952	–	KY019479	KY019305	Wang et al. 2017
*N. sphaerica*	LC 2958	KX985966	–	KY019493	KY019319	Wang et al. 2017
*N. sphaerica*	LC 3420	KX985980	–	KY019506	KY019325	Wang et al. 2017
*N. sphaerica*	LC 3477	KX985982	–	KY019508	KY019326	Wang et al. 2017
*N. sphaerica*	LC 4174	KX985989	–	KY019513	KY019330	Wang et al. 2017
*N. sphaerica*	LC 4241	KX985990	–	KY019514	KY019331	Wang et al. 2017
*N. sphaerica*	LC 4263	KX985992	–	KY019516	KY019333	Wang et al. 2017
*N. sphaerica*	LC 4264	KX985993	–	KY019517	KY019334	Wang et al. 2017
*N. sphaerica*	LC 4274	KX985996	–	KY019520	KY019337	Wang et al. 2017
*N. sphaerica*	LC 4278	KX985998	–	KY019522	KY019339	Wang et al. 2017
*N. sphaerica*	LC 4291	KX986000	–	KY019524	KY019341	Wang et al. 2017
*N. sphaerica*	LC 4293	KX986001	–	KY019525	KY019342	Wang et al. 2017
*N. sphaerica*	LC 4303	KX986004	–	KY019528	KY019345	Wang et al. 2017
*N. sphaerica*	LC 4307	KX986005	–	KY019529	KY019346	Wang et al. 2017
*N. sphaerica*	LC 4372	KX986012	–	KY019535	KY019351	Wang et al. 2017
*N. sphaerica*	LC 4447	KX986014	–	KY019537	KY019352	Wang et al. 2017
*N. sphaerica*	LC 5932	KX986035	–	KY019557	KY019362	Wang et al. 2017
*N. sphaerica*	LC 5944	KX986036	–	KY019558	KY019363	Wang et al. 2017
*N. sphaerica*	LC 5966	KX986039	–	KY019561	KY019365	Wang et al. 2017
*N. sphaerica*	LC 6294	KX986044	–	KY019565	KY019369	Wang et al. 2017
*N. sphaerica*	LC 6969	KX986077	–	KY019584	KY019386	Wang et al. 2017
*N. sphaerica*	LC 6996	KX986085	–	KY019592	KY019390	Wang et al. 2017
*N. sphaerica*	LC 7295	KX985933	–	KY019603	KY019398	Wang et al. 2017
*N. sphaerica*	LC 7296	KX985934	–	KY019604	KY019399	Wang et al. 2017
*N. sphaerica*	LC 7298	KX985937	–	KY019606	KY019401	Wang et al. 2017
*N. sphaerica*	LC 7303	KX986065	–	KY019609	KY019405	Wang et al. 2017
*N. sphaerica*	LC 7304	KX986066	–	KY019610	KY019406	Wang et al. 2017
*N. sphaerica*	LC2839	KX985964	–	KY019491	KY019317	Wang et al. 2017
*N. sphaerica*	LC2840	KX985965	–	KY019492	KY019318	Wang et al. 2017
*N. sphaerica*	LC5901	KX986034	–	KY019556	KY019361	Wang et al. 2017
*N. sphaerica*	LC7312	KX985935	–	KY019618	KY019414	Wang et al. 2017
*N. sphaerica*	GUCC 25-0134	PX460748	–	PX512435	PX512415	This study
*N. sphaerica*	GUCC 25-0135	PX460749	–	PX512436	PX512416	This study
*N. sphaerica*	GUCC 25-0136	PX460750	–	PX512437	PX512417	This study
*N. sphaerica*	GUCC 25-0137	PX460751	–	PX512438	PX512418	This study
*N. sphaerica*	GUCC 25-0138	PX460762	–	PX512449	PX512429	This study
*N. sphaerica*	GUCC 25-0139	PX460763	–	PX512450	PX512430	This study
*N. stoneae*	BRIP 75022a	OR608744	–	OR604067	OR604065	Direct Submission
*N. tomentosae*	ZHKUCC 22-0339	PP759659	–	PP763296	PP763294	Manawasinghe et al. 2025
*N. vesicularifera*	CGMCC 3.19333T	MN215812	–	MN329975	MN264051	Raza et al. 2019
*N. vesicularifera*	URM8718=A.R.M.975	OM265235	–	OM642840	OM513905	de Queiroz Brito et al. 2023
*N. vesicularis*	LC0322	KX985939	–	KY019467	KY019296	Wang et al. 2017
*N. vesicularis*	CGMCC 3.18128T	KX986088	–	KY019463	KY019294	Wang et al. 2017
*N. weininensis*	GUCC23-0144T	PP729081	–	PP737836	PP731561	Liu et al. 2024a
*N. yunnanensis*	GUCC24-0008T	PP915796	–	PP947937	PP947933	Zou et al. 2024
*N. zimmermanii*	CBS 290.62T	KY385309	–	KY385317	KY385311	Wang et al. 2017
*N. zimmermanii*	CBS 984.69	KY385310	–	KY385322	KY385316	Wang et al. 2017

### Phylogenetic analyses

All sequences generated in this study were initially compared against the NCBI nucleotide database using the BLAST tool (https://blast.ncbi.nlm.nih.gov/) to determine closely related taxa. Representative reference sequences of related genera and species were selected for subsequent phylogenetic analyses based on BLAST results and recent taxonomic publications (Liu et al. 2024a; Yan and Zhang 2024; Zou et al. 2024; Chang et al. 2025; Yu et al. 2025). GenBank accession numbers of all sequences used in this study are listed in Table 1. Raw sequence chromatograms were trimmed and edited using the Trim Ends function in Geneious Prime v2023.2.2 (Biomatters Ltd., New Zealand), and individual loci were assembled into consensus sequences. Each locus was aligned using MAFFT v7.520 (https://mafft.cbrc.jp/alignment/server/) with default settings (Katoh et al. 2019). The resulting alignments were manually checked and trimmed using TrimAl v1.3 (Capella-Gutiérrez et al. 2009) to remove ambiguously aligned regions. Concatenated datasets were generated with PhyloSuite v1.2.3 (Zhang et al. 2020).

Phylogenetic analyses were conducted using both Maximum Likelihood (ML) and Bayesian Inference (BI) algorithms. The best-fit substitution model for each gene partition was determined using ModelFinder implemented in IQ-TREE v2.2.0 and is summarized in Suppl. material 3: table S2. ML analyses were performed with 1,000 ultrafast bootstrap replications to assess branch support. Bayesian analyses were carried out in MrBayes v3.2.7a, running two parallel analyses of four Markov Chain Monte Carlo (MCMC) chains for 5,000,000 generations, sampling every 1,000 generations, and discarding the first 25% of trees as burn-in. Phylogenetic trees were visualized in FigTree v1.4.0 (http://tree.bio.ed.ac.uk/software/figtree) and annotated in Adobe Illustrator 2023 (Adobe Systems Inc., USA) for final presentation.

### Host and geographical distribution of *Nigrospora* and *Apiospora*

To assess the host associations and geographic distribution of *Apiospora* and *Nigrospora*, species occurrence records for both genera were retrieved from the Global Biodiversity Information Facility (GBIF) database (GBIF.org, accessed 19 January 2026; Occurrence Downloads: https://doi.org/10.15468/dl.dx2b4u and https://doi.org/10.15468/dl.4ng2ze). The downloaded datasets were curated and standardized, and the following fields were extracted for analysis: GBIF ID, habitat, associated taxa (host), host family, country of collection, and accepted scientific name (Suppl. material 3: tables S3, S4). Records lacking information on habitat or associated taxa (host) were excluded from further analyses.

In total, 2,354 occurrence records for *Apiospora* and 1,785 for *Nigrospora* were obtained. Of these, 2,231 *Apiospora* records and 1,759 *Nigrospora* records contained country-level collection data and were retained for geographic analyses. Subsequent data processing, statistical analyses, and graphical visualization were conducted using R software.

## Results

### Phylogenetic analyses

**Figure 1.**
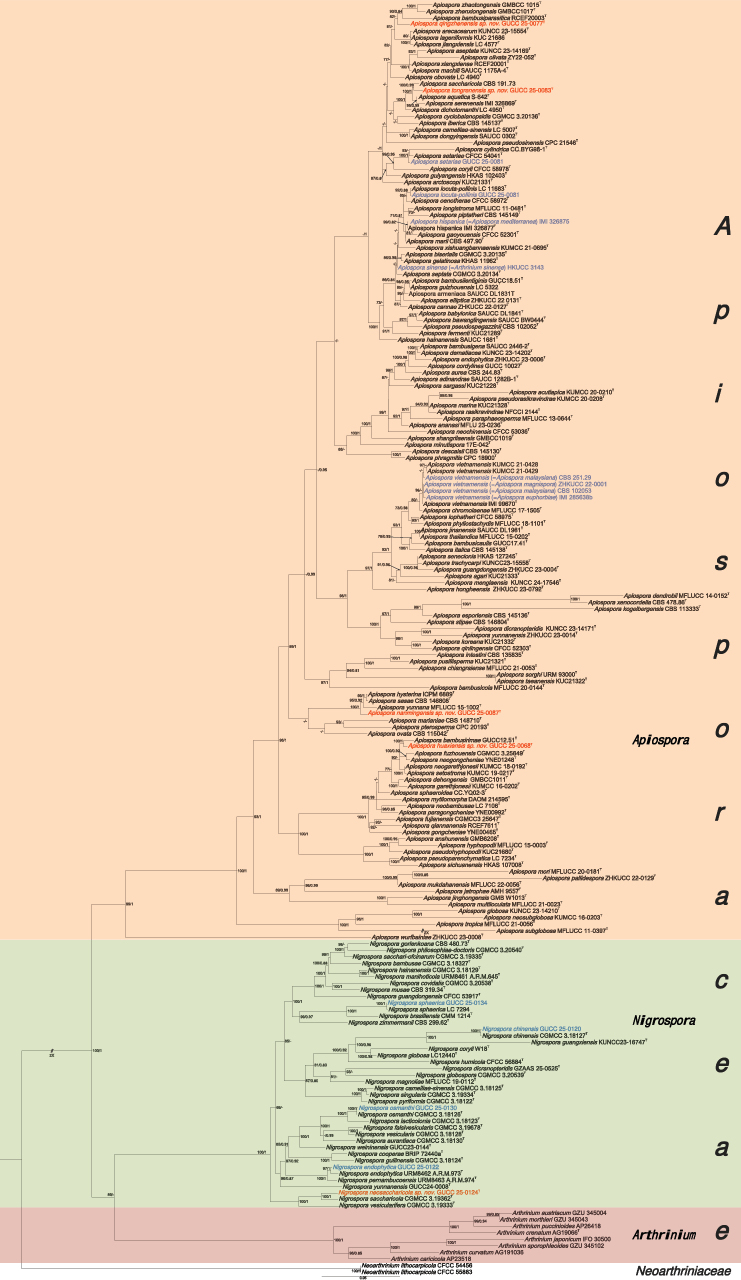
Phylogenetic tree constructed using Maximum likelihood (ML) analyses based on the multilocus alignment (ITS, LSU, *tef*1-α, and *tub2*) for *Apiosporaceae*. The outgroup is *Neoarthrinium lithocarpicola* (CFCC 54456 and CFCC 55883). Maximum likelihood bootstrap proportions ≥ 70% (left) and Bayesian inference posterior probability ≥ 0.8 (right) are indicated at nodes (ML/BI). The type strains are followed by “^T^” and bold. New species in this study are red, and new records and merged species are blue.

For family *Apiosporaceae* (Fig. 1), a concatenated alignment comprising ITS (1–615), LSU (616–1361), *tef1-α* (1362–2368), and *tub2* (2369–2613) loci was analyzed, including 194 strains with *Neoarthrinium lithocarpicola* (CFCC 54456 and CFCC 55883) designated as outgroup taxa. Eleven strains in this research were incorporated into the dataset. The ML topology served as the reference, with Bayesian posterior probabilities mapped onto corresponding branches. Both topologies were identical, and the best-scoring RAxML tree with a final ML optimization likelihood value of – 38398.438 is presented. Estimated base frequencies were as follows: A = 0.232, C = 0.258, G = 0.256, T = 0.254; substitution rates AC = 1.17641, AG = 2.69166, AT = 1.17641, CG = 1.0, CT = 4.67220, GT = 1.0. The phylogenetic tree clearly demonstrates that the three genera *Apiospora*, *Nigrospora*, and *Arthrinium* within *Apiosporaceae* form distinct and well-separated clades.

Phylogenetic analyses further revealed several instances of minimal or no genetic divergence among certain taxa; *A. hispanica* (IMI 326877) and *A. mediterranea* (IMI 326875) clustered together without detectable genetic distance. Similarly, *A. vietnamensis* (IMI 99670, KUMCC 21-0428, KUMCC 21-0429), *A. malaysiana* (CBS 102053, CBS 251.29), *A. magnispora* (ZHKUCC 22-0001T), and *A. euphorbiae* (IMI 285638b) formed a closely related cluster with little genetic divergence (ML/BI = 96/0.63). *Apiospora jinanensis* (SAUCC DL1981) and *A. thailandica* (MFLUCC 15-0202) grouped together with no genetic distance, as did *A. sorghi* (URM 93000) and *A. taeanensis* (KUC21322). Notably, *Ar. sinense* (HKUCC 3143) clusters within the *Apiospora* clade, suggesting potential taxonomic implications that may warrant further investigation.

**Figure 2.**
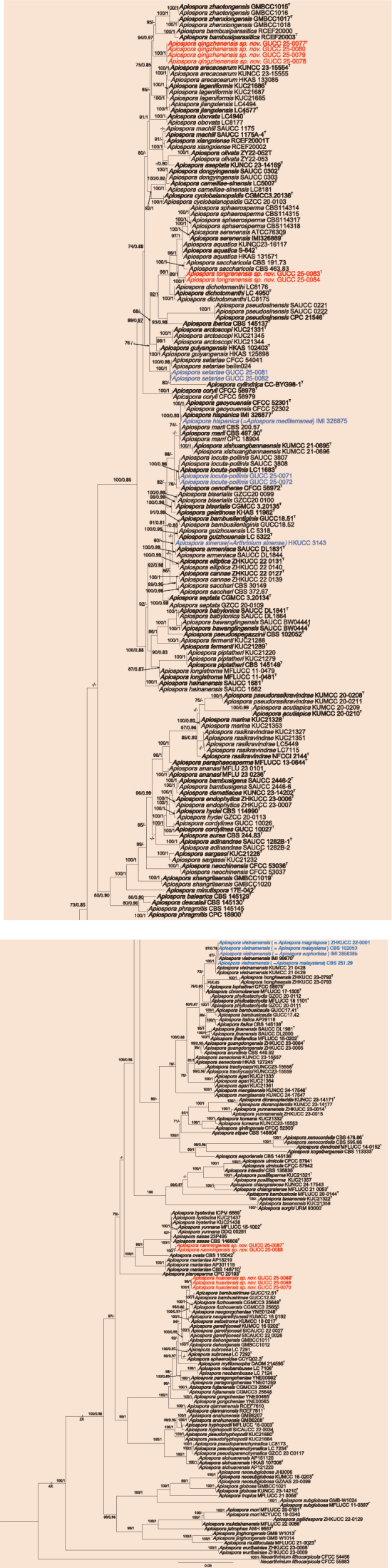
Phylogenetic tree constructed using Maximum likelihood (ML) analyses based on the multilocus alignment (ITS, LSU, *tef*1-α, and *tub2*) for *Apiospora*. The outgroup is *Neoarthrinium lithocarpicola* (CFCC 54456 and CFCC 55883). Maximum likelihood bootstrap proportions ≥ 70% (left) and Bayesian inference posterior probability ≥ 0.8 (right) are indicated at nodes (ML/BI). The type strains are followed by “^T^” and bold. New species in this study are red, and new records and merged species are blue.

The two phylogenetic trees of *Apiospora* and *Nigrospora* were conducted separately for each genus to more precisely determine their interrelationships. For *Apiospora* (Fig. 2), a concatenated alignment comprising ITS (1–466), LSU (467–1220), *tef1-α* (1221–1935), and *tub2* (1936–2303) loci was analyzed, including 282 strains with *Ne. lithocarpicola* (CFCC 54456 and CFCC 55883) designated as outgroup taxa. The ML topology served as the reference, with Bayesian posterior probabilities mapped onto corresponding branches. Both topologies were identical, and the best-scoring RAxML tree with a final ML optimization likelihood value of – 29198.663 is presented. Estimated base frequencies were as follows: A = 0.237, C = 0.250, G = 0.254, T = 0.259; substitution rates AC = 1.29435, AG = 3.16215, AT = 1.03614, CG = 0.85141, CT = 4.90526, GT = 1.0. Sequences from 15 isolates obtained in this study were incorporated into the dataset, forming six well-supported lineages within *Apiospora*, representing four new species (*A. huaxiensis* sp. nov., *A. nanmingensis* sp. nov., *A. qingzhenensis* sp. nov., and *A. tongrenensis* sp. nov.) and two known species (*A. locuta-pollinis* and *A. setariae*). *Apiospora huaxiensis* (GUCC 25-0068, GUCC 25-0069, and GUCC 25-0070) formed a sister clade with *A. bambusirimae* (GUCC12.51 and GUCC12.52) with high support (ML/BI = 100/1). *Apiospora nanmingensis* (GUCC 25-0087 and GUCC 25-0088), formed a sister clade with *A. hysterina* and *A. sasae*, with high support (ML/BI = 100/1). *Apiospora qingzhenensis* (GUCC 25-0077, GUCC 25-0078, GUCC 25-0079, GUCC 25-0080), formed a single clade with high support (ML/BI = 100/1). *Apiospora tongrenensis* (GUCC 25-0083 and GUCC 25-0084) formed a sister clade with *A. saccharicola*, with high support(ML/BI = 99/1). *Apiospora locuta-pollinis* and *A. setariae* each clustered separately from other strains of the species with high support values (ML/BI = 100/0.96 and ML/BI = 100/1). *Arthrinium sinense* (HKUCC 3143) clusters within the clade of *A. bambusilentiginis*, *A. guizhouensis*, *A. armeniaca*, *A. elliptica*, *A. cannae*, and *A. sacchari* with high support (ML/BI = 86/-). *Apiospora hispanica* (IMI 326877) and *A. mediterranea* (IMI 326875) clustered together on the phylogenetic tree with no genetic distance with a high support value (ML/BI = 100/0.93), which is consistent with the previous studies in Tian et al. (2021), Monkai et al. (2022), and Liao et al. (2023). *Apiospora vietnamensis* (IMI 99670, KUMCC 21-0428, KUMCC 21-0429), *A. malaysiana* (CBS 102053, CBS 251.29), *A. magnispora* (ZHKUCC 22-0001), and *A. euphorbiae* (IMI 285638b) clustered together with minimal genetic distance with a high support value (ML/BI = 100/1).

**Figure 3.**
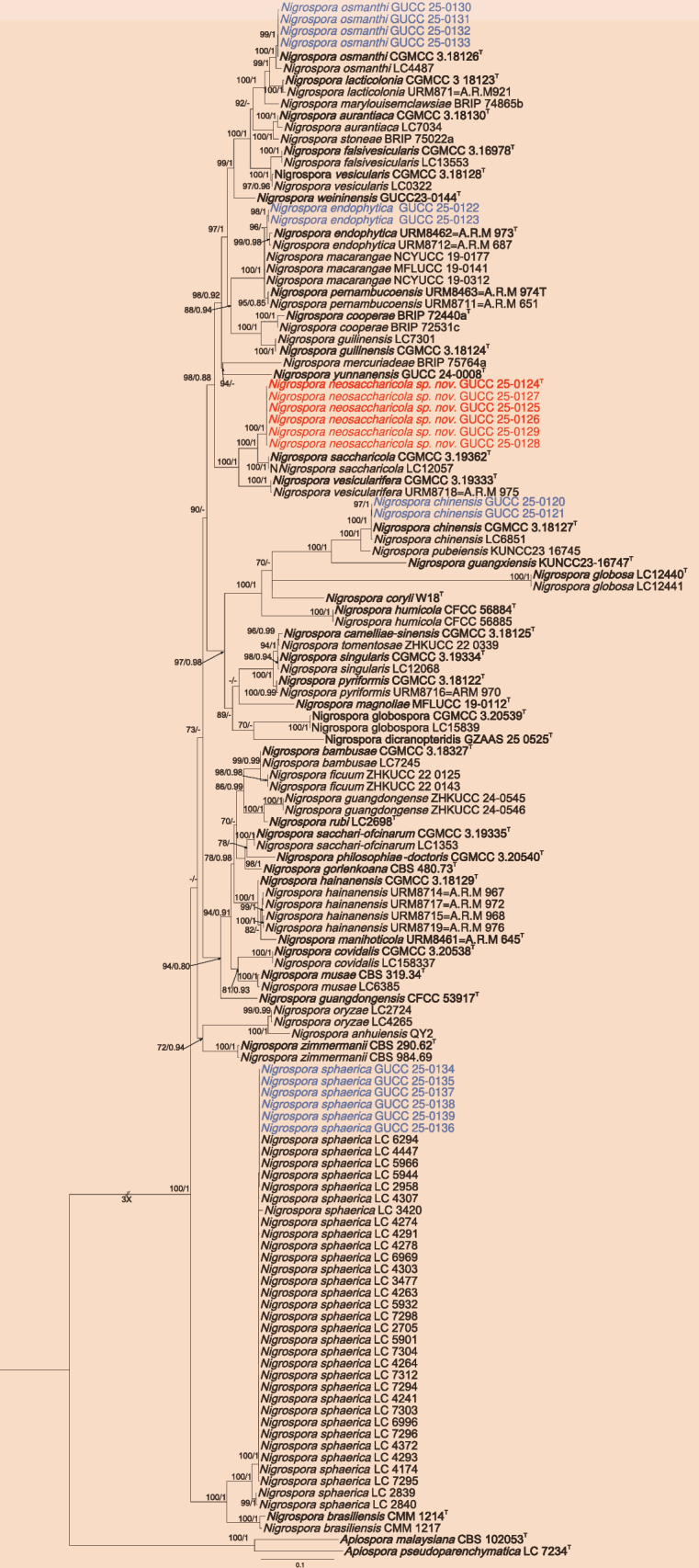
Phylogenetic tree constructed using Maximum likelihood (ML) analyses based on the multilocus alignment (ITS, *tef*1-α, and *tub2*) for *Nigrospora*. The outgroups are *A. pseudoparenchymatica* (LC 7234^T^) and *A. malaysiana* (CBS 102053). Maximum likelihood bootstrap proportions ≥ 70% (left) and Bayesian inference posterior probability ≥ 0.8 (right) are indicated at nodes (ML/BI). The type strains are followed by “^T^” and bold. New species discovered in this study are red, and new records are blue.

For *Nigrospora* (Fig. 3), phylogenetic relationships were inferred from a combined dataset of ITS (1–522), *tef1-α* (523–961), and *tub2* (962–1752) loci, comprising 132 strains with *A. pseudoparenchymatica* (LC 7234) and *A. malaysiana* (CBS 102053) as outgroup taxa. The ML topology was used as the reference, with Bayesian posterior probabilities mapped onto corresponding branches, and the best-scoring RAxML tree with a final ML optimization likelihood value of – 17739.687 is presented. Estimated base frequencies were as follows: A = 0.215, C = 0.306, G = 0.243, T = 0.237; substitution rates AC = 1.0, AG = 3.25843, AT = 1.0, CG = 1.0, CT = 4.42079, GT = 1.0. Twenty newly obtained isolates clustered into five well-supported lineages, representing one novel species (*N. neosaccharicola* sp. nov.) and four known species (*N. chinensis*, *N. endophytica*, *N. osmanthi*, and *N. sphaerica*). *Nigrospora neosaccharicola* (GUCC 25-0124, GUCC 25-0125, GUCC 25-0126, GUCC 25-0127, GUCC 25-0128, and GUCC 25-0129) formed a sister clade with *N. saccharicola* (CGMCC 3.19362 and LC 4241) with a high support value (ML/BI = 100/1). GUCC 25-0130, GUCC 25-0131, GUCC 25-0132, and GUCC 25-0133 clustered to the *N. osmanthi* clade with high support values (ML/BI = 100/1). GUCC 25-0122 and GUCC 25-0123 clustered to *N. endophytica* with high support values (ML/BI = 96/-). GUCC 25-0120 and GUCC 25-0121 clustered to *N. chinensis* with high support values (ML/BI = 100/1). GUCC 25-0134, GUCC 25-0135, GUCC 25-0136, GUCC 25-0137, GUCC 25-0138, and GUCC 25-0139 clustered to *N. sphaerica* with high support values (ML/BI = 100/1).

### Host and geographical distribution of *Apiospora* and *Nigrospora*

**Figure 4.**
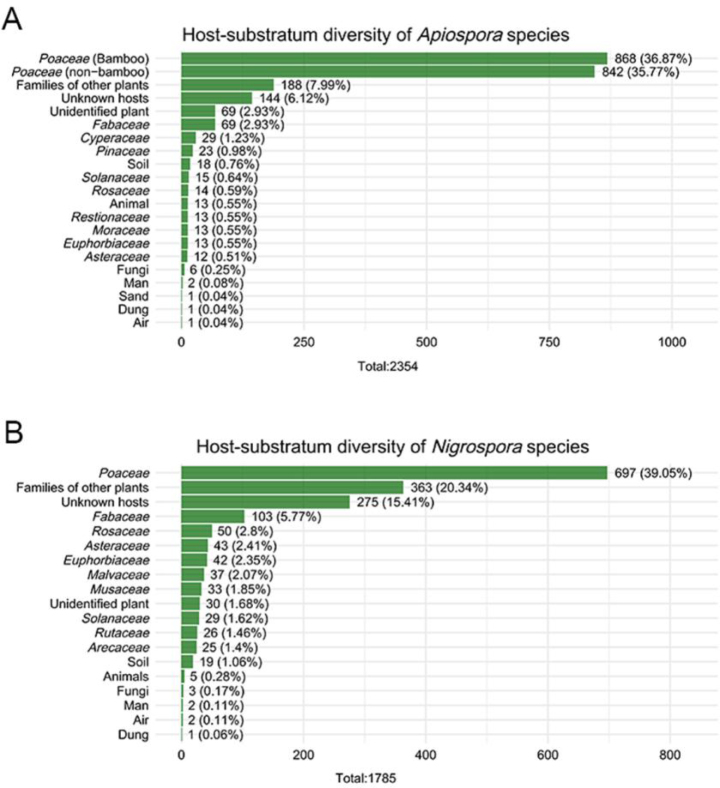
Host-substratum diversity of *Apiospora* (**A**) and *Nigrospora* (**B**) species according to GBIF.

**Figure 5.**
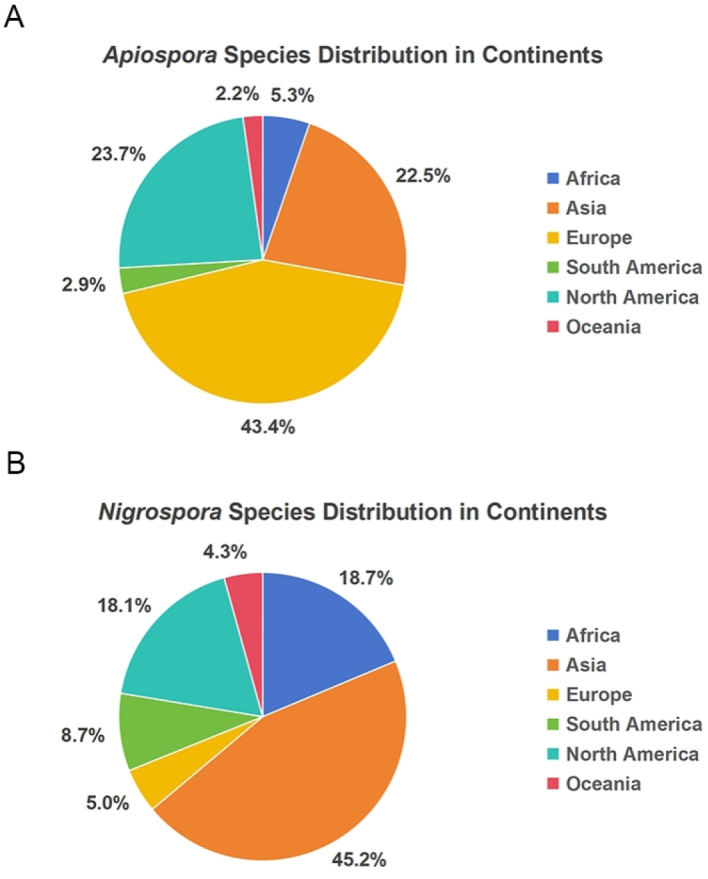
*Apiospora* (**A**) and *Nigrospora* (**B**) species distribution by continent according to GBIF.

**Figure 6.**
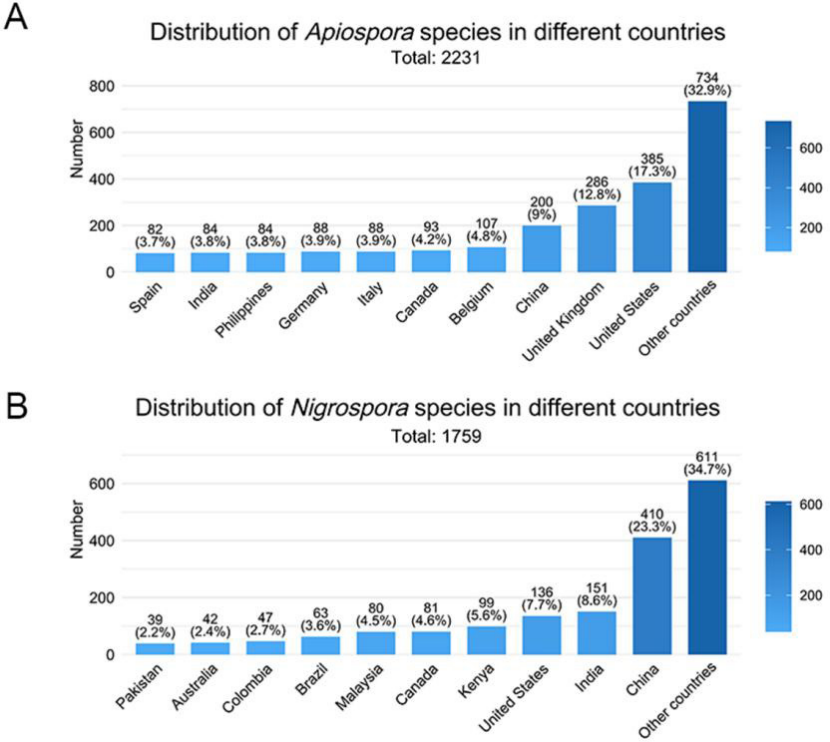
Distribution of *Apiospora* (**A**) and *Nigrospora* (**B**) species by countries according to GBIF.

According to GBIF data, based on host association, *Poaceae* represents the dominant host family for both genera (Fig. 4). For *Apiospora*, 1,710 records (72.64% of all host-associated records) were linked to *Poaceae*, with bamboo accounting for the largest proportion (868 records, 36.87% of all host-associated records). Additional host families with notable representation include *Fabaceae*, *Cyperaceae*, and *Pinaceae*. In contrast, 42 records were associated with non-plant substrates or atypical hosts, such as soil, animals, humans, and other fungi. Similarly, *Nigrospora* species were predominantly associated with *Poaceae*, comprising 697 records (39.05% of host-associated records). Additional host families with notable representation include *Fabaceae*, *Rosaceae*, and *Asteraceae* (Fig. 4). Regarding geographical distribution, *Apiospora* species are most frequently recorded in Europe, followed by Asia and North America (Fig. 5). In contrast, *Nigrospora* species are most widely reported from Asia, with Africa representing the second most recorded region (Fig. 5). At the country level, the highest numbers of *Apiospora* records originate from the USA, UK, and China (Fig. 6). For *Nigrospora*, the top three countries are China, India, and the USA (Fig. 6).

### Taxonomy

#### *Apiosporaceae* K.D. Hyde, et al. 1998.

***Apiospora* Sacc., Atti Soc. Veneto-Trent. Sci. Nat., Padova, Sér. 4 4: 85. 1875**.

##### Apiospora hispanica

Taxon classificationAnimaliaXylarialesApiosporaceae

(Larrondo & Calvo) Pintos & P. Alvarado, Fungal Systematics and Evolution 7: 205. 2021.

B8C1FFC2-09C4-5AAD-9B92-CFAD670D68F8

837674

###### Basionym.

*Arthrinium hispanicum* Larrondo & Calvo, Mycologia 84(3): 476. 1992.

###### Synonyms.

*Arthrinium mediterranei* Larrondo & Calvo, Mycologia 84(3): 476. 1992.

*Apiospora mediterranea* (Larrondo & Calvo) Pintos & P. Alvarado, Fungal Systematics and Evolution 7: 206. 2021. syn. nov.

###### Substrate and distribution.

Beach sand and air, Spain (Larrondo and Calvo 1992).

###### Notes.

*Arthrinium hispanicum* was originally described by Larrondo and Calvo (1992) from maritime sand in Spain, while *Ar. mediterranei* was isolated from the air in the same region (Larrondo and Calvo 1992). The two taxa were morphologically distinguished primarily by conidial dimensions (7.5–8.5 × 6.2–7.6 µm vs. 9–9.5 × 7.5–9 µm, respectively). Pintos and Alvarado (2021) later clarified the relationship between *Apiospora* and *Arthrinium*, transferring both *Ar. hispanicum* and *Ar. mediterranei* to *Apiospora* based on multilocus phylogenetic analyses. However, they did not explicitly evaluate whether the two taxa represent a single species. Our phylogenetic analyses reveal that strains of *A. hispanica* (IMI 326877) and *A. mediterranea* (IMI 326875) form a single clade without genetic distinction. Comparative analyses of ITS, LSU, and *tub2* sequences showed no nucleotide differences between the two species. Based on the congruence of morphological features and the molecular divergence, we accept *A. hispanica* as the correct name and regard *A. mediterranea* syn. nov. as its synonym.

##### Apiospora huaxiensis

Taxon classificationAnimaliaXylarialesApiosporaceae

J. E. Sun, X. C. Wang, K.D. Hyde & Yong Wang bis sp. nov.

1BB615FD-02B8-5B35-8A55-B8EBD48E2250

860108

Fig. 7

###### Etymology.

The species name refers to the collected location, Huaxi District, Guizhou Province.

**Figure 7.**
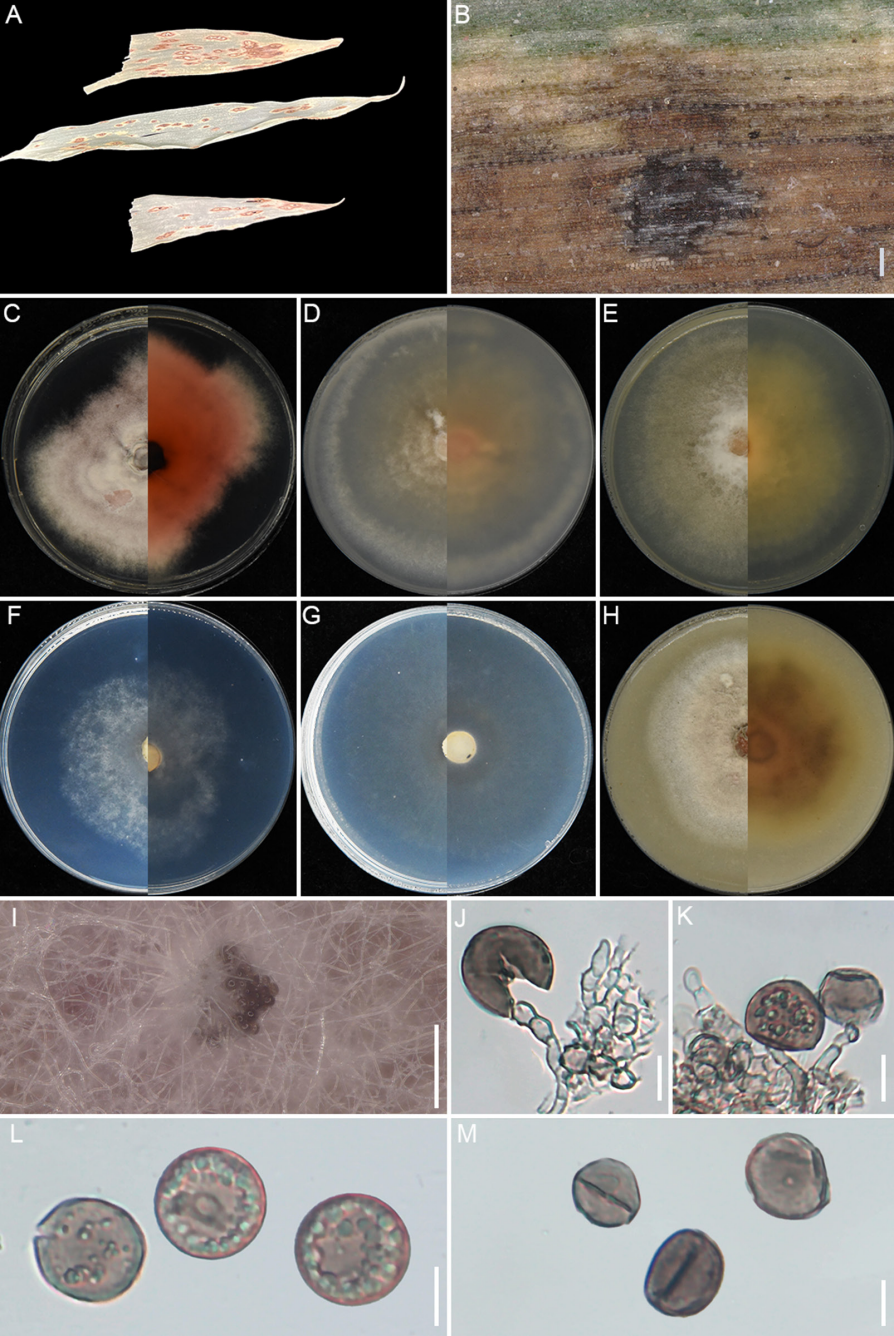
*Apiospora huaxiensis* (GUCC 25-0068). **A, B** Appearance of the fungus on spot leaves of bamboo; **C–H** Upper view and reverse view of culture on PDA, MEA, CMA, SNA, WA, and OA after 7 days. (I) Close up of PDA culture with conidia; **J, K** Conidia with conidiogenous cells. (L) Conidia. (M) Conidia with germ slit. Scale bars: 100 μm (**B**); 10 μm (**J–M**).

###### Holotype.

China • Guizhou Province, Guiyang City, Huaxi District, Huaxi Park, on the leaf spots of bamboo, 23 November 2023. X.C. Wang, HGUP 25-0006 (holotype); ex-type GUCC 25-0068.

###### Description.

Associated with the leaf spots of bamboo. Lesions are black and brown spots with yellow halos on leaves, subglobose and ellipsoidal in shape, measuring 250–700 μm in diam. **Sexual morph**: Not observed. **Asexual morph**: on PDA, ***Hyphae*** 2.4–4.1 μm wide, branched, septate, hyaline to pale green. ***Conidiophores*** reduced to conidiogenous cells. ***Conidiogenous cells*** 3.6–6.5 × 2.3–5.8 μm (x̄ =5.2 × 3.2 μm, n=30), cylindrical or round, monoblastic, pale green. ***Conidia*** 13.8–17.6 × 11.3–16.4 μm (x̄ =15.8 × 13.7 μm, n=30), globose to ellipsoidal granules, pale green to dark brown, with longitudinal germ-slit.

###### Culture characteristics.

After 7 days at 25°C, on PDA, colonies reach 70–74 mm in diam., with irregular margins, flat, cottony, dense aerial mycelia, pale red coloration, and a red reverse; they produce scarlet pigment. On MEA, colonies reach 88–90 mm diam., with regular margins, flat, dense, floccose, surface pale salmon and reverse salmon. On CMA, colonies reach 85–87 mm diam., cottony, flat, dense, irregular margins with aerial mycelia, surface straw, and reverse pale luteous. On SNA, colonies reach 55–59 mm diam., flat, spreading, with sparse aerial mycelia and regular margin, surface and reverse white. On WA, colonies reach 22–26 mm diam., flat, aerial mycelia scant, surface and reverse white. On OA, colonies reach 72–74 mm diam., flat, cottony, with regular floccose margin, surface pale ochreous and reverse umber middle area and pale-yellow outer area. Sporulation was abundant only on PDA after 14 days.

###### Material examined.

China • Guizhou Province, Guiyang City, Huaxi District, Huaxi Park, on the leaf spots of bamboo, 23 November 2023, X.C. Wang, HGUP 25-0006 (holotype); GUCC 25-0068 (ex-type), GUCC 25-0069 and GUCC 25-0070.

###### Notes.

*Apiospora huaxiensis* (GUCC 25-0068, GUCC 25-0069, and GUCC 25-0070) formed a sister clade to *A. bambusirimae* (GUCC 12.51) with strong statistical support (100% ML/1.0 BI) (Fig. 2). Morphologically, the conidia of *A. huaxiensis* are smaller than those of *A. bambusirimae* (x̄ = 15.8 × 13.7 μm vs. x̄ = 20.5 × 13 μm; Yu et al. 2025). In culture, *A. huaxiensis* produced reddish pigment on PDA after 3 days, whereas *A. bambusirimae* did not on the same cultivation conditions (Yu et al. 2025). Moreover, pairwise sequence comparisons between the two species revealed nucleotide differences of 2.34% in ITS (11/469 bp, including six gaps) and 2.65% in *tef1-α* (12/453 bp, including nine gaps).

##### Apiospora locuta-pollinis

Taxon classificationAnimaliaXylarialesApiosporaceae

(F. Liu & L. Cai) Pintos & P. Alvarado, Fungal Systematics and Evolution 7: 206. 2021.

0A314D1F-8365-5336-894A-8283350FD193

837763

Fig. 8

###### Basionym.

*Arthrinium locutum-pollinis* F. Liu & L. Cai [as ‘locuta-pollinis’], in Zhao, Zhang et al., Mycosphere 9(6): 1094. 2018.

**Figure 8.**
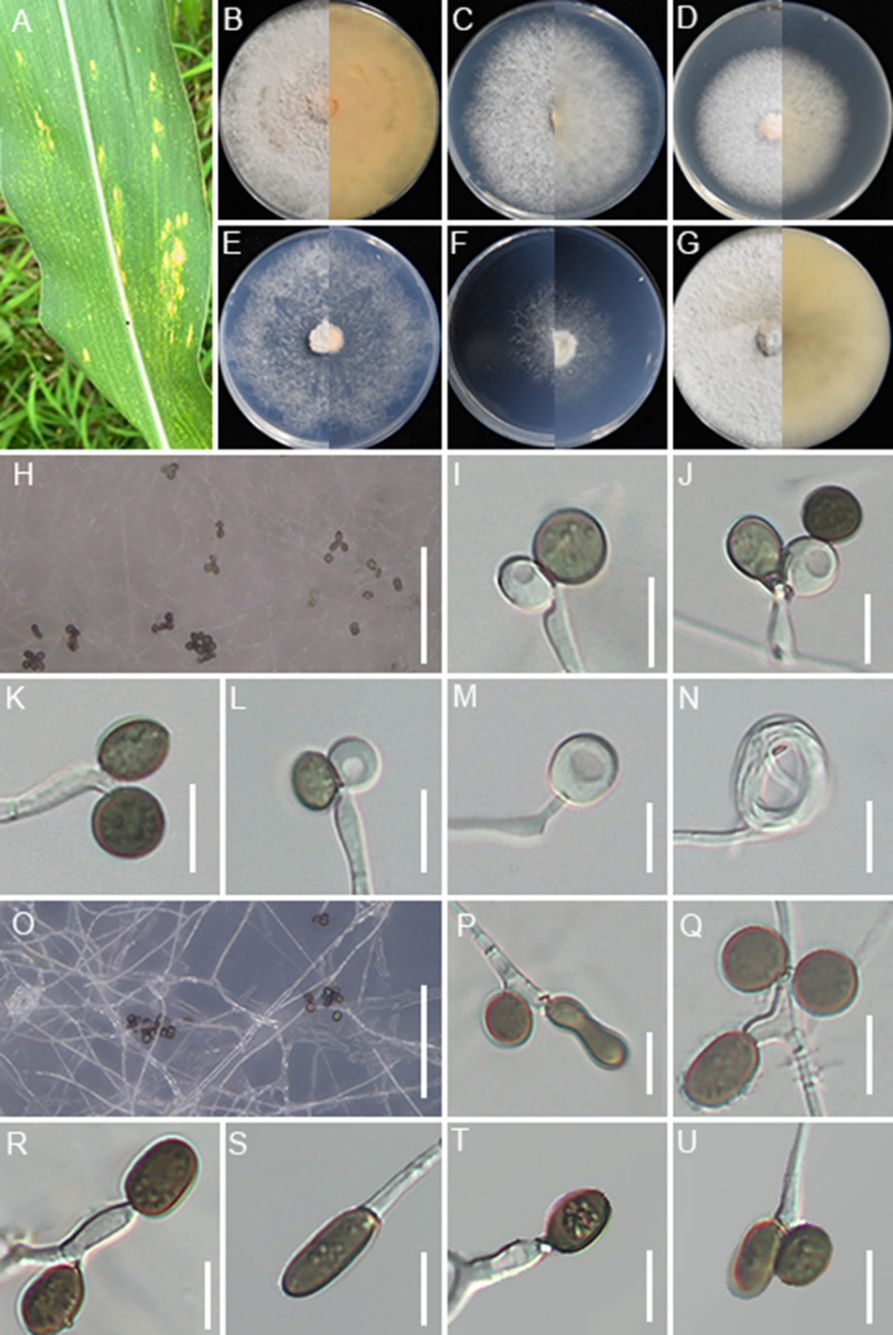
*Apiospora locuta-pollinis* (GUCC 25-0071). **A** Appearance of the fungus on leaves of maize; **B–G** Upper view and reverse view of culture on PDA, MEA, CMA, SNA, WA, and OA after 7 days; **H, O** Close up of WA; **H** and SNA; **O** cultures with conidia; **I–M** Conidia with conidiogenous cells on WA; **N** coiled hyphae on WA; **P–U** Conidia with conidiogenous cells on SNA. Scale bars: 100 μm (**H, O**); 10 μm (**I–N, P–U**).

###### Synonym.

*Arthrinium pseudomarii* T.Z. Chen, Yong Wang bis & K.D. Hyde, in Chen, Zhang et al., Mycotaxon 136(1): 191. 2021.

###### Substrate and distribution.

On hive-stored pollen, Hubei Province, China (Zhao et al. 2018); *Aristolochia debilis*, China (Chen et al. 2021); Bamboo, China (Monkai et al. 2022); *Musa* sp., Thailand (Samarakoon et al. 2024); grass, China (Gao et al. 2025).

###### Description.

Associated with the leaf spots of maize. Lesions are yellow spots with pale brown halos on leaves, subglobose and spindle-shaped in shape, measuring 300–1000 μm in diam. **Sexual morph**: Not observed. **Asexual morph**: on WA, ***Hyphae*** 2.1–4.1 μm wide, occasionally coiled, septate, branched hyphae, hyaline. ***Conidiophores*** reduced to conidiogenous cells. ***Conidiogenous cells*** 3.9–5.1 × 1.9–3.2 μm (x̄ = 4.2 × 2.1 μm, n = 30), subglobose to ampulliform, mostly polyblastic, hyaline to pale green. ***Conidia*** 4.2–10.1 × 3.9–8.7 μm (x̄ = 7.7 × 5.9 μm, n = 30), globose to subglobose, smooth-walled, pale green when immature, brown when mature. On SNA, ***Conidiophores*** reduced to conidiogenous cells. ***Conidiogenous cells*** 3.5–8.2 × 2.1–4.2 μm (x̄ = 4.9× 2.7 μm, n = 30), subglobose to ampulliform, mostly polyblastic, pale green. ***Conidia*** 4.8–13.9 × 3.7–7.6 μm (x̄ = 8.2× 6.2 μm, n = 30), globose, subcylindrical to ovate, smooth, brown.

###### Culture characteristics.

After 7 days at 25°C, on PDA, colonies reach 88–90 mm in diam., flat, cottony, with regular margins; surface pale straw, reverse salmon. On MEA, colonies reach 70–74 mm diam., flat, with regular margins, surface, and reverse white. On CMA, colonies reaching 56–58 mm in diam., flat, with regular margins, surface, and reverse white. On SNA, colonies reach 77–79 mm in diam., flat, spreading, with scant aerial mycelia, and a regular margin, surface, and reverse that are white. On WA, colonies reach 42–45 mm diam., flat, spreading, with irregular margins, aerial mycelia scant, surface and reverse white. On OA, colonies reach 81–84 mm diam., circular, flat, cottony, with regular margin, surface white and reverse pale saffron. Sporulation was abundant on WA and SNA after 14 days.

###### Material examined.

• Guizhou Province, Liupanshui City, leaf spot on *Zea mays*, 16 September 2024, X.C. Wang, HGUP 25-0058; GUCC 25-0071; • ibid. GUCC 25-0072.

###### Notes.

GUCC 25-0071 and GUCC 25-0072 clustered with *A. locuta-pollinis* (LC 11683). Morphologically, the new collections also closely resemble *A. locuta-pollinis* (LC 11683), with globose to subglobose conidia (x̄ = 7.7 × 5.9 μm vs. x̄ = 7.1 × 6.4 μm) and subglobose to ampulliform conidiogenous cells (x̄ = 4.2 × 2.1 μm vs. x̄ = 4.9 × 3.8 μm) (Zhao et al. 2018). Nucleotide comparison with LC 11683 revealed that only *tef1-α* exhibited two nucleotide differences (2/430). Therefore, strains GUCC 25-0071 and GUCC 25-0072 are identified as *A. locuta-pollinis*. This is the first report of *A. locuta-pollinis* isolated from maize.

##### Apiospora nanmingensis

Taxon classificationAnimaliaXylarialesApiosporaceae

X. C. Wang, S. Q. Guo, K.D. Hyde & Yong Wang bis sp. nov.

E3288603-4944-526E-8BF5-08EDF78B43F7

861263

Fig. 9

###### Etymology.

This species was named according to the location where the fungus was first collected, Nanming District, Guiyang City, Guizhou Province.

**Figure 9.**
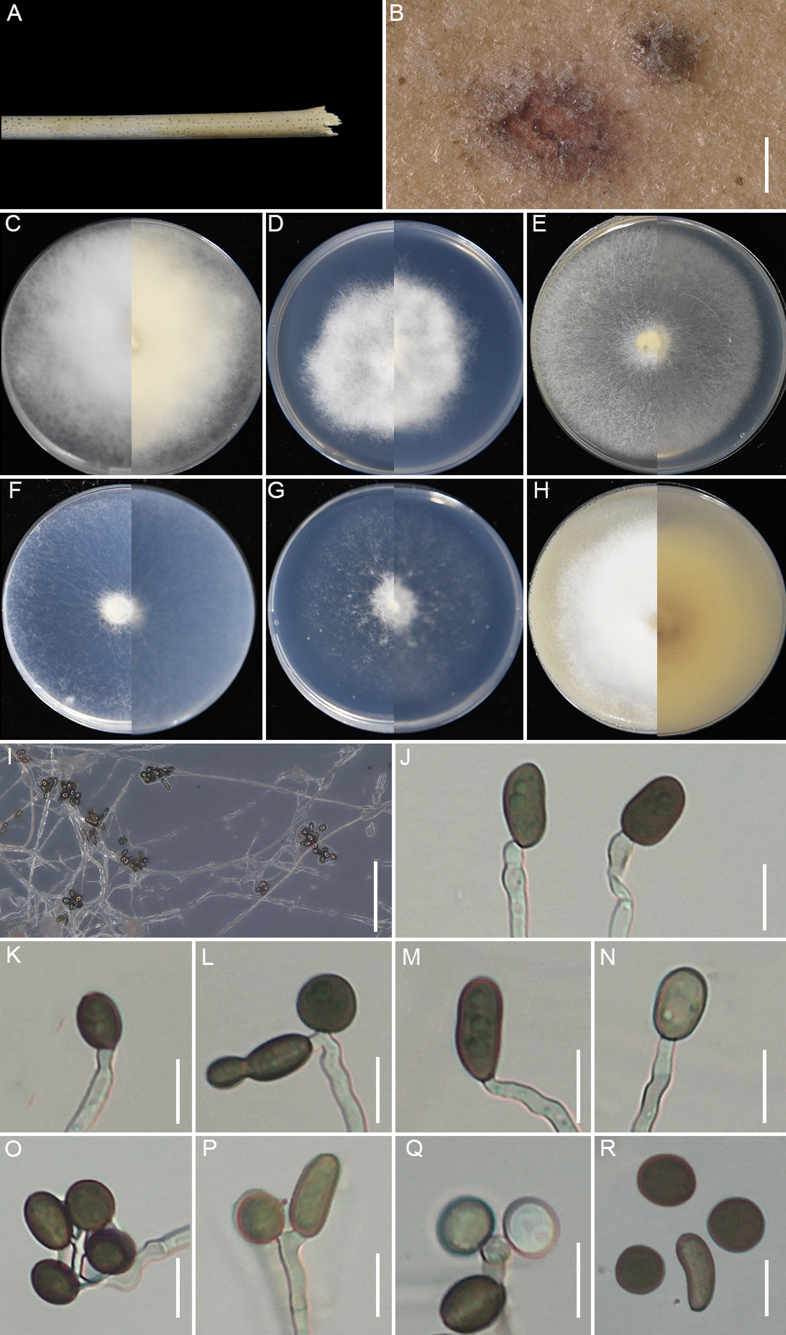
*Apiospora nanmingensis* (GUCC 25-0087). **A** Appearance of the fungus on stem of bamboo; **B** Conidiomata on host; **C–H** Upper view and reverse view of culture on PDA, MEA, CMA, SNA, WA, and OA after 7 days; **I** Close up of SNA culture with conidia; **J–Q** Conidia with conidiogenous cells on SNA; **R** Conidia. Scale bars: 100 μm (**B, I**); 10 μm (**J–R**).

###### Holotype.

China • Guizhou Province, Guiyang City, Nanming District, Guiyang Forest Park, on the stem of bamboo, 1 April 2024. X.C. Wang, HGUP 25-0053 (holotype); ex-type GUCC 25-0087.

###### Description.

Isolated from decaying stems of bamboo. On the host, ***Conidiomata*** punctiform, brown, 150–400 µm × 125–200 µm (n = 30). ***Conidia*** 9.2 × 15.2–7.4 × 13.2 μm (x̄ = 13.2 × 11.4 μm, n=30), globose to subglobose, brown. **Sexual morph**: Not observed. **Asexual morph**: on SNA, ***Hyphae***1.5–3.8 µm wide, branched, septate, smooth, hyaline. ***Conidiophores*** reduced to conidiogenous cells. ***Conidiogenous cells*** 3.9–11.3 × 2.5–3.9 μm (x̄ = 6.8 × 3.1 μm, n=30), cylindrical to globose, monoblastic to mostly polyblastic, aggregated, hyaline to pale green. ***Conidia*** 7.6–10.4 × 6.9–10.1 μm (x̄ = 8.9 × 7.5 μm, n=30), globose, subglobose to lenticular, with longitudinal germ slit, pale green to brown.

###### Culture characteristics.

Colonies for 7 days at 25°C: On PDA, colonies reach 88–90 mm diam., flat, spreading, with irregular margins, dense aerial mycelia, surface white and reverse pale salmon center and white margin. On MEA, colonies reach 57–60 mm diam., flat, floccose, cottony, with regular margins, surface and reverse white. On CMA, colonies reach 77–80 mm diam., flat, with regular margins, spreading, with moderate aerial mycelia, surface and reverse pale salmon. On SNA, colonies reach 87–90 mm diam., flat, aerial mycelia scant, with regular margins, surface and reverse white. On WA, colonies reach 63–65 mm diam., flat, aerial mycelia scant, with regular margins, surface and reverse white. On OA, colonies reach 70–72 mm diam., cottony, with dense aerial mycelia, regular margins, surface white and reverse pale straw. Sporulation was abundant only on SNA after 14 days.

###### Material examined.

China • Guizhou Province, Guiyang City, Nanming District, Guiyang Forest Park, on the stem of bamboo, 1 April 2024. X.C. Wang, HGUP 25-0053 (holotype); GUCC 25-0087 (ex-type), GUCC 25-0088.

###### Notes.

Based on the phylogenetic analyses, the two strains obtained in this study, GUCC 25-0087 and GUCC 25-0088, formed a distinct, well-supported lineage (100% ML/1.0 BI) with *A. hysterina*, *A. sasae*, and *A. yunnana* (Fig. 2). Morphologically, our strains can be readily distinguished from these closely related taxa by their smaller conidia, which measure 7.6–10.4 × 6.9–10.1 µm (vs. 15–18 × 14–16.5 µm in *A. hysterina*; Kwon et al. 2022; Saccardo 1893;17–18 × 16–17 µm in *A. sasae*; Crous et al. 2021; and 17.5–26.5 × 15.5–25 µm in *A. yunnana*; Dai et al. 2017). The conidiogenous cells are likewise smaller than those of *A. yunnana* (3.9–11.3 × 2.5–3.9 µm vs. 16.5–50 × 2–4 µm). Interestingly, in this clade, the shape and size of the conidia of *A. hysterina*, *A. sasae*, and *A. yunnana* are relatively similar, whereas the conidia of *A. nanmingensis* that we identified differ significantly in shape and size from those of the other species, whether observed on the host or on culture media. *Apiospora* species are variable in morphological characteristics depending on growth conditions, and molecular and phylogenetic analysis are the key to recognize species in this genus. Pairwise sequence comparisons revealed that strains GUCC 25-0087 and GUCC 25-0088 differ from *A. hysterina* (ICPM 6889) by 2.72% in ITS (14/441 bp, including two gaps), 1.86% in *tef1-α* (8/430 bp, four gaps), and 1.19% in *tub2* (9/758 bp, one gap). Comparisons with *A. sasae* (CBS 146808) showed divergences of 2.02% in ITS (10/496 bp, three gaps) and 1.55% in *tub2* (12/773 bp). Comparisons with *A. yunnana* (MFLUCC 15-1002) showed divergences of 4.77% in ITS (26/545 bp, seven gaps) and 0.34% in LSU (3/875 bp, one gap).

##### Apiospora qingzhenensis

Taxon classificationAnimaliaXylarialesApiosporaceae

X.C. Wang, K.D. Hyde & Yong Wang bis sp. nov.

5122DDF4-296D-5F00-971D-5CA83CCB81FC

860112

Fig. 10

###### Etymology.

The name refers to Qingzhen City in Guizhou Province, where the fungus was collected.

**Figure 10.**
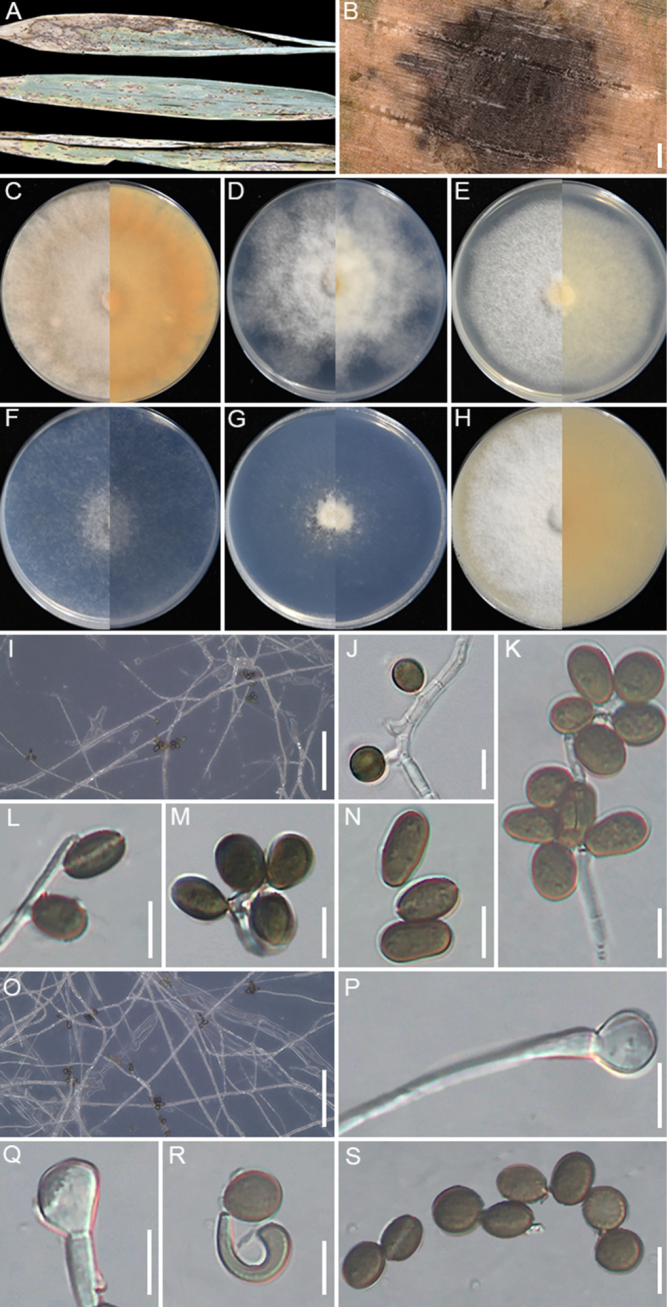
*Apiospora qingzhenensis* (GUCC 25-0077). **A, B** Appearance of the fungus on leaf spots of bamboo; **C–H** Upper view and reverse view of culture on PDA, MEA, CMA, SNA, WA, and OA after 7 days; **I, O** Close up of WA; **I** and SNA; **O** cultures with conidia; **J–M** Conidia with conidiogenous cells on WA; **N** Conidia on WA; **P, Q** Conidia with conidiogenous cells on SNA; **R** Conidia and sterile cell on SNA; **S** Conidia on SNA. Scale bars: 100 μm (**B, I, O**); 10 μm (**J–N, P–S**).

###### Holotype.

China • Guizhou Province, Qingzhen City, on the leaves of bamboo with spots, 18 January 2024, X.C. Wang, HGUP 25-0008 (holotype); ex-type GUCC 25-0077.

###### Description.

Associated with bamboo leaf spots. Lesions are black spots on leaves, subglobose in shape, measuring 250–620 μm in diam. **Sexual morph**: Not observed. **Asexual morph**: On WA, ***Hyphae*** 2.1–5.9 μm in diam, branched, septate, hyaline. ***Conidiophores*** reduced to conidiogenous cells. ***Conidiogenous cells*** 4.1–6.3 × 2.1–5.7 μm (x̄ = 5.7 × 3.6 μm, n=30), cylindrical, mostly polyblastic, aggregated, hyaline or pale green. ***Conidia*** 7.1–17.5 × 6.0–11.4 μm (x̄ = 10.7 × 8.7 μm, n=30), globose, subglobose to ovate with a straight germ-slit along spore length, green to dark brown. On SNA, ***Conidiogenous cells*** 5.2–8.3 × 2.7–6.1 μm (x̄ = 6.1 × 3.9 μm, n=30), cylindrical, mostly polyblastic, aggregated, hyaline or pale green. ***Conidia*** 6.5–14.3 × 6.0–12.4 μm (x̄ = 11.8 × 9.3 μm, n=30), globose to subglobose, pale green for immature and dark brown for mature. Sterile cells extremely rare, pale brown, rolled up, irregularly lobed.

###### Culture characteristics.

Colonies for 7 days at 25°C: On PDA, colonies reach 90 mm diam., flat, spreading, cottony, dense aerial mycelia, with regular margins, surface, and reverse pale flesh. On MEA, colonies reach 77–80 mm diam., flat, with moderate aerial mycelia, irregular margins, surface white, and reverse center pale salmon, with margin white. On CMA, colonies reach mm 71–74 diam., flat, spreading, with moderate aerial mycelia, regular margins, surface and reverse pale salmon. On SNA, colonies reach 90 mm diam., flat, aerial mycelia scant, with regular margins, surface and reverse pale white. On WA, colonies reach 53–57 mm diam., flat, aerial mycelia scant, regular margins filamentous, surface and reverse white. On OA, colonies reach 81–84 mm diam., floccose, flat, spreading, with dense aerial mycelia, regular margins, surface white and reverse pale salmon. Sporulation was abundant on WA and SNA after 14 days.

###### Material examined.

China • Guizhou Province, Qingzhen City, on the leaves of bamboo with spots, 18 January 2024, X.C. Wang, HGUP 25-0008 (holotype); GUCC 25-0077 (ex-type), GUCC 25-0078, GUCC 25-0079, and GUCC 25-0080.

###### Notes.

Based on phylogenetic analyses (Fig. 2), four strains (GUCC 25-0077, GUCC 25-0078, GUCC 25-0079, and GUCC 25-0080) formed a distinct and well-supported clade within *Apiosporasensu stricto*. The closest related species are *A. zhaotongensis*, *A. zhenxiongensis*, and *A. bambusiparasitica*. Since *A. zhaotongensis* and *A. zhenxiongensis* have been reported only from their sexual morphs (Han et al. 2024), morphological comparisons were made solely with *A. bambusiparasitica*. Morphologically, the new species differs from *A. bambusiparasitica* by having smaller conidiogenous cells (4.1–6.3 × 2.1–5.7 μm vs. 7–17 × 2.0–4.5 μm) and slightly larger conidia (x̄ = 10.7 × 8.7 μm vs. x̄ = 9.2 ± 0.9 × 8.1 ± 1.1 μm; Chang et al. 2025). Occasionally, pale brown, elongated sterile cells can be observed in our species. Furthermore, pairwise sequence comparisons across four gene regions revealed clear nucleotide differences between the new species and its closest relatives. The new strains differ from *A. zhaotongensis* (GMBCC 1015) by 4.34% in *tub2* (21/484 bp, including seven gaps) and 5.66% in *tef1-α* (25/442 bp, including five gaps); from *A. zhenxiongensis* (GMBCC 1017) by 5.15% in *tub2* (25/485 bp, including eight gaps) and 5.67% in *tef1-α* (25/441 bp, including five gaps); and from *A. bambusiparasitica* (RCEF 20003) by 4.72% in *tub2* (23/487 bp, including ten gaps) and 6.39% in *tef1-α* (28/438 bp, including six gaps).

##### Apiospora setariae

Taxon classificationAnimaliaXylarialesApiosporaceae

(C.M. Tian & N. Jiang) X.G. Tian & Tibpromma, Life 11(no. 1071): 19. 2021.

2046CA96-5EC6-59E2-AF86-9CF13455A6F2

558561

Fig. 11

###### Basionym.

*Arthrinium setariae* C.M. Tian & N. Jiang, in Jiang & Tian, Phytotaxa 484(2): 153. 2020.

**Figure 11.**
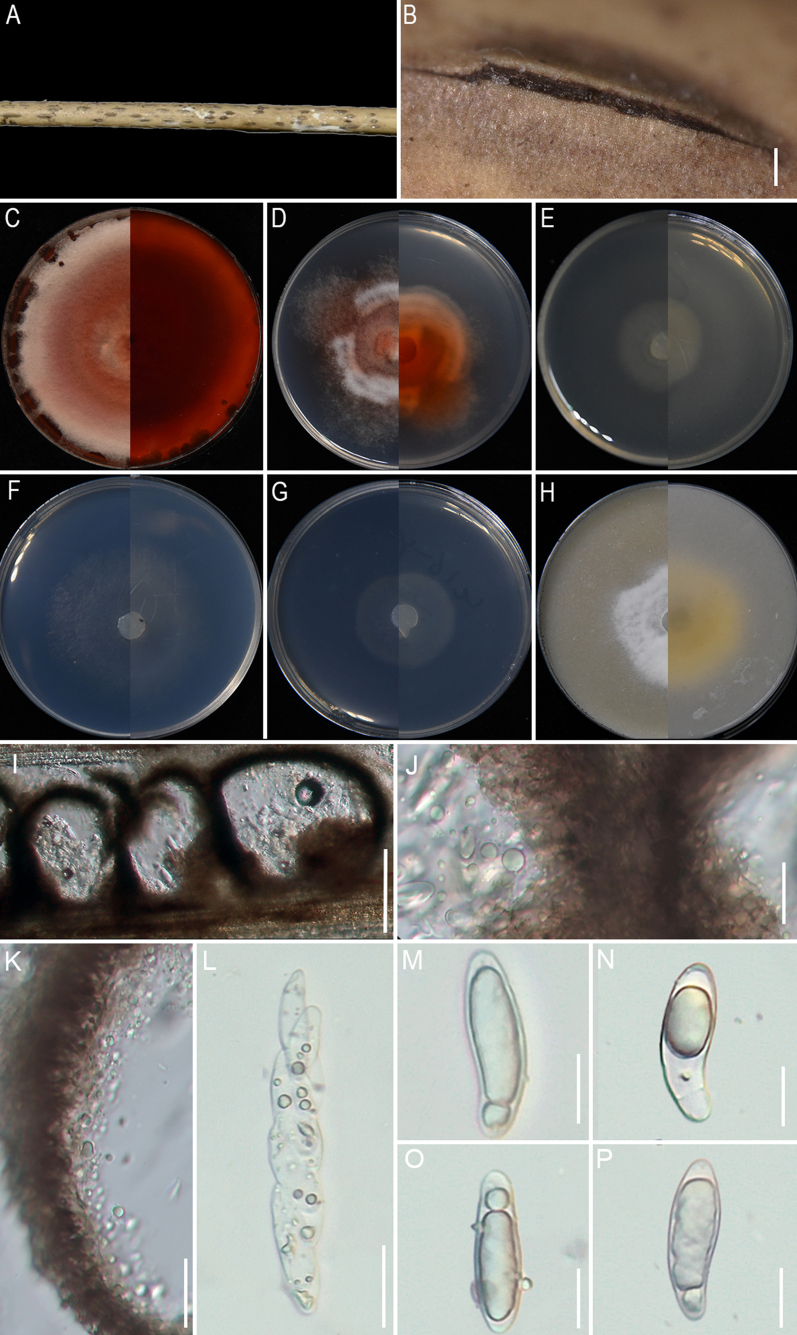
*Apiospora setariae* (GUCC 25-0081). **A** Appearance of the fungus on stem of bamboo stems; **B** Stromata on bamboo stems; **C–H** Upper view and reverse view of culture on PDA, MEA, CMA, SNA, WA, and OA after 7 days; **I** Transverse sections of the stromata; **J, K** Peridium; **L** Asci; **M–P** Ascospores. Scale bar: 100 μm (**B, I**); 20 μm (**J–L**); 10 μm (**M–P**).

###### Substrate and distribution.

*Setaria viridis*, China (Jiang and Tian 2021).

###### Description.

Associated with decaying stems of bamboo. **Sexual morph**: ***Stromata*** 400–1250 × 270–495 μm (n = 30), solitary to gregarious, partially immersed to erumpent, fusiform, raised on the host surface, with a slit-like opening, multi-loculate. ***Ascomata*** 70–240 μm (n = 30) diam., uniseriate or irregularly arranged beneath stromata, globose to subglobose. ***Peridium*** 5–20 μm (n = 30) thick, composed of 3–5 layers of brown to hyaline cells arranged in textura angularis. ***Hamathecium*** not observed. ***Asci*** 67.1–91.6 × 10.1–20.4 μm (x̄ = 79.9 × 15 μm, n = 30), 8-spored, broadly cylindrical, with an indistinct pedicel. ***Ascospores*** 22.9–29.4 × 6.9–10.9 μm (x̄ = 25.9 × 9 μm, n = 30), broadly ellipsoidal, composed of a large upper cell and a small lower cell, hyaline to pale green, smooth-walled. **Asexual morph**: refer to (Jiang and Tian 2021).

Culture characteristics—Colonies for 7 days at 25°C: On PDA, colonies reaching 88–90 mm diam., flat, spreading, dense aerial mycelia, with regular margins, surface and reverse scarlet, produce scarlet pigment. On MEA, colonies reach 55–58 mm diam., flat, spreading, with irregular margins, surface and reverse center scarlet and margin white, produce reddish pigment. On CMA, colonies reach 31–33 mm diam., flat, aerial mycelia scant, regular margins, surface and reverse white. On SNA, colonies reach 41–43 mm diam., flat, aerial mycelia scant, regular margins, surface and reverse white. On WA, colonies reach 30–32 mm diam., flat, aerial mycelia scant, regular margins, surface and reverse white. On OA, colonies reach 44–47 mm diam., flat, cottony, dense aerial mycelia, with regular margins, surface white and reverse salmon. Sporulation was absent on any medium after 14 days.

###### Material examined.

China • Guizhou Province, Guiyang City, Huaxi District, Huaxi Park, on the decaying bamboo stems, 17 April 2024, X.C. Wang, HGUP 25-0059; GUCC 25-0081 and GUCC 25-0082.

###### Notes.

In phylogenetic analyses, the newly generated strains in this study, GUCC 25-0081 and GUCC 25-0082, clustered with *A. setariae* (strains CFCC 54041 and beilin024) with ML/BI = 100/1 (Fig. 2). Morphologically, our new collections are very similar to *A. setariae* in the asci size (67.1–91.6 × 10.1–20.4 μm vs. 60–95 × 15–21 μm) and ascospores (22.9–29.4 × 6.9–10.9 μm vs. 18–23 × 8–11 μm, Jiang and Tian 2021). Therefore, both collections are identified as *A. setariae*. Previously, *A. setariae* was reported only from decaying culms of *Setaria viridis* (Jiang and Tian 2021). The comparison of nucleotide differences between strain GUCC 25-0081 and the type strain of *A. setariae* (CFCC 54041T) revealed clear sequence divergence: 0.69% in ITS (4/583 bp, including one gap), 0.41% in *tub2* (3/730 bp, including one gap), and 0.46% in *tef1-α* (2/439 bp, no gaps). In this study, the species was isolated from bamboo stems, representing a new host record for *A. setariae*.

##### Apiospora sinense

Taxon classificationAnimaliaXylarialesApiosporaceae

K.D. Hyde, J. Fröhl. & Joanne E. Taylor, Sydowia 50(1): 27. 1998 syn. nov.

12F85AAE-322C-5344-9B79-DFBFD131F025

816836

###### Synonym.

*Arthrinium sinense* (K.D. Hyde, J. Fröhl. & Joanne E. Taylor) Crous & J.Z. Groenew., in Réblová et al., IMA Fungus 7(1): 140. 2016.

###### Substrate and distribution.

On dead petiole of *Trachycarpus fortunei*, Hubei Province, China (Hyde 1998).

###### Morphological description.

refer to Hyde et al. (1998).

###### Notes.

*Apiospora sinensis* was described initially from dead petiole of *Trachycarpus fortunei* by Hyde et al. (1998). Due to uncertainty regarding the taxonomic boundaries between *Apiospora* and *Arthrinium*, Crous and Groenewald in 2016 subsequently transferred this species to *Arthrinium* based on phylogenetic analysis (Réblová et al. 2016). Pintos and Alvarado (2021) clarified the generic delimitation between *Apiospora* and *Arthrinium*, and reassigned most species previously placed in *Arthrinium* to *Apiospora*. Currently, only LSU sequence data (AY083831) is available for this species in GenBank. In our phylogenetic analyses, *Ar. sinensis* (HKUCC 3143) clustered within *Apiosporasensu stricto* and formed an independent branch distinct from other taxa. Compared to the reported morphology of this species, its asexual form, the conidia, exhibit the following characteristics: 9–12 × 6–8 μm, mainly rounded in face view, mainly lenticular, brown, with an equatorial germ slit, smooth (Hyde et al. 1998), which shares the same conidial characteristics as those reported for *Apiospora* (Pintos and Alvarado 2021). Therefore, *Ar. sinense* is reinstated in *Apiospora* to accommodate this taxon.

##### Apiospora tongrenensis

Taxon classificationAnimaliaXylarialesApiosporaceae

S.Q. Guo, X.C. Wang, K.D. Hyde & Yong Wang bis sp. nov.

4FF98D5A-A06B-55AB-BA61-F8085117334B

860113

Fig. 12

###### Etymology.

Named according to Tongren, Guizhou Province, China, where the species was collected.

**Figure 12.**
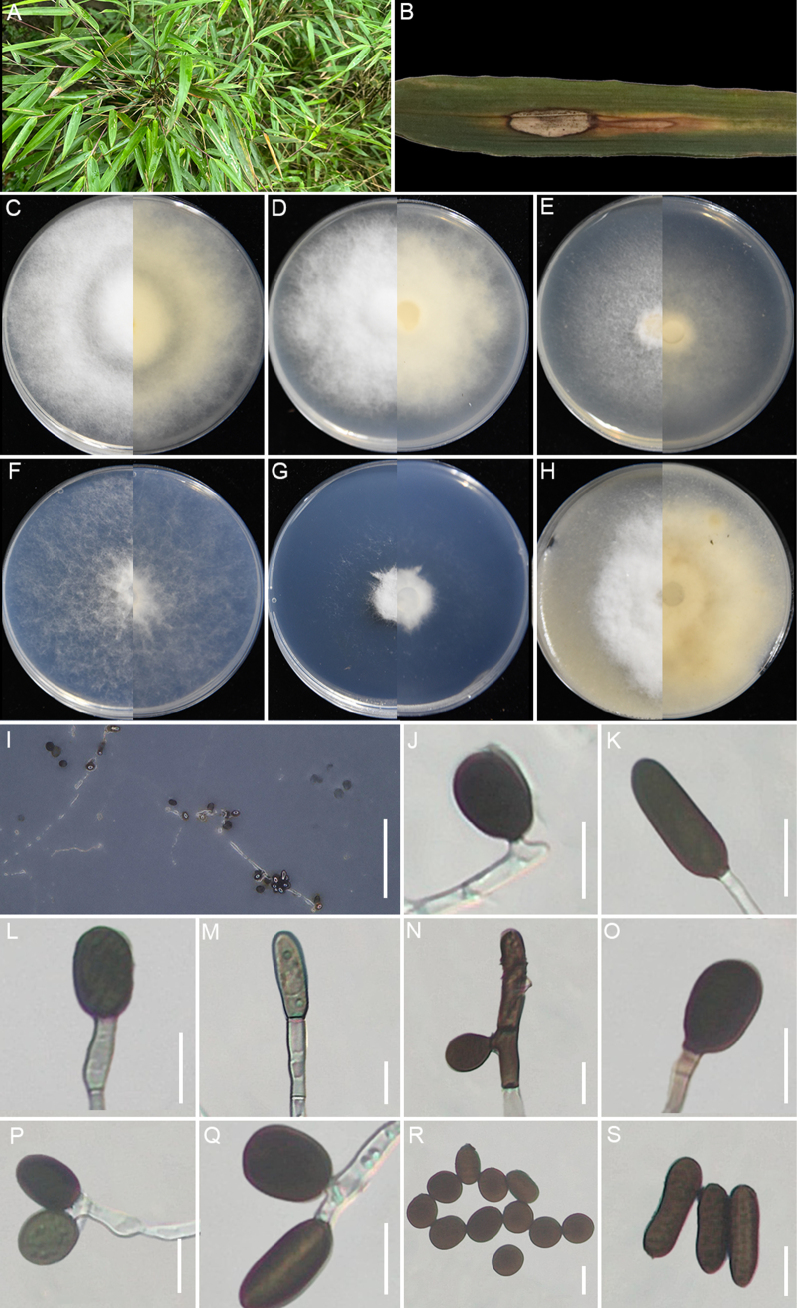
*Apiospora tongrenensis* (GUCC 25-0083). **A** Collecting location; **B** Appearance of the fungus on spot leaves of bamboo; **C–H** Upper view and reverse view of culture on PDA, MEA, CMA, SNA, WA, and OA after 7 days; **I** Close up of SNA culture with conidia. (J, L, O–Q) Conidia with conidiogenous cells on SNA. (K, M, N) Elongated conidia (sterile cells) with conidiogenous cells on SNA; **R** Conidia; **S** Elongated conidia (sterile cells). Scale bars: 100 μm (**B**); 10 μm (**J–S**).

###### Holotype.

China • Guizhou Province, Tongren City, Foding Mountain, leaf spots on bamboo, 10 July 2024, S.Q. Guo, HGUP 25-0009 (holotype), ex-type living culture GUCC 25-0083.

###### Description.

Associated with leaf spots on bamboo. Lesions as pale-yellow spots on leaves, subglobose and ellipsoidal in shape, measuring 500–2000 × 240–1200 μm. **Sexual morph**: Not observed. **Asexual morph**: On SNA, ***Hyphae*** 1.9–3.6 μm wide, branched, coiled hyphae, septate, hyaline to pale green. ***Conidiophores*** reduced to conidiogenous cells. ***Conidiogenous cells*** 6–18.4 × 3.1–6.6 μm (x̄ = 12.4 × 4.2 μm, n=30), cylindrical, aggregated, monoblastic to polyblastic, smooth, hyaline or brown. ***Conidia*** immature pale green, mature brownish, smooth-walled, globose or subglobose, 10–14.9 × 8.5–11.4 μm (x̄ = 13 × 9.7 μm, n=30), sometimes with longitudinal germ slit. Elongated conidia (sterile cells) pale brown, elongate, 17.6–32.5 × 4.5–8.8 μm (x̄ = 22.3 × 6.9 μm, n=30).

###### Culture characteristics.

Colonies after 7 days at 25°C: On PDA, colonies reach 90 mm diam., flat, spreading, dense aerial mycelia, with regular margins, surface white and reverse pale salmon. On MEA, colonies reach 80–82 mm diam., flat, cottony, dense aerial mycelia, with regular margins, surface white and reverse pale salmon. On CMA, colonies reach 77–80 mm diam., flat, spreading, with regular margins, surface white, and reverse pale salmon. On SNA, colonies reach 83–85 mm diam., flat, aerial mycelia scant, regular margins filamentous, surface and reverse white. On WA, colonies reach 47–50 mm diam., flat, center dense aerial mycelia, and margin scant, surface and reverse white. On OA, colonies reach 64–67 mm diam., flat, cottony, dense aerial mycelia, with regular margins, surface white and reverse salmon. Sporulation was abundant only on SNA after 14 days.

###### Material examined.

China • Guizhou Province, Tongren City, Foding Mountain, leaf spots on bamboo, 10 July 2024, S.Q. Guo, HGUP 25-0009 (holotype); GUCC 25-0083 (ex-type), GUCC 25-0083 and GUCC 25-0084.

###### Notes.

Phylogenetic analyses (Fig. 2) revealed that the new species, *A. tongrenensis* (GUCC 25-0083 and GUCC 25-0084) formed a distinct and well-supported branch with *A. saccharicola* (99% ML/1.0 BI). Morphologically, the two species are clearly different, with *A. tongrenensis* possessing larger conidiogenous cells (6–18.4 × 3.1–6.6 μm vs. 5–12 × 2.5–4 μm; Crous and Groenewald 2013). The conidia of *A. tongrenensis* are also larger than those of *A. saccharicola* (Crous and Groenewald 2013). Pairwise sequence comparisons between *A. tongrenensis* (GUCC 25-0083) and *A. saccharicola* (CBS 191.73) revealed nucleotide differences of 1% in ITS (6/600, no gaps), 0.82% in LSU (10/1225, no gaps), 3.47% in *tub2* (28/806, including six gaps), and 0.92% in *tef1-α* (4/435 bp, no gaps).

##### Apiospora vietnamensis

Taxon classificationAnimaliaXylarialesApiosporaceae

(Hol.-Jech.) Pintos & P. Alvarado, Fungal Syst. Evol. 7: 207. 2021.

7D2B2152-B4C6-57DE-87D3-4FC1C946A6D4

837737

###### Basionym.

*Nigrospora vietnamensis* Hol.-Jech., Česká Mykol. 17(1): 19. 1963.

###### Synonyms.

*Arthrinium euphorbiae* M.B. Ellis, Mycol. Pap. 103: 6. 1965.

*Arthrinium malaysianum* Crous, in Crous & Groenewald, IMA Fungus 4(1): 144. 2013. syn. nov.

*Arthrinium vietnamense* (Hol.-Jech.) Mei Wang & L. Cai [as ‘vietnamensis’], in Wang, Liu, et al., Persoonia 39: 139. 2017. syn. nov.

*Apiospora euphorbiae* (M.B. Ellis) X.G. Tian & Tibpromma, in Tian, Karunarathna, et al., Life 11(no. 1071): 17. 2021. syn. nov.

*Apiospora malaysiana* (Crous) Pintos & P. Alvarado, Fungal Syst. Evol. 7: 206. 2021. syn. nov.

*Apiospora magnispora* H.J. Zhao, Manawas. & W. Dong, in Zhao, Dong, et al., Curr. Res. Envir. & App. Myc. 13(1): 9. 2023. syn. nov.

###### Substrate and distribution.

On decayed fruit of *Citrus sinensis*, Czech Republic (Holubová-Jechová 1963); On dead stems of *Euphorbia*, Zambia (Ellis 1965); *Macaranga hullettii*, Malaysia (Crous and Groenewald 2013); *Cinnamomum camphora* (Crous and Groenewald 2013); *Ficus septica*, Taiwan (China) (Tennakoon et al. 2021); *Bambusa textilis*, China (Zhao et al. 2023); Bats, China (Liu et al. 2023b). Other distributions such as Vietnam, Indonesia, Brazil, Portugal, India, United Kingdom, and The United Republic of Tanzania record in GBIF.

###### Notes.

The species *A. vietnamensis* was originally described as *N. vietnamensis* from decayed fruit of *Citrus sinensis* (Jechová 1963) and later transferred to *Arthrinium* by Wang et al. (2017) before being reclassified under *Apiospora* based on DNA sequence data (Pintos and Alvarado 2021). Our phylogenetic analyses indicate that *A. vietnamensis*, *A. euphorbiae*, *A. malaysiana*, and *A. magnispora* form a single clade without evident genetic divergence (Fig. 2). Pairwise sequence comparisons further reveal negligible nucleotide differences among the type strains of these species. Morphologically, *A. euphorbiae* (Ellis 1965; Tian et al. 2021), *A. malaysiana* (Crous & Groenewald, 2013), and *A. vietnamensis* (Wang et al. 2017) share nearly identical conidial dimensions (4–5.5 × 3–4 µm, 5–6 × 3–4 µm, and 5–6 × 3–4 µm, respectively). In contrast, *A. magnispora* produces distinctly larger conidia (20–35 × 15–25 µm; Zhao et al. 2023), which may represent host-associated morphological plasticity rather than a taxonomically meaningful difference. Pairwise sequence comparisons revealed that type strain IMI 99670T of *A. vietnamensis* differs from *A. malaysiana* (CBS 102053T) by 0.18% in ITS (1/570 bp, no gaps), 0.12% in LSU (1/812 bp, one gap), and no differences in *tub2*. Comparisons with *A. euphorbiae* (IMI 285638b) showed divergences of 0.18% in ITS (1/520 bp, no gaps), 0.31% in LSU (1/318 bp), and no difference in *tub2*. Comparisons with *A. magnispora* (ZHKUCC 22-0001T) showed divergences of 0.18% in ITS (1/570 bp, no gaps), 0.12% in LSU (1/812 bp, one gap), and no differences in *tub2*. Given the absence of genetic differentiation and the strong morphological congruence among these taxa, we consider *A. euphorbiae*, *A. malaysiana*, and *A. magnispora* to be conspecific with *A. vietnamensis*, and thus propose them as its synonyms.

#### *Nigrospora* Zimm., Centbl. Bakt. ParasitKde, Abt. II 8: 220 (1902).

##### Nigrospora chinensis

Taxon classificationAnimaliaXylarialesApiosporaceae

Mei Wang & L. Cai, in Wang, Liu, et al., Persoonia 39: 129. 2017.

57ED8FD0-92F9-58B0-86A4-EB51185AA911

820732

Fig. 13

###### Substrate and distribution.

Found mostly in China on *Zanthoxylum bungeanum* (Cheng et al. 2025); dragon fruit (Guo et al. 2024); *Camellia oleifera* (Qin et al. 2021); rice, (Liu et al. 2024c); *Camellia sinensis*, *Musa paradisiaca*, *Lindera aggregate*, *Aucuba japonica*, *Machilus duthiei*, *Osmanthus* sp., *Quercus* sp., *Smilax ocreata*, (Wang et al. 2017). And on *Eleiodoxa conferta*, Thailand (Karimi et al. 2025).

**Figure 13.**
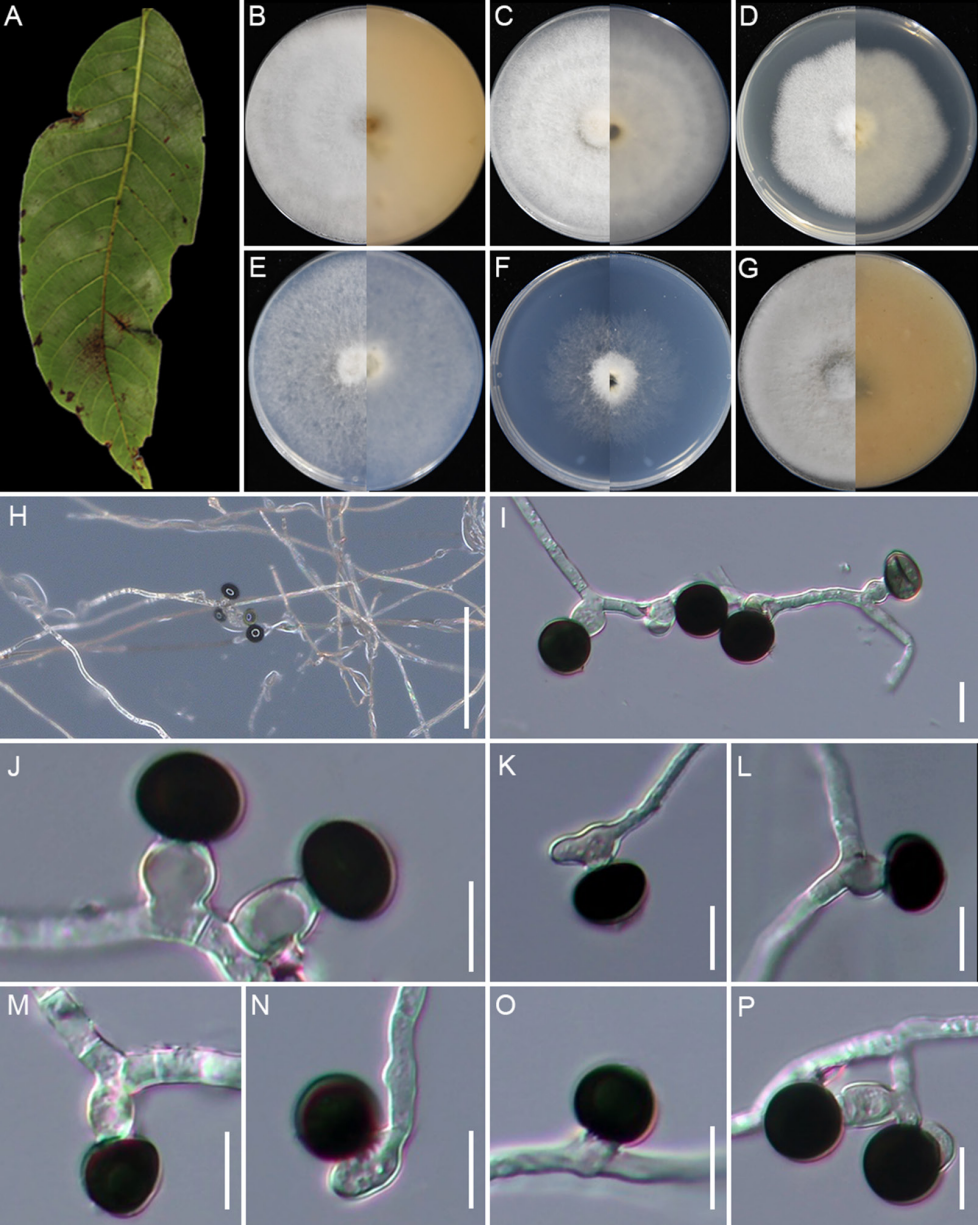
*Nigrospora chinensis* (GUCC 25-0120) **A** Appearance of the fungus on leaves of *Juglans regia*; **B–G** Upper view and reverse view of culture on PDA, MEA, CMA, SNA, WA, and OA after 7 days; **H** Close up of SNA culture with conidia; **I–P** Conidia with conidiogenous cells on SNA. Scale bars: 100 μm (**H**); 10 μm (**I–P**).

###### Description.

Associated with leaf spots on *Juglans regia*. Circular or irregular lesions, ranging from yellowish-brown to dark brown, primarily concentrated along the leaf margins. **Sexual morph**: Not observed. **Asexual morph**: On SNA, ***Hyphae*** 2.5–5.1 µm wide, hyphae, branched, septate, hyaline to pale green, smooth. ***Conidiophores*** reduced to conidiogenous cells. ***Conidiogenous cells*** 5.4–9.5 × 4.8–6.1 µm (x̄ = 7.4 × 5.3 μm, n=30), ampulliform to subglobose, monoblastic, hyaline. ***Conidia*** 10.3–13.5 × 8.5–12.8 µm (x̄ = 11.9 × 10.3 μm, n=30), globose to subglobose, solitary, aseptate, black, smooth-walled, rarely longitudinal germ-slit.

###### Culture characteristics.

Colonies after 7 days at 25 °C: On PDA, colonies reach mm 90 diam., flat, spreading, with regular margins, surface white and reverse pale salmon. On MEA, colonies reach 88–90 mm diam., flat, spreading, with regular margins, surface and reverse white. On CMA, colonies reach 70–72 mm diam., floccose, spreading, with regular margins, surface and reverse white. On SNA, colonies reach 85–87 mm diam., flat, spreading, with regular margins, surface and reverse white. On WA, colonies reach 53–57 mm diam., flat, spreading, with erose margin, surface and reverse white. On OA, colonies reach 90 mm diam., flat, cottony, with regular margins, surface pale mouse grey and reverse pale salmon. Sporulation was abundant only on SNA after 14 days.

###### Material examined.

China • Guizhou Province, Tongren City, Dejiang County, on the diseased leaves of *Juglans regia* with spots, 5 May 2024, M.T. Zou, HGUP 25-0060; living cultures GUCC 25-0120 and GUCC 25-0121.

###### Notes.

Our phylogenetic analysis revealed that the two strains GUCC 25-0120 and GUCC 25-0121 clustered together with *N. chinensis*, showing no significant genetic distance and high support rates (100% ML/1.0 BI; Fig. 3). Morphological comparison indicated that the characteristics of our strains are consistent with those reported for *N. chinensis* with the size of conidia and conidiogenous cells (Wang et al. 2017). This species is reported for the first time on the leaves of *Juglans regia*.

##### Nigrospora endophytica

Taxon classificationAnimaliaXylarialesApiosporaceae

A.C.Q. Brito & A.R. Machado, in Brito, Mello, et al., Mycological Progress 22(6, no. 37): 5. 2023.

B5809F7E-410E-5E2B-933D-EE12653B7682

845749

Fig. 14

###### Substrate and distribution.

*Manihot esculenta*, Brazil (de Queiroz Brito et al. 2023).

**Figure 14.**
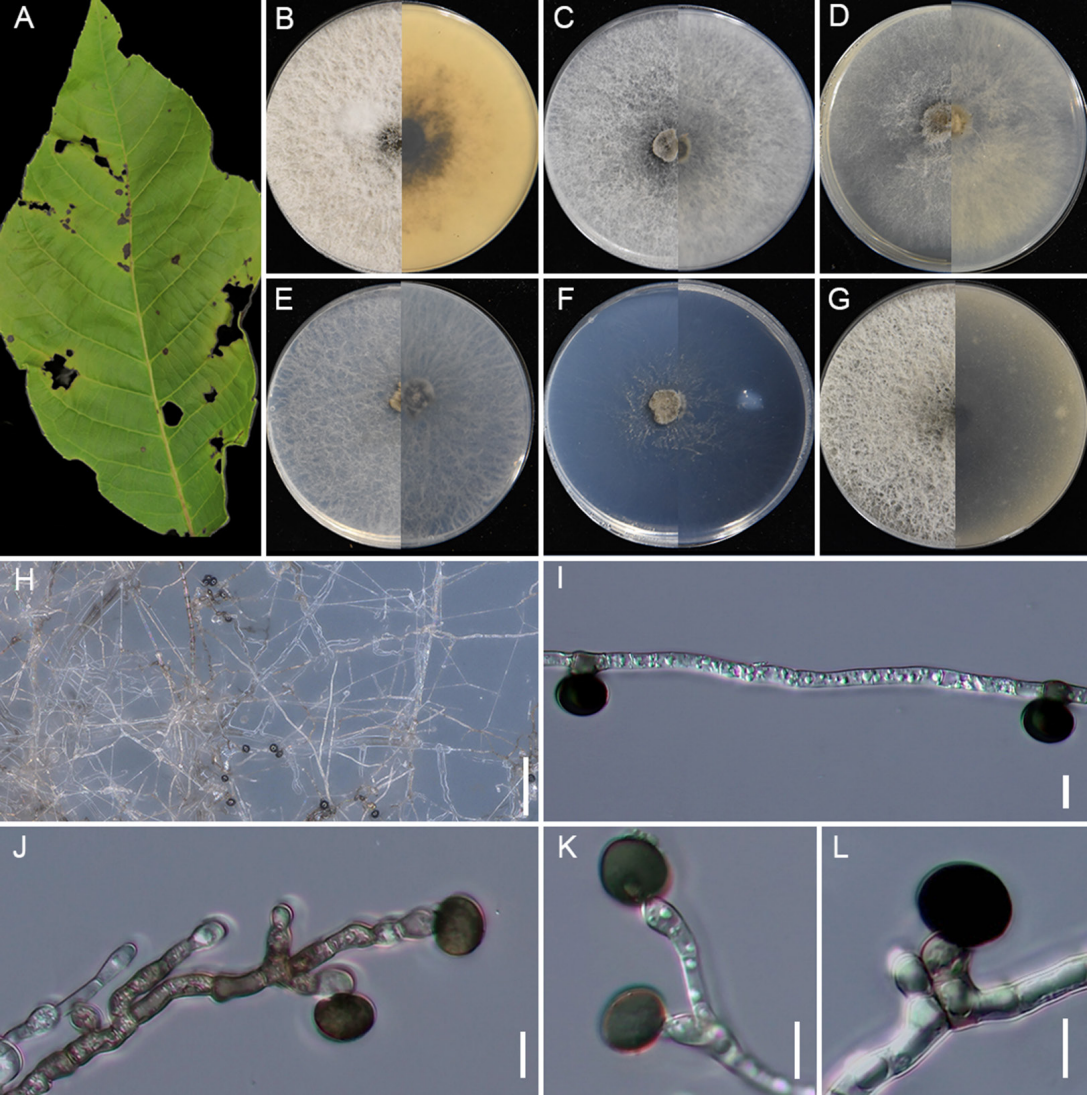
*Nigrospora endophytica* (GUCC 25-0122) **A** Appearance of the fungus on leaves of *Juglans regia*; **B–G** Upper view and reverse view of culture on PDA, MEA, CMA, SNA, WA, and OA after 7 days; **H** Close up of SNA culture with conidia; **I–L** Conidia with conidiogenous cells on SNA. Scale bars: 100 μm (**H**); 10 μm (**I–L**).

###### Description.

Associated with leaf spots on *Juglans regia*. Circular or irregular lesions, ranging from yellowish-brown to dark brown. **Sexual morph**: Not observed. **Asexual morph**: On SNA, ***Hyphae*** 1.5–4 µm wide, hyphae, ranched, sometimes coiled, septate, hyaline or pale green to dark brown. ***Conidiophores*** reduced to conidiogenous cells. ***Conidiogenous cells*** 7.4–9.1 × 4.9–7.8 µm (x̄ = 8.2 × 6.2 μm, n=30), ampulliform to subglobose, solitary, monoblastic, pale green. ***Conidia*** 11.1–16.3 × 7.4–13.4 µm (x̄ = 13.6 × 10.9 μm, n=30), subglobose, solitary, aseptate, pale brown to black, smooth.

###### Culture characteristics.

Colonies for 7 days at 25 °C: On PDA, colonies reach 90 mm diam., flat, spreading, with regular margins, surface center fuscous black and margin white, reverse center black and margin pale salmon. On MEA, colonies reach 90 mm diam., flat, spreading, with regular margins, surface center and reverse vinaceous grey and margin greyish lilac. On CMA, colonies reach 90 mm diam., flat spreading, with regular margins, surface, and reverse greyish lilac. On SNA, colonies reaching 90 mm diam., flat, aerial mycelia scant, regular margins filamentous, surface and reverse pale vinaceous grey. On WA, colonies reach 40–42 mm diam., flat, aerial mycelia scant, with erose margin, surface and reverse greyish. On OA, colonies reach 90 mm diam., flat, spreading, with regular margins, surface greyish lilac and reverse center black and margin pale grey. Sporulation was abundant only on SNA after 14 days.

###### Material examined.

China • Yunnan Province, Lincang City, on the diseased leaves of *Juglans regia* with spots, 21 June 2024, M.T. Zou, HGUP 25-0061; GUCC 25-0122 and GUCC 25-0123.

###### Notes.

Our phylogenetic analysis indicated that strains GUCC 25-0122 and GUCC 25-0123 clustered together with *N. endophytica* (strains A.R.M 973 and A.R.M 687) with statistical support (96% ML/0.69 BI) (Fig. 3). Comparison of nucleotide differences between GUCC 25-0122 and *N. endophytica* (A.R.M 973) revealed minor variations: 0.83% in ITS (4/481bp, including one gap), 1.05% in *tef1-α* (5/478 bp, including one gap), and 0.05% in *tub2* (2/405 bp, without gaps). Although slight sequence differences were observed, the morphological characteristics of our strains are closely similar to those of *N. endophytica* (de Queiroz Brito et al. 2023). Therefore, strains GUCC 25-0122 and GUCC 25-0123 are identified as *N. endophytica*. Previously, *N. endophytica* was reported only as an endophyte from the stem tissue of *Manihot esculenta* (de Queiroz Brito et al. 2023). This study represents the first record of this species from *J. regia* leaves, indicating a new host association.

##### Nigrospora neosaccharicola

Taxon classificationAnimaliaXylarialesApiosporaceae

M. T. Zou, X. C. Wang, K.D. Hyde & Yong Wang bis sp. nov.

41880857-C8AB-5139-A19A-60DB52983537

860114

Fig. 15

###### Etymology.

The species was named based on morphological similarity to *Nigrospora saccharicola*.

**Figure 15.**
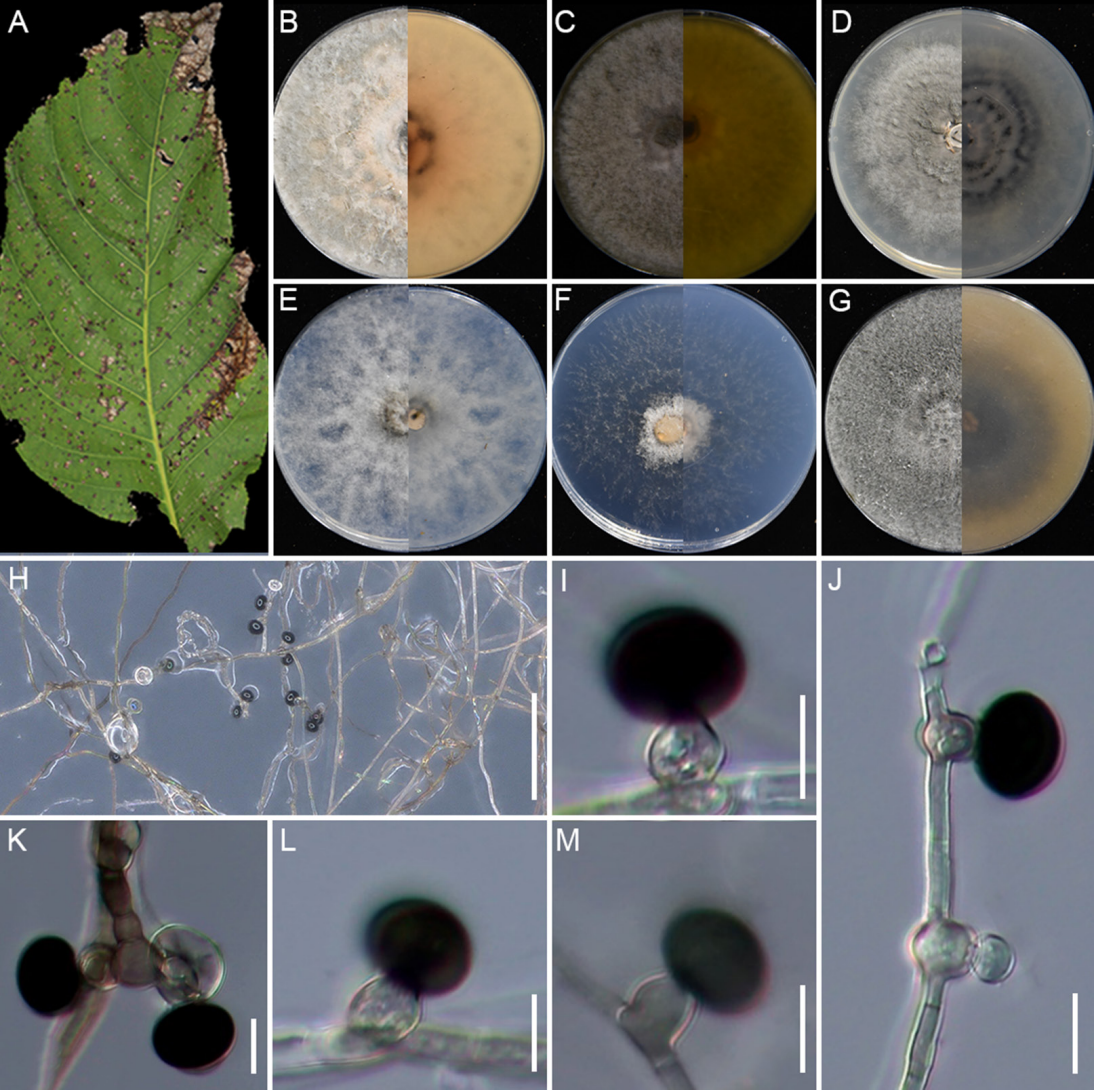
*Nigrospora neosaccharicola* (GUCC 25-0124) **A** Appearance of the fungus on leaves of *Juglans regia*; **B–G** Upper view and reverse view of culture on PDA, MEA, CMA, SNA, WA, and OA after 7 days; **H** Close up of SNA culture with conidia; **I–M** Conidia with conidiogenous cells on SNA. Scale bars: 100 μm (**H**); 10 μm (**I–M**).

###### Holotype.

China • Yunnan Province, Chuxiong City, on the diseased leaves of *Juglans regia* with spots, 26 March 2024, M.T. Zou, HGUP 25-0010 (holotype), ex-type GUCC 25-0124.

###### Description.

Associated with leaf spots on *Juglans regia*. Leaves lose their green coloration, developing nearly circular lesions that appear yellowish-brown with a surrounding yellow halo. In later stages, the center of the lesions turns pale yellow. Small spots merge into larger ones, eventually causing leaf death. **Sexual morph**: Not observed. **Asexual morph**: On SNA, ***Hyphae*** 3.2–5.8 µm wide, branched, septate, hyaline to dark brown, smooth. ***Conidiophores*** reduced to conidiogenous cells. ***Conidiogenous cells*** 4.5–10.7 × 5.4–8.9 µm (x̄ = 7.4 × 6.7 μm, n=30), subglobose to pot-shaped, aseptate, pale brown. ***Conidia*** 11.4–16.7 × 8.1–13.4 µm (x̄ = 13.7 × 10.6 μm, n=30), globose to subglobose, solitary, aseptate, brown to black, smooth-walled.

###### Culture characteristics.

Colonies after 7 days at 25 °C: On PDA, colonies reach 90 mm diam., flat, dense aerial mycelia, with regular margins, surface pale olivaceous gray, reverse center black, and margin pale flesh. On MEA, colonies reach 90 mm in diam., flat, spreading, and dense, with regular margins; surface dark mouse grey and reverse umber. On CMA, colonies reach 90 mm diam., flat, spreading, with regular margins, surface, and reverse center grey and margin greyish lilac. On SNA, colonies reach 90 mm diam., flat, aerial mycelia scant, regular margins filamentous, surface and reverse pale vinaceous grey. On WA, colonies reach 77–79 mm diam., flat, aerial mycelia scant, with erose margin, surface and reverse greyish. On OA, colonies reach 90 mm diam., cottony, dense aerial mycelia, with regular margins, surface mouse grey, reverse center dark mouse grey, and margin saffron. Sporulation was abundant only on SNA after 14 days.

###### Material examined.

China • Yunnan Province, Chuxiong City, on the diseased leaves of *Juglans regia* with spots, 26 March 2024, M.T. Zou, HGUP 25-0010 (holotype), GUCC 25-0124, (ex-type), GUCC 25-0125, GUCC 25-0126, GUCC 25-0127, GUCC 25-0128 and GUCC 25-0129.

###### Notes.

In the phylogenetic analyses (Fig. 3), six strains (GUCC 25-0124, GUCC 25-0125, GUCC 25-0126, GUCC 25-0127, GUCC 25-0128, and GUCC 25-0129) formed a sister clade to *N. saccharicola* (type strain CGMCC 3.19362) with strong statistical support (100% ML/1.0 BI). The comparison of nucleotide differences between strain GUCC 25-0124 and the type strain of *N. saccharicola* revealed clear sequence divergence: 0.31% in ITS (1/324 bp, no gaps), 3.32% in *tub2* (12/361 bp, including seven gaps), and 5.58% in *tef1-α* (24/430 bp, including three gaps). Morphologically, our isolates differ slightly from *N. saccharicola* in both conidial and conidiogenous cell dimensions. *Nigrospora neosaccharicola* produces smaller conidia (mean = 13.7 × 10.6 μm vs. 15.24 × 11.69 μm) and narrower conidiogenous cells (mean = 7.4 × 6.7 μm vs. 8.65 × 6.8 μm) (Raza et al. 2019). Furthermore, sterile (elongated) cells, which are present in *N. saccharicola*, were not observed in our isolates. While *N. saccharicola* was originally described from *Saccharum officinarum* (Raza et al. 2019), our isolates were obtained from leaves of *Juglans regia*.

##### Nigrospora osmanthi

Taxon classificationAnimaliaXylarialesApiosporaceae

Mei Wang & L. Cai, Persoonia 39: 135 (2017).

A2EDC710-48D0-56E4-A238-21197035B59E

820736

Fig. 16

###### Substrate and distribution.

*Ficus pandurata*, China (Liu et al. 2019); *Hedera nepalensis*, China (Wang et al. 2017); Water Lettuce, China (Lin et al. 2023); *Stenotaphrum secundatum* China (Mei et al. 2019); *Orthosiphon stamineus*, Malaysia (Ismail et al. 2022); *Artemisia argyi*, China (Yang et al. 2025); *Cirsium setosum*, *Phyllostachys nigra*, *Phragmites australis*, *Rosa chinensis*, China (Hao et al. 2020). Other distributions such as Colombia, Croatia, Iran, Malaysia, Saudi Arabia, South Africa, and United States recorded in GBIF.

**Figure 16.**
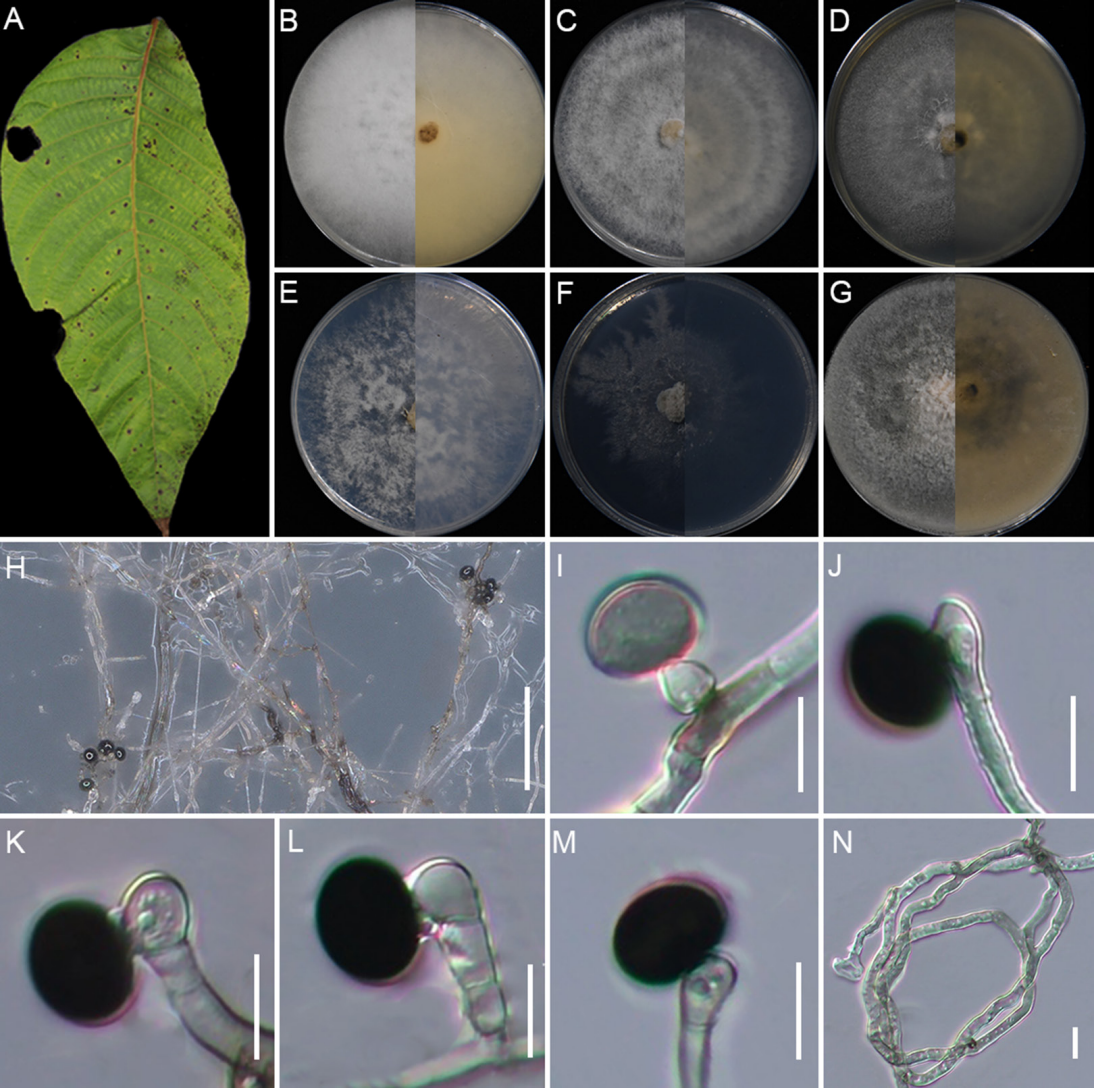
*Nigrospora osmanthi* (GUCC 25-0130). **A** Appearance of the fungus on leaves of *Juglans regia*; **B–G** Upper view and reverse view of culture on PDA, MEA, CMA, SNA, WA, and OA after 7 days; **H** Close up of SNA culture with conidia; **I–M** Conidia with conidiogenous cells on SNA; **N** Coiled hyphae on SNA. Scale bars: 100 μm (**H**); 10 μm (**I–N**).

###### Description.

Associated with leaf spots of *Juglans regia*. Circular or irregular lesions, ranging from yellowish-brown to dark brown with yellow halos. **Sexual morph**: Not observed. **Asexual morph**: On SNA, ***Hyphae*** 3.5–7 µm wide, branched, septate, hyaline to dark brown, smooth. ***Conidiophores*** reduced to conidiogenous cells. ***Conidiogenous cells*** 5.8–8.7 × 4.1–6.6 µm (x̄ = 7.0 × 5.2 μm, n=30), subglobose, monoblastic, pale green to brown. ***Conidia*** 10.2–12.3 × 8.3–10.9 µm (x̄ = 11.4 × 9.5 μm, n=30), globose to subglobose, aseptate, solitary, brown to black, smooth, shiny.

###### Culture characteristics.

Colonies after 7 days at 25 °C: On PDA, colonies reach 90 mm diam., flat, spreading, dense aerial mycelia, with regular margins, surface and reverse white. On MEA, colonies reach 90 mm diam., flat, spreading, dense aerial mycelia, regular margins, surface and reverse pale vinaceous grey. On CMA, colonies reach 90 mm diam., flat, spreading, regular margins, surface and reverse pale vinaceous grey. On SNA, colonies reach 88–90 mm diam., flat, spreading, aerial mycelia scant, regular margins filamentous, surface and reverse pale vinaceous grey. On WA, colonies reach 52–55 mm diam., flat, spreading, with erose and irregular margin, surface and reverse pale vinaceous grey. On OA, colonies reach 90 mm diam., flat, spreading, dense aerial mycelia, with regular margins, surface mouse grey and reverse center dark mouse grey and margin saffron. Sporulation was abundant only on SNA after 14 days.

###### Material examined.

China • Yunnan Province, Chuxiong City, on the diseased leaves of *Juglans regia* with spots, 23 March 2024, M.T. Zou, HGUP 25-0062; GUCC 25-0130, GUCC 25-0131, GUCC 25-0132 and GUCC 25-0133.

###### Notes.

*Nigrospora osmanthi* was first observed on *Osmanthus* sp. in Jiangxi Province, China (Wang et al. 2017). In this study, four strains (GUCC 25-0130, GUCC 25-0131, GUCC 25-0132, and GUCC 25-0133) were isolated from leaf spots of *J. regia*. Phylogenetic analysis revealed that these two strains clustered with the type strain of *N. osmanthi* (CGMCC 3.18126) and strain LC4467, showing no significant genetic divergence. Morphologically, our isolates exhibited characteristics consistent with those described for *N. osmanthi* (Wang et al. 2017). Based on the combined morphological and phylogenetic evidence, the four strains are identified as *N. osmanthi*. This represents the first record of *N. osmanthi* from *J. regia*. However, further pathogenicity tests are required to confirm the pathogenic causes of leaf spot symptoms.

##### Nigrospora sphaerica

Taxon classificationAnimaliaXylarialesApiosporaceae

(Sacc.) E.W. Mason, Trans. Br. Mycol. Soc. 12(2-3): 158 (1927).

8708EAD7-9D28-5539-BE6C-8941DF9AD3F8

254776

Fig. 17

###### Substrate and distribution.

Pathogens with a wide host range, primarily causing leaf spot or leaf blight.

**Figure 17.**
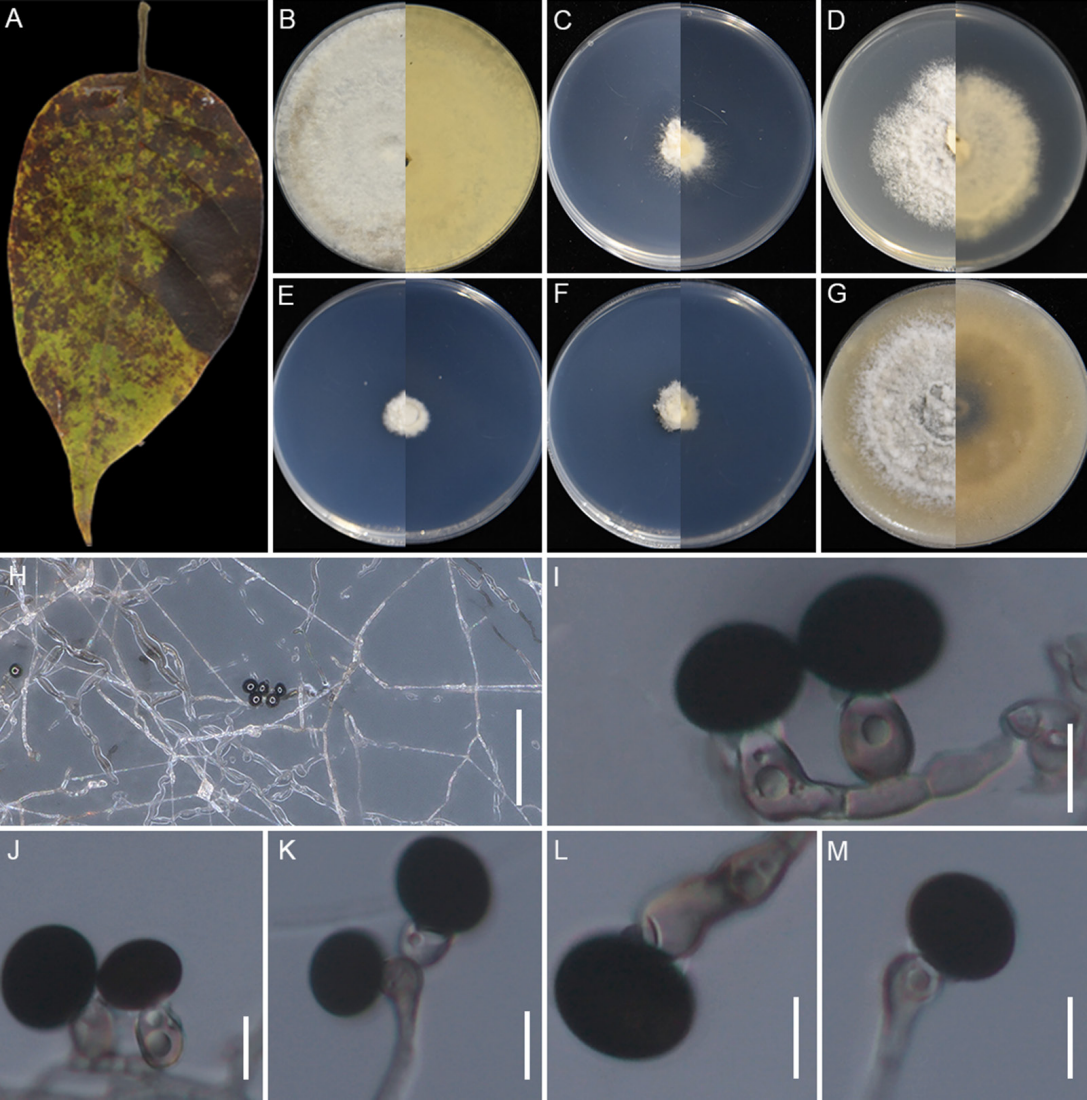
*Nigrospora sphaerica* (GUCC 25-0134). **A** Appearance of the fungus on leaves of *Juglans regia*; **B–G** Upper view and reverse view of culture on PDA, MEA, CMA, SNA, WA, and OA after 7 days; **H** Close up of SNA culture with conidia; **I–M** Conidia with conidiogenous cells on SNA. Scale bars: 100 μm (**H**); 10 μm (**I–M**).

###### Description.

Associated with leaf spots of *Juglans regia*. Lesions irregular in shape and dark brown in color. **Sexual morph**: Not observed. **Asexual morph**: On SNA, ***Hyphae*** 2.5–5 µm in diam., branched, septate, contracted at septum, hyaline to dark brown, smooth. ***Conidiophores*** reduced to conidiogenous cells. ***Conidiogenous cells*** 6.0–10.5 × 5.0–8.1 µm (x̄ = 7.8 × 6.6 μm, n=30), subglobose, monoblastic, pale brown. ***Conidia*** 11.8–15.8 × 9.9–13.7 µm (x̄ = 14.1 × 11.6 μm, n=30), globose to subglobose, solitary, aseptate, black, smooth.

###### Culture characteristics.

Colonies after 7 days at 25 °C: On PDA, colonies reach 90 mm diam., flat, spreading, with regular margins, surface white and reverse salmon. On MEA, colonies reach 27–30 mm diam., flat, aerial mycelia scant, regular margins filamentous, surface and reverse white. On CMA, colonies reach 56–60 mm diam., cottony, dense aerial mycelia, with regular margins, surface white and reverse pale salmon. On SNA, colonies reach 20–22 mm diam., flat, aerial mycelia scant, regular margins filamentous, surface and reverse white. On WA, colonies reach 20–22 mm diam., flat, aerial mycelia scant, regular margins filamentous, surface and reverse white. On OA, colonies reach 73–75 mm diam., flat, spreading, dense aerial mycelia, with regular margins, surface greyish lilac and reverse center black and margin pale salmon. Sporulation was abundant only on SNA after 14 days.

###### Material examined.

China • Yunnan Province, Lincang City, on the diseased leaves of *Juglans regia* with spots, 7 May 2024, M.T. Zou, HGUP 25-0063; GUCC 25-0134, GUCC 25-0135, GUCC 25-0136, GUCC 25-0137, GUCC 25-0138 and GUCC 25-0139.

###### Notes.

In the phylogenetic analyses (Fig. 3), the six strains obtained in this study (GUCC 25-0134, GUCC 25-0135, GUCC 25-0136, GUCC 25-0137, GUCC 25-0138, and GUCC 25-0139) clustered with the *N. sphaerica* species clade with strong statistical support (100% ML/1.0 BI). Morphologically, our isolates closely resemble *N. sphaerica* in their overall diagnostic characteristics. The conidia of our strains are comparable in shape but slightly smaller (14.1 × 11.6 µm) than those reported for *N. sphaerica* (18.22 × 18.22 µm), while the conidiogenous cells also show similar dimensions (7.8 × 6.6 µm vs. 7.97 × 7.97 µm; Wang et al. 2017; Hyde et al. 2024a). This study represents the first record of *N. sphaerica* isolated from leaves of *J. regia*.

## Discussion

The family *Apiosporaceae* is widely distributed across terrestrial ecosystems and exhibits broad ecological adaptability, with members recorded from tropical, subtropical, temperate, and boreal regions and colonizing diverse hosts with no clear host specificity (Jiang et al. 2019; Phukhamsakda et al. 2022). The Yunnan–Guizhou Plateau in Southwest China represents a well-known biodiversity hotspot where numerous novel fungi have been discovered in recent years (Wijayawardene et al. 2021), including members of *Apiosporaceae*, such as *A. aseptata*, *A. dematiacea*, *A. dicranopteridis*, *A. globosa* (Zhang et al. 2023), *A. olivata* (Zhang et al. 2024b), and *N. weininensis* (Liu et al. 2024a). In this study, all specimens were collected from Guizhou and Yunnan provinces, further confirming that the plateau remains an important reservoir of *Apiosporaceae* diversity.

The multilocus phylogenetic analysis using ITS, LSU, *tef1-α*, and *tub2* sequences confirms the novelty of these taxa. Four single-gene phylogenetic trees were constructed to evaluate variation in the ITS and protein-coding genes (*tef1-α* and *tub2*). In the ITS phylogeny (Suppl. material 1: fig. S1), several *Apiospora* species exhibited low genetic divergence, whereas this phenomenon is less frequent in *tub2* and *tef1-α* phylogenies (Suppl. material 1: figs S3, S4). These results indicate that *tub2* and *tef1-α* provide higher resolution for species delimitation than ITS, consistent with Monkai et al. (2022). Similarly, in a study of *Hypoxylaceae*, another family within the *Xylariomycetidae* to which *Apiospora* belongs, Stadler et al. (2020) demonstrated that ITS alone is insufficient for delimiting taxa, as ITS and other rDNA markers exhibit polymorphism both between and within species. Therefore, species identification in *Apiospora* should rely on multigene phylogenetic frameworks, with a particular emphasis on *tub2* and *tef1-α* genes, while ITS is primarily suitable for genus-level identification.

Multigene phylogenetic analyses demonstrate that *A. mediterranea* and *A. hispanica* are conspecific, forming a single clade with no genetic divergence and showing minimal morphological differentiation (Monkai et al. 2022; Liao et al. 2023). Similarly, *A. euphorbiae*, *A. magnispora*, and *A. malaysiana* should be treated as synonyms of *A. vietnamensis*, as they cluster together with negligible nucleotide differences and minimal morphological variation. Phylogenetic analyses in *Nigrospora* revealed that *N. sphaerica* is divided into two lineages, one of which includes only strains LC2839 and LC2840. The type strain lacks molecular data, and the sequences provided by Wang et al. (2017) indicate that LC2840 forms a separate branch from the main *N. sphaerica* clade. Comparative sequence analyses reveal differences of 1.94% in *tub2* (8/412 bp, five gaps) and 2.55% in *tef1-α* (12/470 bp, no gap) between LC2840 and LC7295. BLAST analyses further indicate that these strains share <99% identity with known *Nigrospora* sequences. Therefore, resequencing of LC2840 and LC2839 is recommended to determine whether these strains represent a distinct taxon or belong to *N. sphaericasensu stricto*.

To investigate spore production in *Apiospora* and *Nigrospora*, six commonly used media (PDA, SNA, WA, OA, CMA, and MEA) were evaluated, and conidia production was examined after 14 days. *Apiospora* produced conidia readily on WA and SNA, consistent with the report by Zhang et al. (2023). Strains of *A. setariae* failed to produce conidia on any medium, even after 30 days. For *Nigrospora*, conidia production occurred only on SNA medium after 14 days, though at low levels. In addition, these observations suggest that certain cultural characteristics may serve as diagnostic features for species identification. For example, *A. huaxiensis* and *A. setariae* produce red pigments on PDA. Similar pigment production has been reported in *A. dehongensis* (Han et al. 2024). Therefore, red pigment production on PDA may represent a useful taxonomic character.

The four new species of *Apiospora* were isolated from symptomatic bamboo leaves or stems, supporting a strong association with *Poaceae*, particularly bamboo hosts (Han et al. 2024; Liu et al. 2024b). Two additional taxa, *A. locuta-pollinis* and *A. setariae* were recorded from new host species. Strains of *A. locuta-pollinis*, previously reported in hive-stored pollen (Zhao et al. 2018), *Aristolochia debilis* (Chen et al. 2021), bamboo (Monkai et al. 2022), *Musa* sp. (Samarakoon et al. 2024), and grass (Gao et al. 2025), were isolated from maize leaves. Moreover, *A. setariae*, previously known from dead culms of *Setaria viridis* (Jiang and Tian 2021), is recorded here for the first time from bamboo. All *Nigrospora* isolates in this research were obtained from walnut. To date, only one species, *N. yunnanensis*, has been reported from walnut (Zou et al. 2024). Analyses of host preferences and species distributions of *Apiospora* and *Nigrospora* indicate that for *Apiospora*, the primary host family is *Poaceae*, with bamboo species constituting the largest proportion, consistent with Monkai et al. (2022). For the species distributions of this genus, Monkai et al. (2022) identified China as the country with the highest number of recorded species for this genus. Our findings, however, indicate that the United States has the highest number of recorded species, followed by the United Kingdom and China. This discrepancy likely reflects differences between databases, as GBIF was used, whereas Monkai et al. (2022) used USDA data, which contain fewer records and are primarily disease-related. For *Nigrospora*, GBIF data indicate that *Poaceae* is the most common host family, followed by *Fabaceae* and *Cyperaceae*. In terms of geographic distribution, China has the highest number of hosts records, followed by India and the United States.

Overall, this study significantly expands current knowledge of *Apiosporaceae* diversity in Southwest China, clarifies several taxonomic relationships within *Apiospora and Nigrospora*, and highlights the Yunnan–Guizhou Plateau as a rich reservoir of new taxa. The discovery of five new species and six new host species records highlights the ecological adaptability of *Apiosporaceae* and underscores the importance of integrating morphology, molecular data, and host associations for accurate species identification and improved understanding of their taxonomy and evolution.

## Supplementary Material

XML Treatment for Apiospora hispanica

XML Treatment for Apiospora huaxiensis

XML Treatment for Apiospora locuta-pollinis

XML Treatment for Apiospora nanmingensis

XML Treatment for Apiospora qingzhenensis

XML Treatment for Apiospora setariae

XML Treatment for Apiospora sinense

XML Treatment for Apiospora tongrenensis

XML Treatment for Apiospora vietnamensis

XML Treatment for Nigrospora chinensis

XML Treatment for Nigrospora endophytica

XML Treatment for Nigrospora neosaccharicola

XML Treatment for Nigrospora osmanthi

XML Treatment for Nigrospora sphaerica

## Additional information

### Artificial Intelligence (AI) use

The authors accept full responsibility for the content of the manuscript, including the disclosure of any use of AI.

No AI tools were used in the preparation of this manuscript.

### Adherence to national and international regulations

All the fungal strains used in this study have been legally obtained, respecting the Convention on Biological Diversity (Rio Convention).

### Author ORCIDs

Xing-Chang Wang https://orcid.org/0009-0008-4913-5316

Rui-Nan Zhang https://orcid.org/0009-0009-6010-9570

Chada Norphanphoun https://orcid.org/0000-0002-5756-7206

Si-Bo Wang https://orcid.org/0009-0004-0060-3247

Meng-Ting Zou https://orcid.org/0009-0005-5564-4131

Shi-Qi Guo https://orcid.org/0009-0002-4997-2276

Jing-E Sun https://orcid.org/0000-0002-5226-5743

Jia-Ping Wang https://orcid.org/0009-0002-7558-4760

Nalin N. Wijayawardene https://orcid.org/0000-0003-0522-5498

Kevin D. Hyde https://orcid.org/0000-0002-2191-0762

Yong Wang https://orcid.org/0000-0003-3831-2117

Feng-Quan Liu https://orcid.org/0000-0001-9325-1500

### Data availability

All data generated or analyzed during this study are included in this published article or in the supplementary materials. All sequences during this study have been submitted to GenBank.

## Supplementary materials

Supplementary material 1 Single gene trees for *Apiospora* and *Nigrospora* https://doi.org/10.3897/imafungus.17.177246.suppl1 Data type zip Explanation note **fig. S1**. Phylogenetic trees constructed using Maximum likelihood (ML) analyses based on ITS for *Apiospora*. **fig. S2**. Phylogenetic trees constructed using Maximum likelihood (ML) analyses based on LSU for *Apiospora*. **fig. S3**. Phylogenetic trees constructed using Maximum likelihood (ML) analyses based on *tef1-α* for *Apiospora*. **fig. S4**. Phylogenetic trees constructed using Maximum likelihood (ML) analyses based on *tub2* for *Apiospora*. **fig. S5**. Phylogenetic trees constructed using Maximum likelihood (ML) analyses based on ITS for *Nigrospora*. **fig. S6**. Phylogenetic trees constructed using Maximum likelihood (ML) analyses based on *tef1-α* for *Nigrospora*. **fig. S7**. Phylogenetic trees constructed using Maximum likelihood (ML) analyses based on *tub2* for *Nigrospora*. https://binary.pensoft.net/file/1605922This dataset is made available under the Open Database License (http://opendatacommons.org/licenses/odbl/1.0/). The Open Database License (ODbL) is a license agreement intended to allow users to freely share, modify, and use this Dataset while maintaining this same freedom for others, provided that the original source and author(S) are credited. Xing-Chang Wang, Rui-Nan Zhang, Chada Norphanphoun, Si-Bo Wang, Meng-Ting Zou, Shi-Qi Guo, Jing-E Sun, Jia-Ping Wang, Nalin N. Wijayawardene, Kevin D. Hyde, Yong Wang, Feng-Quan Liu

Supplementary material 2 Suppl. data 1-7: the sequences after mafft for *Apiospora* and *Nigrospora* https://doi.org/10.3897/imafungus.17.177246.suppl2 Data type zip Explanation note **suppl. data 1**. The ITS sequences for *Apiospora* after Mafft. **suppl. data 2**. The LSU sequences for *Apiospora* after Mafft. **suppl. data 3**. The *tef1-α* sequences for *Apiospora* after Mafft. **suppl. data 4**. The *tub2* sequences for *Apiospora* after Mafft. **suppl. data 5**. The ITS sequences for *Nigrospora* after Mafft. **suppl. data 6**. The *tef1-α* sequences for *Nigrospora* after Mafft. **suppl. data 7**. The *tub2* sequences for *Nigrospora* after Mafft. https://binary.pensoft.net/file/1605923This dataset is made available under the Open Database License (http://opendatacommons.org/licenses/odbl/1.0/). The Open Database License (ODbL) is a license agreement intended to allow users to freely share, modify, and use this Dataset while maintaining this same freedom for others, provided that the original source and author(S) are credited. Xing-Chang Wang, Rui-Nan Zhang, Chada Norphanphoun, Si-Bo Wang, Meng-Ting Zou, Shi-Qi Guo, Jing-E Sun, Jia-Ping Wang, Nalin N. Wijayawardene, Kevin D. Hyde, Yong Wang, Feng-Quan Liu

Supplementary material 3 Primer pairs and amplification procedures used in this study, the best models of BI and ML used for each gene in the phylogenetic tree, and the records of *Apiospora* and *Nigrospora* in the GBIF database https://doi.org/10.3897/imafungus.17.177246.suppl3 Data type xlsx Explanation note **table SS1**. Primer pairs and amplification procedures used in this study. **table S2**. The best models of BI and ML used for each gene in the phylogenetic tree. **table S3**. Records of *Apiospora* in GBIF. **table S4**. Records of *Nigrospora* in GBIF. https://binary.pensoft.net/file/1605924This dataset is made available under the Open Database License (http://opendatacommons.org/licenses/odbl/1.0/). The Open Database License (ODbL) is a license agreement intended to allow users to freely share, modify, and use this Dataset while maintaining this same freedom for others, provided that the original source and author(S) are credited. Xing-Chang Wang, Rui-Nan Zhang, Chada Norphanphoun, Si-Bo Wang, Meng-Ting Zou, Shi-Qi Guo, Jing-E Sun, Jia-Ping Wang, Nalin N. Wijayawardene, Kevin D. Hyde, Yong Wang, Feng-Quan Liu
