# Supplementary figures and images for: Novel species and new host records of Apiospora and Nigrospora (Amphisphaeriales, Apiosporaceae) from Yunnan-Guizhou Plateau, China
Source: IMA Fungus. 2026 Apr 24;17:e177246. doi: 10.3897/imafungus.17.177246 (PMC13135200; doi:10.3897/imafungus.17.177246)

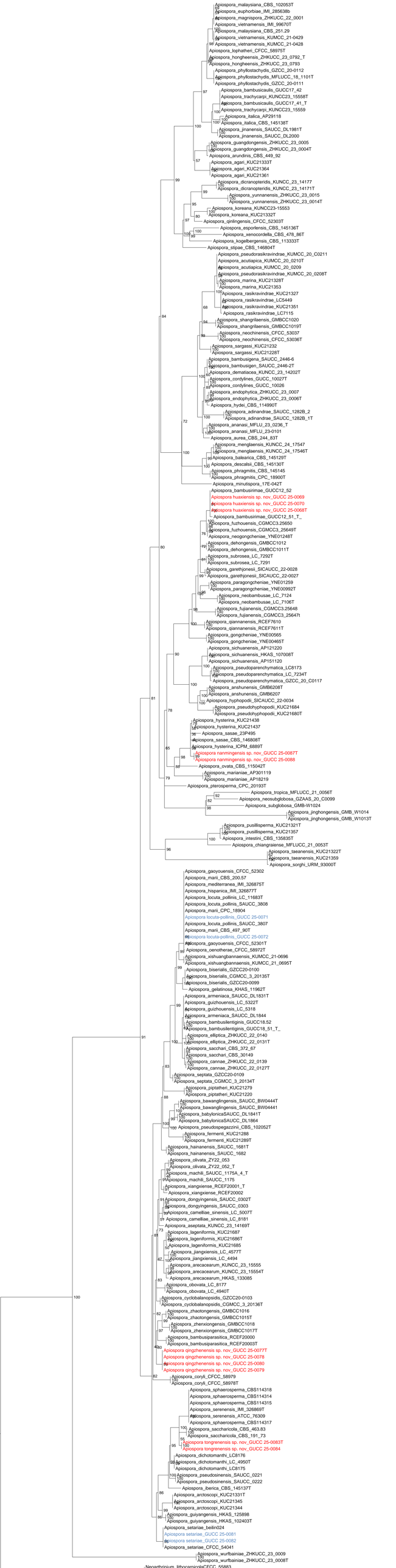

Supplement: Supplementary material 1 — Single gene trees for Apiospora and Nigrospora [file imafungus-17-e177246-s001.zip › Supplementary figure 4 Apiospora tub ML.pdf]

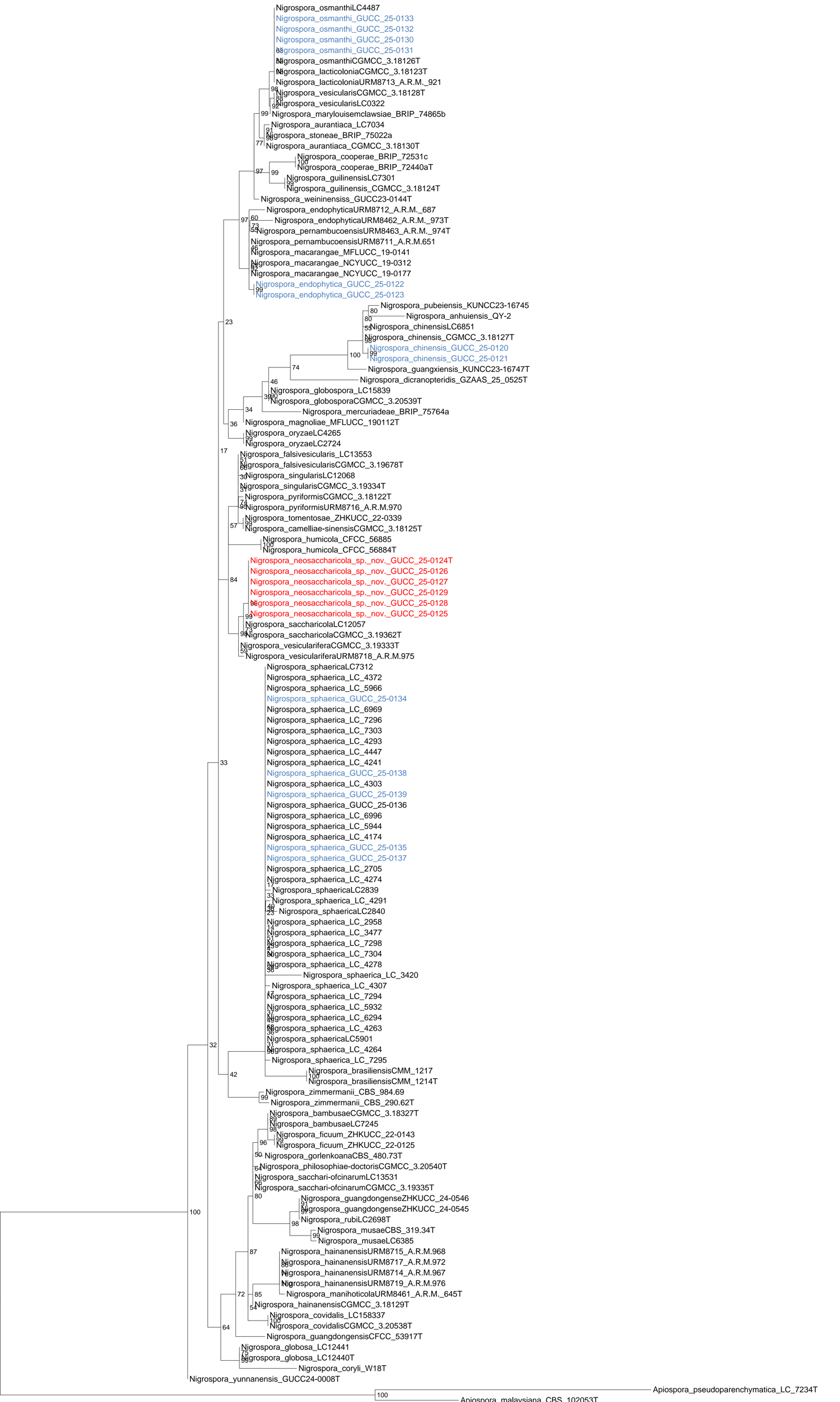

Supplement: Supplementary material 1 — Single gene trees for Apiospora and Nigrospora [file imafungus-17-e177246-s001.zip › Supplementary figure 5 Nigrospora ITS ML.pdf]

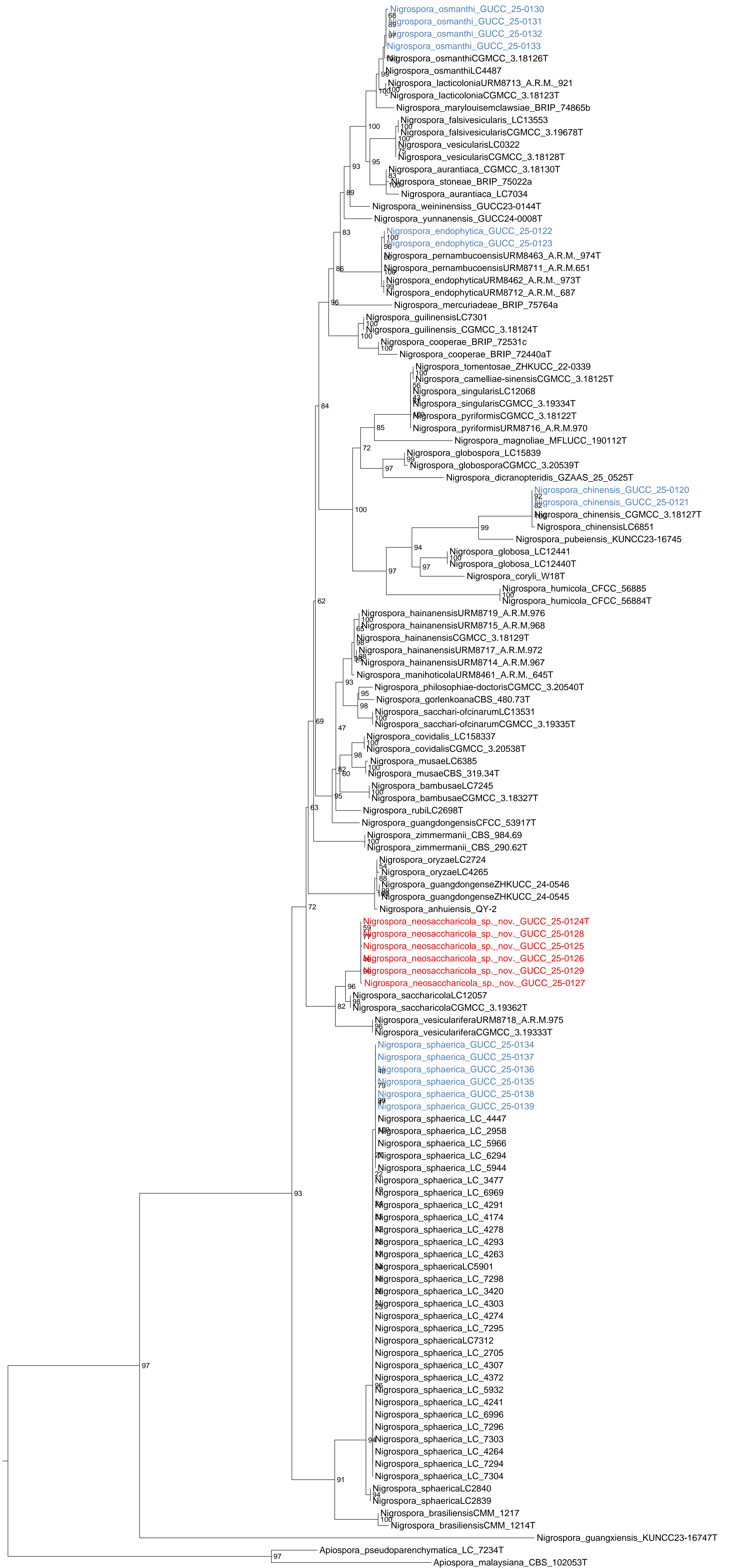

Supplement: Supplementary material 1 — Single gene trees for Apiospora and Nigrospora [file imafungus-17-e177246-s001.zip › Supplementary figure 7 Nigrospora tub ML.pdf]

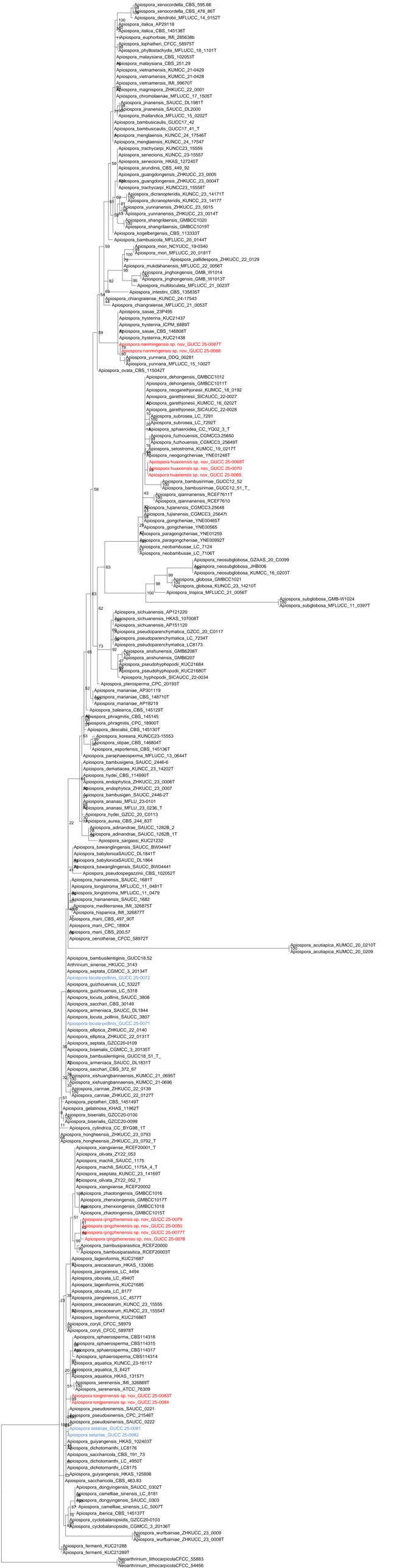

Supplement: Supplementary material 1 — Single gene trees for Apiospora and Nigrospora [file imafungus-17-e177246-s001.zip › Supplementary figure 2 Apiospora LSU ML.pdf]

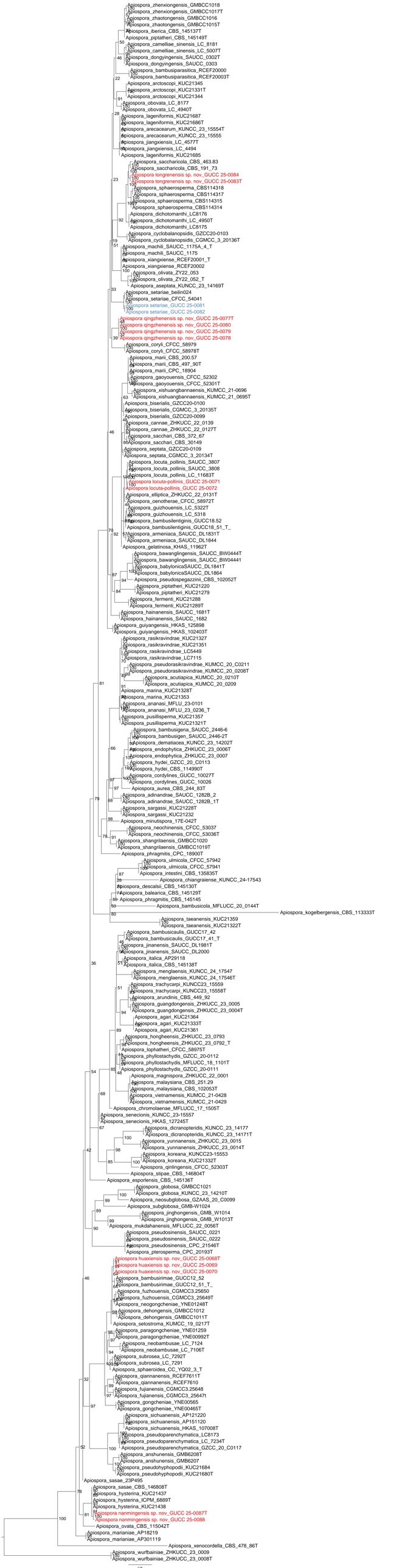

Supplement: Supplementary material 1 — Single gene trees for Apiospora and Nigrospora [file imafungus-17-e177246-s001.zip › Supplementary figure 3 Apiospora tef1-α ML.pdf]
