# Supplementary material for: Novel species and new host records of Apiospora and Nigrospora (Amphisphaeriales, Apiosporaceae) from Yunnan-Guizhou Plateau, China
Source: IMA Fungus. 2026 Apr 24;17:e177246. doi: 10.3897/imafungus.17.177246 (PMC13135200; doi:10.3897/imafungus.17.177246)
Supplement: Supplementary material 1 — Single gene trees for Apiospora and Nigrospora [file Supplementary figure 6 Nigrospora tef1-α ML.pdf › Supplementary figure 6 Nigrospora tef1-α ML.pdf]

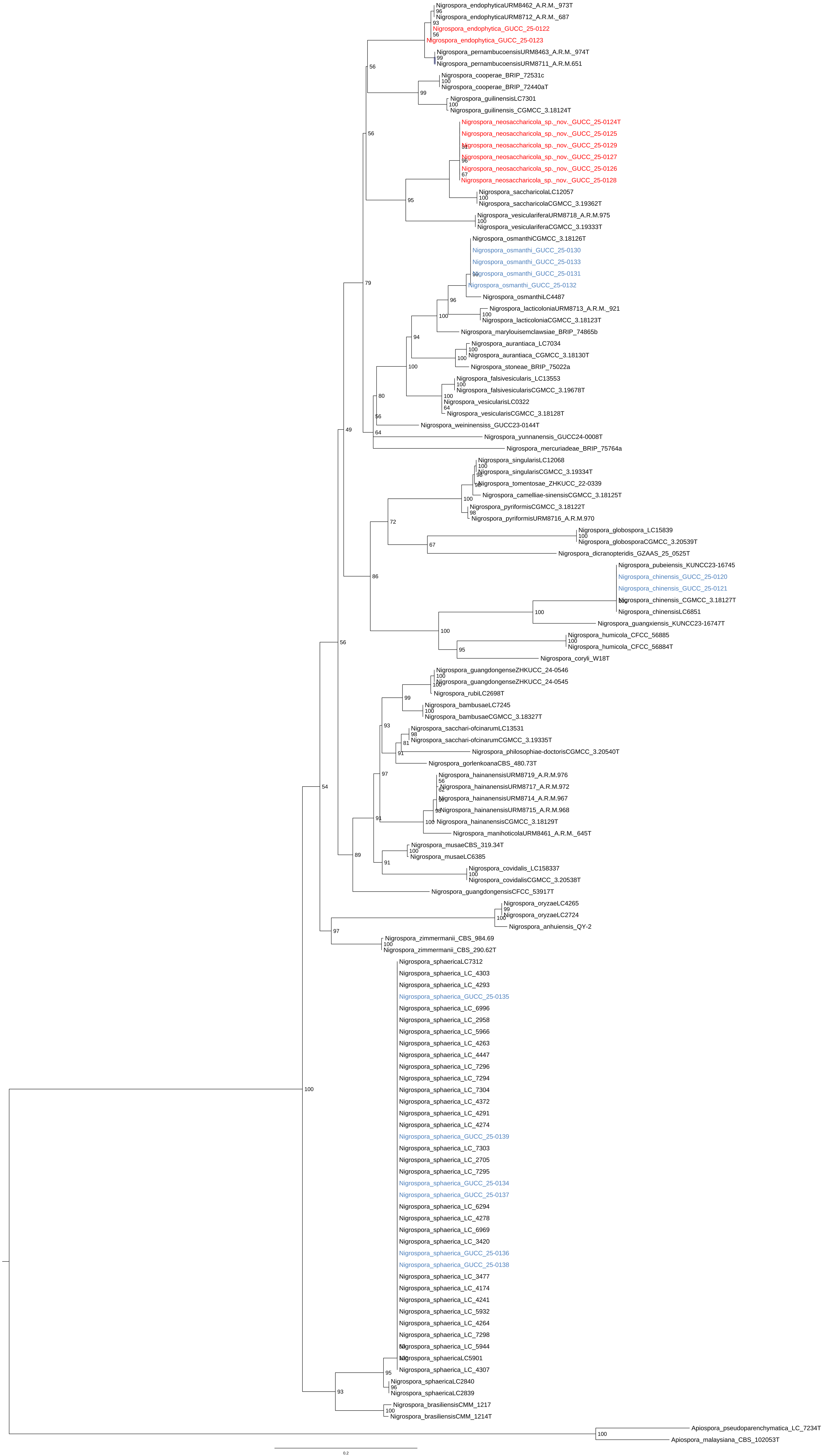
Nigrospora_endophyticaURM8462_A.R.M._973T
Nigrospora_endophyticaURM8712_A.R.M._687
Nigrospora_endophytica_GUCC_25-0122
Nigrospora_endophytica_GUCC_25-0123
Nigrospora_pernambucoensisURM8463_A.R.M._974T
Nigrospora_pernambucoensisURM8711_A.R.M.651
Nigrospora_cooperae_BRIP_72531c
Nigrospora_cooperae_BRIP_72440aT
Nigrospora_guilinensisLC7301
Nigrospora_guilinensis_CGMCC_3.18124T
Nigrospora_neosaccharicola_sp._nov._GUCC_25-0124T
Nigrospora_neosaccharicola_sp._nov._GUCC_25-0125
Nigrospora_neosaccharicola_sp._nov._GUCC_25-0129
Nigrospora_neosaccharicola_sp._nov._GUCC_25-0127
Nigrospora_neosaccharicola_sp._nov._GUCC_25-0126
Nigrospora_neosaccharicola_sp._nov._GUCC_25-0128
Nigrospora_saccharicolaLC12057
Nigrospora_saccharicolaCGMCC_3.19362T
Nigrospora_vesiculariferaURM8718_A.R.M.975
Nigrospora_vesiculariferaCGMCC_3.19333T
Nigrospora_osmanthiCGMCC_3.18126T
Nigrospora_osmanthi_GUCC_25-0130
Nigrospora_osmanthi_GUCC_25-0133
Nigrospora_osmanthi_GUCC_25-0131
Nigrospora_osmanthi_GUCC_25-0132
Nigrospora_osmanthiLC4487
Nigrospora_lacticoloniaURM8713_A.R.M._921
Nigrospora_lacticoloniaCGMCC_3.18123T
Nigrospora_marylouisemclawsiae_BRIP_74865b
Nigrospora_aurantiaca_LC7034
Nigrospora_aurantiaca_CGMCC_3.18130T
Nigrospora_stoneae_BRIP_75022a
Nigrospora_falsivesicularis_LC13553
Nigrospora_falsivesicularisCGMCC_3.19678T
Nigrospora_vesicularisLC0322
Nigrospora_vesicularisCGMCC_3.18128T
Nigrospora_weininensiss_GUCC23-0144T
Nigrospora_yunnanensis_GUCC24-0008T
Nigrospora_mercuriadeae_BRIP_75764a
Nigrospora_singularisLC12068
Nigrospora_singularisCGMCC_3.19334T
Nigrospora_tomentosae_ZHKUCC_22-0339
Nigrospora_camelliae-sinensisCGMCC_3.18125T
Nigrospora_pyriformisCGMCC_3.18122T
Nigrospora_pyriformisURM8716_A.R.M.970
Nigrospora_globospora_LC15839
Nigrospora_globosporaCGMCC_3.20539T
Nigrospora_dicranopteridis_GZAAS_25_0525T
Nigrospora_pubeiensis_KUNCC23-16745
Nigrospora_chinensis_GUCC_25-0120
Nigrospora_chinensis_GUCC_25-0121
Nigrospora_chinensis_CGMCC_3.18127T
Nigrospora_chinensisLC6851
Nigrospora_guangxiensis_KUNCC23-16747T
Nigrospora_humicola_CFCC_56885
Nigrospora_humicola_CFCC_56884T
Nigrospora_coryli_W18T
Nigrospora_guangdongenseZHKUCC_24-0546
Nigrospora_guangdongenseZHKUCC_24-0545
Nigrospora_rubiLC2698T
Nigrospora_bambusaeLC7245
Nigrospora_bambusaeCGMCC_3.18327T
Nigrospora_sacchari-ofcinarumLC13531
Nigrospora_sacchari-ofcinarumCGMCC_3.19335T
Nigrospora_philosophiae-doctorisCGMCC_3.20540T
Nigrospora_gorlenkoanaCBS_480.73T
Nigrospora_hainanensisURM8719_A.R.M.976
Nigrospora_hainanensisURM8717_A.R.M.972
Nigrospora_hainanensisURM8714_A.R.M.967
Nigrospora_hainanensisURM8715_A.R.M.968
Nigrospora_hainanensisCGMCC_3.18129T
Nigrospora_manihoticolaURM8461_A.R.M._645T
Nigrospora_musaeCBS_319.34T
Nigrospora_musaeLC6385
Nigrospora_covidalis_LC158337
Nigrospora_covidalisCGMCC_3.20538T
Nigrospora_guangdongensisCFCC_53917T
Nigrospora_oryzaeLC4265
Nigrospora_oryzaeLC2724
Nigrospora_anhuiensis_QY-2
Nigrospora_zimmermanii_CBS_984.69
Nigrospora_zimmermanii_CBS_290.62T
Nigrospora_sphaericaLC7312
Nigrospora_sphaerica_LC_4303
Nigrospora_sphaerica_LC_4293
Nigrospora_sphaerica_GUCC_25-0135
Nigrospora_sphaerica_LC_6996
Nigrospora_sphaerica_LC_2958
Nigrospora_sphaerica_LC_5966
Nigrospora_sphaerica_LC_4263
Nigrospora_sphaerica_LC_4447
Nigrospora_sphaerica_LC_7296
Nigrospora_sphaerica_LC_7294
Nigrospora_sphaerica_LC_7304
Nigrospora_sphaerica_LC_4372
Nigrospora_sphaerica_LC_4291
Nigrospora_sphaerica_LC_4274
Nigrospora_sphaerica_GUCC_25-0139
Nigrospora_sphaerica_LC_7303
Nigrospora_sphaerica_LC_2705
Nigrospora_sphaerica_LC_7295
Nigrospora_sphaerica_GUCC_25-0134
Nigrospora_sphaerica_GUCC_25-0137
Nigrospora_sphaerica_LC_6294
Nigrospora_sphaerica_LC_4278
Nigrospora_sphaerica_LC_6969
Nigrospora_sphaerica_LC_3420
Nigrospora_sphaerica_GUCC_25-0136
Nigrospora_sphaerica_GUCC_25-0138
Nigrospora_sphaerica_LC_3477
Nigrospora_sphaerica_LC_4174
Nigrospora_sphaerica_LC_4241
Nigrospora_sphaerica_LC_5932
Nigrospora_sphaerica_LC_4264
Nigrospora_sphaerica_LC_7298
Nigrospora_sphaerica_LC_5944
Nigrospora_sphaericaLC5901
Nigrospora_sphaerica_LC_4307
Nigrospora_sphaericaLC2840
Nigrospora_sphaericaLC2839
Nigrospora_brasiliensisCMM_1217
Nigrospora_brasiliensisCMM_1214T
Apiospora_pseudoparenchymatica_LC_7234T
Apiospora_malaysiana_CBS_102053T
0.2
