# Supplementary material for: Novel species and new host records of Apiospora and Nigrospora (Amphisphaeriales, Apiosporaceae) from Yunnan-Guizhou Plateau, China
Source: IMA Fungus. 2026 Apr 24;17:e177246. doi: 10.3897/imafungus.17.177246 (PMC13135200; doi:10.3897/imafungus.17.177246)
Supplement: Supplementary material 1 — Single gene trees for Apiospora and Nigrospora [file Supplementary figure 1 Apiospora ITS ML.pdf › Supplementary figure 1 Apiospora ITS ML.pdf]

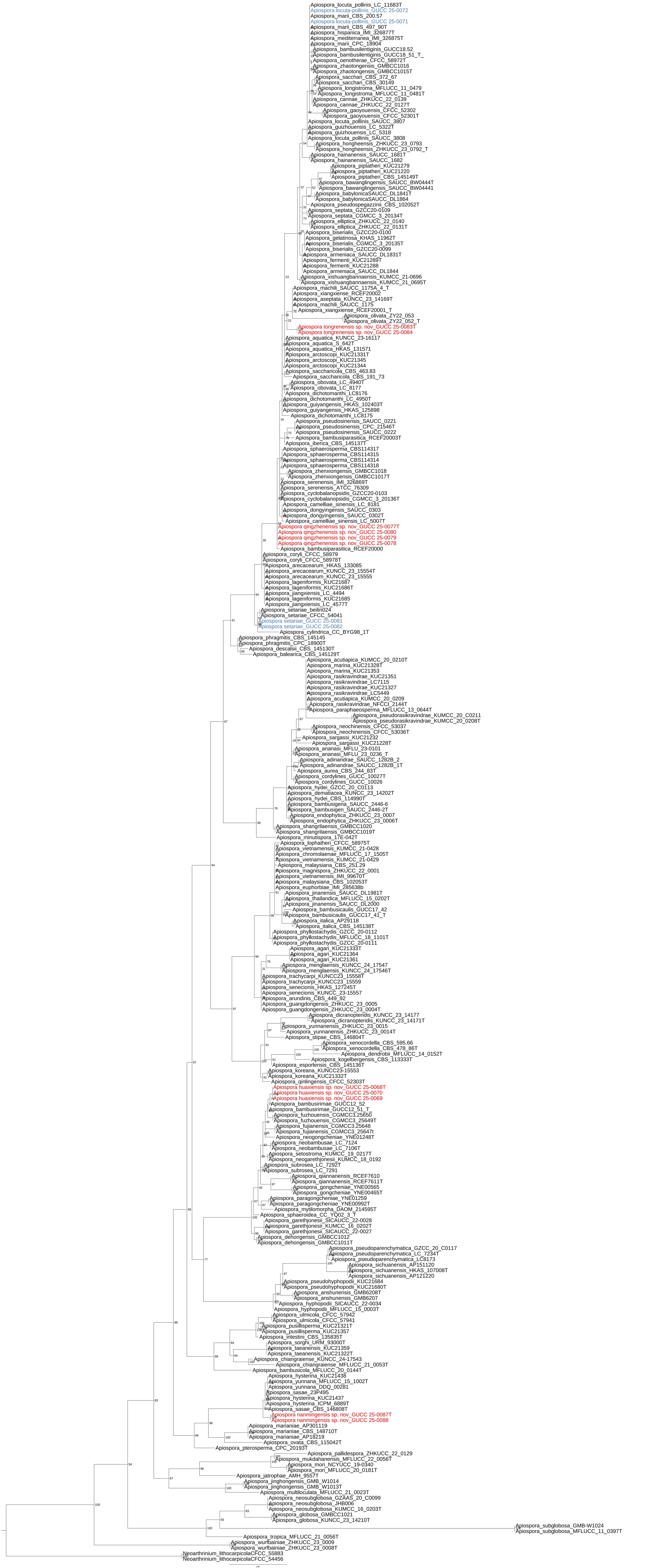

Apiospora_locuta_pollinis_LC_11683T
Apiospora locuta-pollinis_GUCC 25-0072
Apiospora_marii_CBS_200.57
Apiospora locuta-pollinis_GUCC 25-0071
Apiospora_marii_CBS_497_90T
Apiospora_hispanica_IMI_326877T
Apiospora_mediterranea_IMI_326875T
Apiospora_marii_CPC_18904
Apiospora_bambusilentiginis_GUCC18.52
Apiospora_bambusilentiginis_GUCC18_51_T_
Apiospora_oenotherae_CFCC_58972T
Apiospora_zhaotongensis_GMBCC1016
Apiospora_zhaotongensis_GMBCC1015T
Apiospora_sacchari_CBS_372_67
Apiospora_sacchari_CBS_30149
Apiospora_longistroma_MFLUCC_11_0479
Apiospora_longistroma_MFLUCC_11_0481T
Apiospora_cannae_ZHKUCC_22_0139
Apiospora_cannae_ZHKUCC_22_0127T
Apiospora_gaoyouensis_CFCC_52302
Apiospora_gaoyouensis_CFCC_52301T
Apiospora_locuta_pollinis_SAUCC_3807
Apiospora_guizhouensis_LC_5322T
Apiospora_guizhouensis_LC_5318
Apiospora_locuta_pollinis_SAUCC_3808
Apiospora_hongheensis_ZHKUCC_23_0793
Apiospora_hongheensis_ZHKUCC_23_0792_T
Apiospora_hainanensis_SAUCC_1681T
Apiospora_hainanensis_SAUCC_1682
Apiospora_piptatheri_KUC21279
Apiospora_piptatheri_KUC21220
Apiospora_piptatheri_CBS_145149T
Apiospora_bawanglingensis_SAUCC_BW0444T
Apiospora_bawanglingensis_SAUCC_BW04441
Apiospora_babylonicaSAUCC_DL1841T
Apiospora_babylonicaSAUCC_DL1864
Apiospora_pseudospegazzinii_CBS_102052T
Apiospora_septata_GZCC20-0109
Apiospora_septata_CGMCC_3_20134T
Apiospora_elliptica_ZHKUCC_22_0140
Apiospora_elliptica_ZHKUCC_22_0131T
Apiospora_biserialis_GZCC20-0100
Apiospora_gelatinosa_KHAS_11962T
Apiospora_biserialis_CGMCC_3_20135T
Apiospora_biserialis_GZCC20-0099
Apiospora_armeniaca_SAUCC_DL1831T
Apiospora_fermenti_KUC21289T
Apiospora_fermenti_KUC21288
Apiospora_armeniaca_SAUCC_DL1844
Apiospora_xishuangbannaensis_KUMCC_21-0696
Apiospora_xishuangbannaensis_KUMCC_21_0695T
Apiospora_machili_SAUCC_1175A_4_T
Apiospora_xiangxiense_RCEF20002
Apiospora_aseptata_KUNCC_23_14169T
Apiospora_machili_SAUCC_1175
Apiospora_xiangxiense_RCEF20001_T
Apiospora_olivata_ZY22_053
Apiospora_olivata_ZY22_052_T
Apiospora tongrenensis sp. nov_GUCC 25-0083T
Apiospora tongrenensis sp. nov_GUCC 25-0084
Apiospora_aquatica_KUNCC_23-16117
Apiospora_aquatica_S_642T
Apiospora_aquatica_HKAS_131571
Apiospora_arctoscopi_KUC21331T
Apiospora_arctoscopi_KUC21345
Apiospora_arctoscopi_KUC21344
Apiospora_saccharicola_CBS_463.83
Apiospora_saccharicola_CBS_191_73
Apiospora_obovata_LC_4940T
Apiospora_obovata_LC_8177
Apiospora_dichotomanthi_LC8176
Apiospora_dichotomanthi_LC_4950T
Apiospora_guiyangensis_HKAS_102403T
Apiospora_guiyangensis_HKAS_125898
Apiospora_dichotomanthi_LC8175
Apiospora_pseudosinensis_SAUCC_0221
Apiospora_pseudosinensis_CPC_21546T
Apiospora_pseudosinensis_SAUCC_0222
Apiospora_bambusiparasitica_RCEF20003T
Apiospora_iberica_CBS_145137T
Apiospora_sphaerosperma_CBS114317
Apiospora_sphaerosperma_CBS114315
Apiospora_sphaerosperma_CBS114314
Apiospora_sphaerosperma_CBS114318
Apiospora_zhenxiongensis_GMBCC1018
Apiospora_zhenxiongensis_GMBCC1017T
Apiospora_serenensis_IMI_326869T
Apiospora_serenensis_ATCC_76309
Apiospora_cyclobalanopsidis_GZCC20-0103
Apiospora_cyclobalanopsidis_CGMCC_3_20136T
Apiospora_camelliae_sinensis_LC_8181
Apiospora_dongyingensis_SAUCC_0303
Apiospora_dongyingensis_SAUCC_0302T
Apiospora_camelliae_sinensis_LC_5007T
Apiospora qingzhenensis sp. nov_GUCC 25-0077T
Apiospora qingzhenensis sp. nov_GUCC 25-0080
Apiospora qingzhenensis sp. nov_GUCC 25-0079
Apiospora qingzhenensis sp. nov_GUCC 25-0078
Apiospora_bambusiparasitica_RCEF20000
Apiospora_coryli_CFCC_58979
Apiospora_coryli_CFCC_58978T
Apiospora_arecacearum_HKAS_133085
Apiospora_arecacearum_KUNCC_23_15554T
Apiospora_arecacearum_KUNCC_23_15555
Apiospora_lageniformis_KUC21687
Apiospora_lageniformis_KUC21686T
Apiospora_jiangxiensis_LC_4494
Apiospora_lageniformis_KUC21685
Apiospora_jiangxiensis_LC_4577T
Apiospora_setariae_beilin024
Apiospora_setariae_CFCC_54041
Apiospora setariae_GUCC 25-0081
Apiospora setariae_GUCC 25-0082
Apiospora_cylindrica_CC_BYG98_1T
Apiospora_phragmitis_CBS_145145
Apiospora_phragmitis_CPC_18900T
Apiospora_descalsii_CBS_145130T
Apiospora_balearica_CBS_145129T
Apiospora_acutiapica_KUMCC_20_0210T
Apiospora_marina_KUC21328T
Apiospora_marina_KUC21353
Apiospora_rasikravindrae_KUC21351
Apiospora_rasikravindrae_LC7115
Apiospora_rasikravindrae_KUC21327
Apiospora_rasikravindrae_LC5449
Apiospora_acutiapica_KUMCC_20_0209
Apiospora_rasikravindrae_NFCCI_2144T
Apiospora_paraphaeosperma_MFLUCC_13_0644T
Apiospora_pseudorasikravindrae_KUMCC_20_C0211
Apiospora_pseudorasikravindrae_KUMCC_20_0208T
Apiospora_neochinensis_CFCC_53037
Apiospora_neochinensis_CFCC_53036T
Apiospora_sargassi_KUC21232
Apiospora_sargassi_KUC21228T
Apiospora_ananasi_MFLU_23-0101
Apiospora_ananasi_MFLU_23_0236_T
Apiospora_adinandrae_SAUCC_1282B_2
Apiospora_adinandrae_SAUCC_1282B_1T
Apiospora_aurea_CBS_244_83T
Apiospora_cordylines_GUCC_10027T
Apiospora_cordylines_GUCC_10026
Apiospora_hydei_GZCC_20_C0113
Apiospora_dematiacea_KUNCC_23_14202T
Apiospora_hydei_CBS_114990T
Apiospora_bambusigena_SAUCC_2446-6
Apiospora_bambusigen_SAUCC_2446-2T
Apiospora_endophytica_ZHKUCC_23_0007
Apiospora_endophytica_ZHKUCC_23_0006T
Apiospora_shangrilaensis_GMBCC1020
Apiospora_shangrilaensis_GMBCC1019T
Apiospora_minutispora_17E-042T
Apiospora_lophatheri_CFCC_58975T
Apiospora_vietnamensis_KUMCC_21-0428
Apiospora_chromolaenae_MFLUCC_17_1505T
Apiospora_vietnamensis_KUMCC_21-0429
Apiospora_malaysiana_CBS_251.29
Apiospora_magnispora_ZHKUCC_22_0001
Apiospora_vietnamensis_IMI_99670T
Apiospora_malaysiana_CBS_102053T
Apiospora_euphorbiae_IMI_285638b
Apiospora_jinanensis_SAUCC_DL1981T
Apiospora_thailandica_MFLUCC_15_0202T
Apiospora_jinanensis_SAUCC_DL2000
Apiospora_bambusicaulis_GUCC17_42
Apiospora_bambusicaulis_GUCC17_41_T
Apiospora_italica_AP29118
Apiospora_italica_CBS_145138T
Apiospora_phyllostachydis_GZCC_20-0112
Apiospora_phyllostachydis_MFLUCC_18_1101T
Apiospora_phyllostachydis_GZCC_20-0111
Apiospora_agari_KUC21333T
Apiospora_agari_KUC21364
Apiospora_agari_KUC21361
Apiospora_menglaensis_KUNCC_24_17547
Apiospora_menglaensis_KUNCC_24_17546T
Apiospora_trachycarpi_KUNCC23_15558T
Apiospora_trachycarpi_KUNCC23_15559
Apiospora_senecionis_HKAS_127245T
Apiospora_senecionis_KUNCC_23-15557
Apiospora_arundinis_CBS_449_92
Apiospora_guangdongensis_ZHKUCC_23_0005
Apiospora_guangdongensis_ZHKUCC_23_0004T
Apiospora_dicranopteridis_KUNCC_23_14177
Apiospora_dicranopteridis_KUNCC_23_14171T
Apiospora_yunnanensis_ZHKUCC_23_0015
Apiospora_yunnanensis_ZHKUCC_23_0014T
Apiospora_stipae_CBS_146804T
Apiospora_xenocordella_CBS_595.66
Apiospora_xenocordella_CBS_478_86T
Apiospora_dendrobii_MFLUCC_14_0152T
Apiospora_kogelbergensis_CBS_113333T
Apiospora_esporlensis_CBS_145136T
Apiospora_koreana_KUNCC23-15553
Apiospora_koreana_KUC21332T
Apiospora_qinlingensis_CFCC_52303T
Apiospora huaxiensis sp. nov_GUCC 25-0068T
Apiospora huaxiensis sp. nov_GUCC 25-0070
Apiospora huaxiensis sp. nov_GUCC 25-0069
Apiospora_bambusirimae_GUCC12_52
Apiospora_bambusirimae_GUCC12_51_T
Apiospora_fuzhouensis_CGMCC3.25650
Apiospora_fuzhouensis_CGMCC3_25649T
Apiospora_fujianensis_CGMCC3.25648
Apiospora_fujianensis_CGMCC3_25647t
Apiospora_neogongcheniae_YNE01248T
Apiospora_neobambusae_LC_7124
Apiospora_neobambusae_LC_7106T
Apiospora_setostroma_KUMCC_19_0217T
Apiospora_neogarethjonesii_KUMCC_18_0192
Apiospora_subrosea_LC_7292T
Apiospora_subrosea_LC_7291
Apiospora_qiannanensis_RCEF7610
Apiospora_qiannanensis_RCEF7611T
Apiospora_gongcheniae_YNE00565
Apiospora_gongcheniae_YNE00465T
Apiospora_paragongcheniae_YNE01259
Apiospora_paragongcheniae_YNE00992T
Apiospora_mytilomorpha_DAOM_214595T
Apiospora_sphaeroidea_CC_YQ02_3_T
Apiospora_garethjonesii_SICAUCC_22-0028
Apiospora_garethjonesii_KUMCC_16_0202T
Apiospora_garethjonesii_SICAUCC_22-0027
Apiospora_dehongensis_GMBCC1012
Apiospora_dehongensis_GMBCC1011T
Apiospora_pseudoparenchymatica_GZCC_20_C0117
Apiospora_pseudoparenchymatica_LC_7234T
Apiospora_pseudoparenchymatica_LC8173
Apiospora_sichuanensis_AP151120
Apiospora_sichuanensis_HKAS_107008T
Apiospora_sichuanensis_AP121220
Apiospora_pseudohyphopodii_KUC21684
Apiospora_pseudohyphopodii_KUC21680T
Apiospora_anshunensis_GMB6208T
Apiospora_anshunensis_GMB6207
Apiospora_hyphopodii_SICAUCC_22-0034
Apiospora_hyphopodii_MFLUCC_15_0003T
Apiospora_ulmicola_CFCC_57942
Apiospora_ulmicola_CFCC_57941
Apiospora_pusillisperma_KUC21321T
Apiospora_pusillisperma_KUC21357
Apiospora_intestini_CBS_135835T
Apiospora_sorghi_URM_93000T
Apiospora_taeanensis_KUC21359
Apiospora_taeanensis_KUC21322T
Apiospora_chiangraiense_KUNCC_24-17543
Apiospora_chiangraiense_MFLUCC_21_0053T
Apiospora_bambusicola_MFLUCC_20_0144T
Apiospora_hysterina_KUC21438
Apiospora_yunnana_MFLUCC_15_1002T
Apiospora_yunnana_DDQ_00281
Apiospora_sasae_23P495
Apiospora_hysterina_KUC21437
Apiospora_hysterina_ICPM_6889T
Apiospora_sasae_CBS_146808T
Apiospora nanmingensis sp. nov_GUCC 25-0087T
Apiospora nanmingensis sp. nov_GUCC 25-0088
Apiospora_marianiae_AP301119
Apiospora_marianiae_CBS_148710T
Apiospora_marianiae_AP18219
Apiospora_ovata_CBS_115042T
Apiospora_pterosperma_CPC_20193T
Apiospora_pallidespora_ZHKUCC_22_0129
Apiospora_mukdahanensis_MFLUCC_22_0056T
Apiospora_mori_NCYUCC_19-0340
Apiospora_mori_MFLUCC_20_0181T
Apiospora_jatrophae_AMH_9557T
Apiospora_jinghongensis_GMB_W1014
Apiospora_jinghongensis_GMB_W1013T
Apiospora_multiloculata_MFLUCC_21_0023T
Apiospora_neosubglobosa_GZAAS_20_C0099
Apiospora_neosubglobosa_JHB006
Apiospora_neosubglobosa_KUMCC_16_0203T
Apiospora_globosa_GMBCC1021
Apiospora_globosa_KUNCC_23_14210T
Apiospora_subglobosa_GMB-W1024
Apiospora_subglobosa_MFLUCC_11_0397T
Apiospora_tropica_MFLUCC_21_0056T
Apiospora_wurfbainiae_ZHKUCC_23_0009
Apiospora_wurfbainiae_ZHKUCC_23_0008T
Neoarthrinium_lithocarpicolaCFCC_55883
Neoarthrinium_lithocarpicolaCFCC_54456
